# Classifying polish in use-wear analysis with convolutional neural networks

**DOI:** 10.1038/s41598-025-18179-4

**Published:** 2025-10-22

**Authors:** Anastasia Eleftheriadou, Youssef Djellal, Shannon P. McPherron, João Marreiros

**Affiliations:** 1 Interdisciplinary Center for Archaeology and the Evolution of Human Behaviour (ICArEHB), Universidade do Algarve, Faro, Portugal; 2 TraCEr, Laboratory for Traceology and Controlled Experiments at MONREPOS Archaeological Research Centre and Museum for Human Behavioural Evolution, The Leibniz-Zentrum für Archäologie (LEIZA), Schloss Monrepos, 56567 Neuwied, Germany; 3 Department of Human Origins, Max Planck Institute for Evolutionary Anthropology, Deutscher Platz 6, 04103 Leipzig, Germany

**Keywords:** Lithic use-wear analysis, Polish classification, Convolutional neural networks, Experimental archaeology, Deep learning, Archaeology, Computational science

## Abstract

Lithic use-wear analysis examines micro- and macroscopic traces on tool surfaces resulting from human use and post-depositional processes. Polish, formed through surface abrasion with different materials, is a key diagnostic feature that is increasingly analyzed using machine learning to enhance automation and standardization. However, further research is needed to explore whether deep learning approaches, in particular, can be effectively applied to use-wear analysis and to determine the optimal surface area size (e.g., patch size and microscope objectives) and model architecture (custom vs. pre-trained) for achieving the best results. This study employs convolutional neural networks (CNNs) to classify experimental polish based on contact material (wood, hide, bone) and use intensity, while also assessing optimal imaging and analytical parameters. The results of this exploratory study suggest that CNNs may effectively identify polish from bone and hide but perform less effectively with wood. The models also successfully distinguish between polish formed by short- and long-term use. Custom models outperformed pre-trained ones, particularly when using images that captured smaller areas of the tool’s surface, suggesting that bigger surface areas may lack the necessary information for optimal results. These findings underscore the need to expand use-wear datasets in terms of size and variability and optimize CNN architectures and workflows.

**Supplementary Information:**

The online version contains supplementary material available at 10.1038/s41598-025-18179-4.

## Introduction

A central theme in human evolution studies is understanding how technological traditions and innovations enabled hominins to survive and disperse across continents^1^. Stone artifacts play a pivotal role in this discussion by offering valuable insights into subsistence strategies, cultural transmission, and cognitive abilities^2^. Use-wear analysis, developed by Semenov in the 1960s, is a fundamental method for investigating tool function by examining macroscopic and microscopic trace patterns left on tool surfaces after use^3–5^. Experimental archaeology further enriches this approach by creating reference libraries of tool traces against which archaeological artifacts can be compared^6^. Recently, controlled experiments have helped to establish more systematic, repeatable, and reproducible workflows by implementing hypothesis-driven experimental designs and using mechanical or automated instruments to isolate, control, and measure specific variables^7^.

One of the most extensively studied traces in use-wear analysis is polish, which refers to the modified area of a tool that appears brighter or smoother due to the gradual removal or deformation of the natural surface caused by physical abrasion during contact with other materials^8,9^. Polish formation is a dynamic process influenced by factors such as tool material, contact material, and duration of use^10^. Traditionally, polish classification relied on qualitative assessments, where analysts visually identified and interpreted features^8^. However, these methods are prone to intra- and inter-analyst errors and have demonstrated limited performance in blind tests^11^. To address these issues, researchers have introduced quantitative approaches for polish identification, such as surface texture analysis^12^, gray-level co-occurrence matrix (GLCM)^13^, and fractal analysis^14^.

Recent efforts have sought to improve the classification of use-wear traces by applying quantitative methods ranging from traditional statistical approaches to more advanced machine learning (ML) techniques, to either tabular or image data^15^. Statistical methods such as quadratic discriminant function analysis (QDA)^10,16^, discriminant analysis (DA)^17,18^, and logistic regression^19^ have been used to classify polish based on the contact material and wear patterns. Although these approaches have provided valuable insights, they often require predefined assumptions regarding data distributions and feature selection, which can limit their adaptability to complex patterns of use-wear traces^20^.

In contrast, ML, a subset of artificial intelligence (AI) that enables algorithms to learn patterns from data and improve their performance over time, offers a more robust approach^21^. ML has been used to classify use-wear based on various factors, including contact material hardness (e.g., soft vs. hard), type (e.g., bone vs. hide), and condition (e.g., dry vs. fresh)^11^. Overall, ML has shown promising results, achieving up to 78% accuracy in classifying different contact materials^13^. However, most studies reported lower classification performance, with overall model accuracies of 67%^22^, 60%^18^, and 47%^11^. The difficulty in achieving better results can be attributed to several factors, including equifinality (i.e., different materials or activities exhibiting similar polish characteristics), post-depositional processes (in the case of archaeological specimens), limitations in dataset size and quality, and the selection of inadequate ML algorithms^11,17,22,23^. Another aspect explored is how machine learning models perform compared to blind tests, where analysts classify use-wear samples without knowing which material group they belong to^18,22^. While some models classify polish as accurately as, or better than, human experts, scholars emphasize that expert knowledge is essential not only to validate the results but also to ensure that the models base their decisions on meaningful features of use-wear polish^18,22^. A growing body of research has examined the different methodological factors influencing ML performance in use-wear analysis. For instance, Stevens et al.^18^ compared DA and decision trees (DT), Pedergnana et al.^11^ compared support vector machines (SVM) and DT, and Sferrazza^13^ compared SVM and random forest (RF). Others have investigated the effectiveness of various micro surface texture analysis techniques, including ISO 25178-2, scale-sensitive fractal analysis, and GLCM, to determine which best captures polish characteristics^11^.

More recently, deep learning methods, particularly convolutional neural networks (CNNs), have shown promise in automating the classification of polish by directly analyzing image data^22^. CNNs are a type of neural network used to learn the underlying spatial features in images and utilize this information for segmentation and classification tasks^24^. Compared to other machine learning approaches, CNNs directly process raw pixel data to simultaneously learn hierarchical feature representations and perform classification, facilitating the detection of complex patterns characteristic of lithic use-wear^22,25^. Despite their potential, research on CNNs for use-wear analysis remains limited. To date, Zhang et al.^22^ is the only study that systematically explores CNNs for polish classification, testing different models (ResNet50, ResNet152, ConvNeXt, and ViT-H) training strategies (e.g., pre-trained vs. custom-trained models), image resolutions, and sensing modalities (2D vs. 3D). However, critical questions remain, including whether CNNs can effectively classify polish images in response to different research questions, which model architectures are best suited for various polish types, and how data acquisition and preprocessing parameters impact CNN performance.

This study aims to address this gap by identifying optimal acquisition settings and analysis parameters for the classification of use-wear images using CNNs. Specifically, the main goal is to classify images of experimental polish based on the contact material and number of strokes by evaluating two key factors: (1) the impact of the ‘window of analysis’ referring to the size of the area captured in an image and (2) the suitability of custom-trained versus pre-trained models. The first factor examines how the scale at which polish is observed affects classification (i.e., larger surface areas may capture more extensive wear, while smaller areas may reflect more localized wear), which is quantified by the dimensions of image segments (patch size) and the choice of microscope objective. The second factor evaluates whether a commonly used pre-trained model, such as ResNet50, performs better than a model specifically trained on the current dataset. This study provides a heuristic tool to better understand the potential and limitations of CNNs in use-wear analysis, while also presenting one of the first cases to explore the effects of transfer learning and model interpretability. Although the results presented here are preliminary, they offer insights into the influence of acquisition settings and analysis parameters on polish development and classification on flint. The study provides a baseline that, with the availability of larger and more diverse datasets from experimental and archaeological contexts, will support the refinement and integration of computational techniques in material studies and archaeology.

## Results

### Overview of model performance

**Fig. 1:**
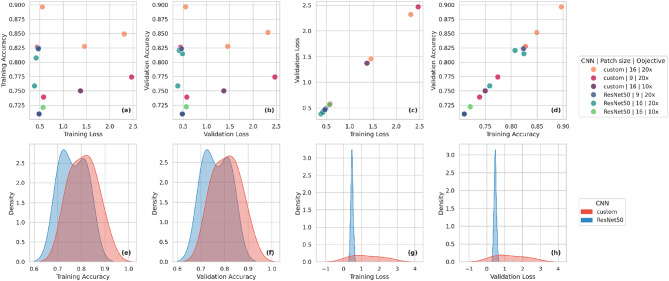
Summary of final accuracy and loss values reached by the models combining different architectures, patch sizes and objectives. Color gradients distinguish model configurations (upper legend): custom CNNs (red-purple) and ResNet50 (blue-green), with hues varying by patch size (9 vs. 16) and objective (10× vs. 20×), (lower legend): custom CNN (red) and ResNet50 (blue). (**a–d**) Scatterplots comparing metrics across training and validation sets: (**a**) training loss vs. training accuracy, (**b**) validation loss vs. validation accuracy, (**c**) training loss vs. validation loss, and (**d**) training accuracy vs. validation accuracy. (**e–h**) Kernel density estimates showing distributions of each metric per model type (**e**) training accuracy, (**f**) validation accuracy, (**g**) training loss, and (**h**) validation loss.

Given the large number of models and parameters involved, Fig. 1 summarizes the accuracy and loss values for both the training and validation sets at the end of each model run. Kernel density estimate (KDE) plots were used to visualize the distribution of values per model type, while scatterplots were used to illustrate the relationships between model type, objective and patch size. The loss and accuracy values for both the training and validation sets were similar for each model (Fig. 1e–h). ResNet50 shows for most models to have validation accuracy ranging from 0.70 to 0.82 (Fig. 1b, f) and validation loss from 0.38 to 0.56 (Fig. 1b, h). The custom CNN shows a similar range of validation accuracy values (0.73–0.89) but with a wider range of validation loss (0.44–2.46) (Fig. 1c, d). When it comes to the performance of the models relative to the loss, all ResNet50 models show minimal to no difference between the training and validation sets (Fig. 1c), with the majority of loss values being approximately 0.5 (Fig. 1c, g). The custom CNN also showed minimal to no difference between the training and validation sets (Fig. 1a–d); however, the distribution of accuracy values among the models was less consistent (Fig. 1a, d).

**Fig. 2:**
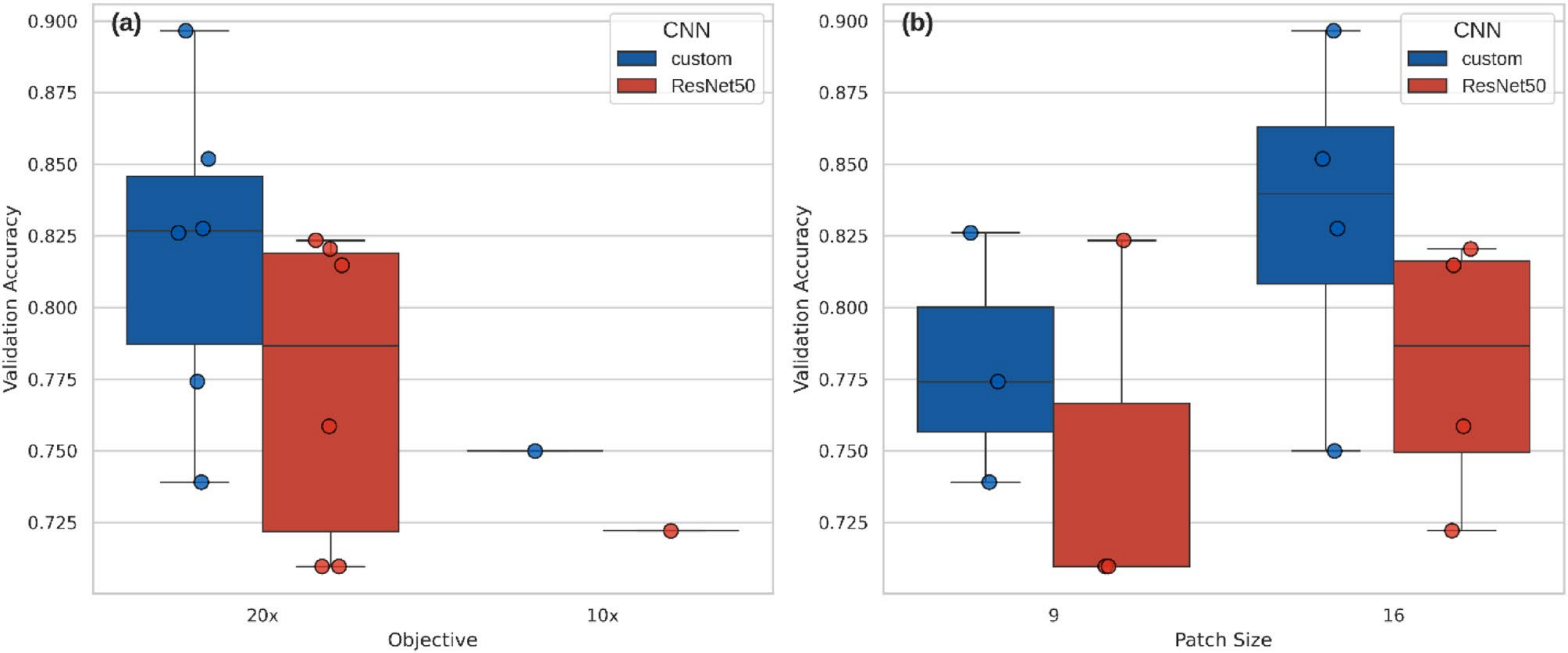
Boxplot with overlaid data points showing the validation accuracy of fourteen CNN models, with custom models in blue and ResNet50 in red, categorized by objective (**a**) and patch size (**b**).

Both models show the same pattern in the effect of objective choice, showing 20x (median accuracy: custom = 0.82, ResNet50 = 0.79) to achieve higher accuracy than 10x (median accuracy for custom = 0.75 and ResNet50 = 0.72) (Fig. 2). The effect of patch size on classification performance differs per model, with the custom CNN showing higher accuracy for 16 (median = 0.83) than 9 (median = 0.77), similar to ResNet50 (patch size 16: median = 0.78, patch size 9: median = 0.71). Overall, the custom CNN exhibited higher median accuracy values for both types of objectives and patch sizes.

**Fig. 3:**
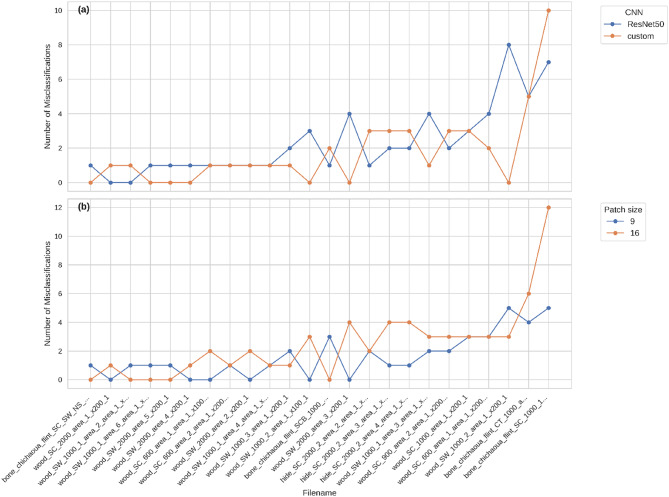
Line plots illustrating the number of misclassifications made by CNN models, categorized by model type (**a**) and patch size (**b**) for each image.

The frequency of misclassifications, defined as instances in which images were incorrectly assigned to a specific contact material or stroke count, was organized by model type and patch size (Fig. 3). For the ResNet50 model, misclassifications occurred as follows: six images were misclassified once, eight images two to four times, and two images more than five times. For the custom CNN, misclassifications were distributed as follows: eight images were misclassified once, seven images two to four times, and two images was misclassified more than five times. When examining the effect of patch size, the dataset divided into nine areas exhibited 6 images misclassified once,6 images 2 to 4 times and 2 images misclassified more than five times. In contrast, the dataset divided into 16 areas showed 5 images misclassified once, 13 images 2 to four times, and 2 images misclassified more than five times. Only five images were misclassified more often, irrespective of model type or patch size, whereas the majority of images exhibited no discernible pattern of recurrent misclassification.

### Learning curves

All learning curves, confusion matrices, and ROC-AUC curves illustrating the performance of the models are available in the Supplementary Fig. S4. Overall, both the custom CNN and ResNet50 exhibited learning curves consistent with the expected pattern of decreasing loss and increasing accuracy for both the training and validation sets. Additionally, in both model types, the curves displayed frequent but generally non-extreme fluctuations.

**Fig. 4:**
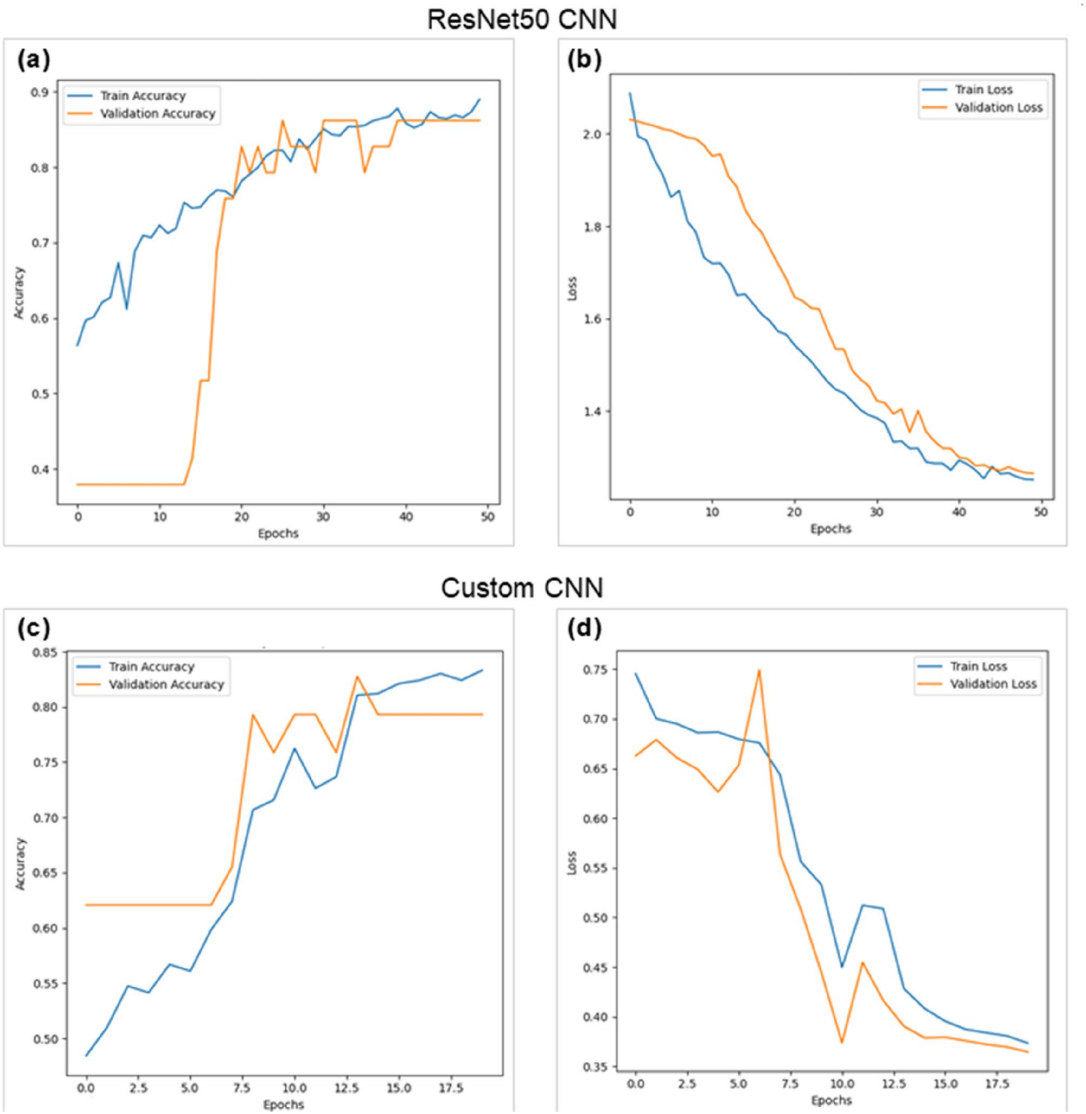
Learning curves of CNN models trained, tested, and validated on a dataset using a 20× objective, 2000 strokes, and a patch size of 16. (**a**) Accuracy and (**b**) loss for the ResNet50 model, and (**c**) accuracy and (**d**) loss for the custom CNN model are displayed.

The trends in accuracy and loss appear to be more stable in the custom CNNs, whereas the ResNet50 models exhibit greater irregularities, characterized by random fluctuations and plateaus (Fig. 4). When present, small discrepancies between training and validation loss are primarily observed in the early stages of training in custom CNNs but tend to diminish as training progresses. ResNet50 models also exhibit limited distance between training and validation loss; however, in some instances, a persistent gap remains throughout the training, preventing their convergence.

A difference is observed in the application of early stopping, with custom CNNs typically training for up to 50 epochs, whereas ResNet50 models terminate earlier, at approximately 17 epochs. Regarding classification performance, confusion matrices indicate that custom CNNs yield false positive and false negative rates between 0.06% and 40% across different classes, whereas ResNet50 models exhibit higher misclassification rates, with some models misclassifying wood in up to 50% of the cases (Supplementary Table S1). In terms of ROC-AUC values, custom CNN models outperformed all ResNet50 counterparts.

### Saliency maps

**Fig. 5:**
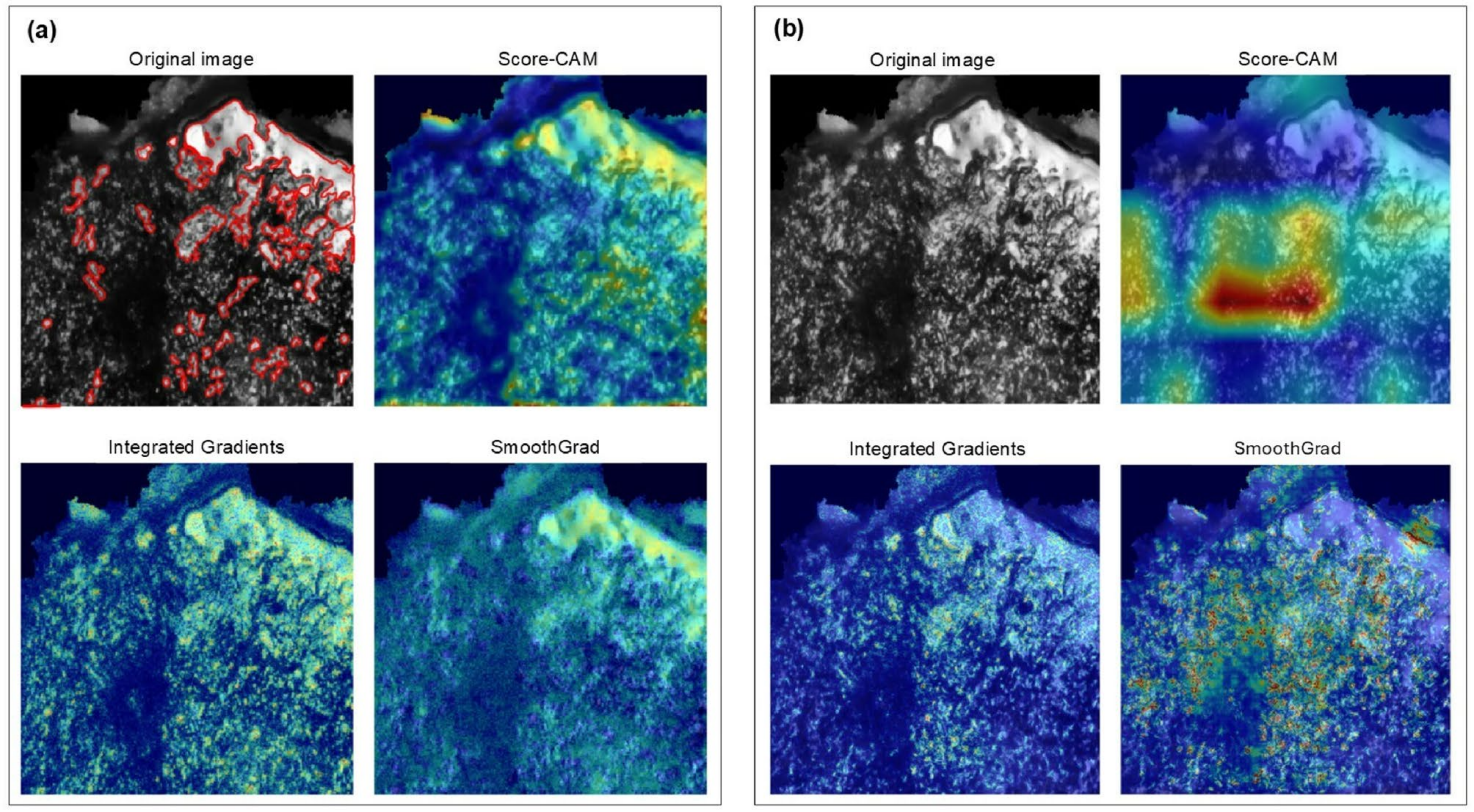
An example of saliency maps generated for an image (224 × 224 pixels) of bone polish acquired with a 10x objective, 1000 strokes, and 16 patches, classified using a custom CNN (**a**) and ResNet50 CNN (**b**). The position of the polish is outlined in the original image (**a**, top left) for reference. Colder colors represent areas with lower activation, indicating regions that the model focuses less on, while warmer colors highlight areas with higher activation, showing regions the model pays more attention to.

The performance of saliency maps varies depending on the model architecture and research question. For the custom CNN, the results produced by Score-CAM, Integrated Gradients, and SmoothGrad were largely consistent with one another, exhibiting minimal variation in color distribution (i.e. which areas were important) and slight differences in color intensity (i.e. how important these areas were) (Fig. 5a). Models classifying polish based on contact material predominantly highlight regions where polish is present and well-defined. In contrast, models classifying based on the number of strokes also emphasize areas with prominent polish, but do not necessarily assign them the highest importance scores. Instead, they tend to incorporate broader areas of the lithic’s surface, sometimes emphasizing regions with more diffuse or weaker polish patterns (e.g., Fig. 5a). In both classification tasks, Score-CAM effectively identified local patterns and provided a smoother distribution of importance across the image.

For ResNet50, the saliency maps exhibit different patterns. Regardless of whether the classification pertains to the contact material or the number of strokes, the highlighted regions do not always correspond to polished areas, nor do they consistently emphasize the most well-developed polish. ResNet50 models typically focus on the central portion of the image, identifying at most one or two regions of importance (Fig. 5b). Integrated Gradients and SmoothGrad tend to capture more localized patterns and are largely in agreement with each other, whereas Score-CAM appears to emphasize global patterns, covering larger portions of the image. In some cases, although Score-CAM aligns with the location of the patterns observed in Integrated Gradients and SmoothGrad, it redistributes the intensity of color in different ways (Fig. 5b).

### Research question 1: material classification

#### Performance across materials

**Fig. 6:**
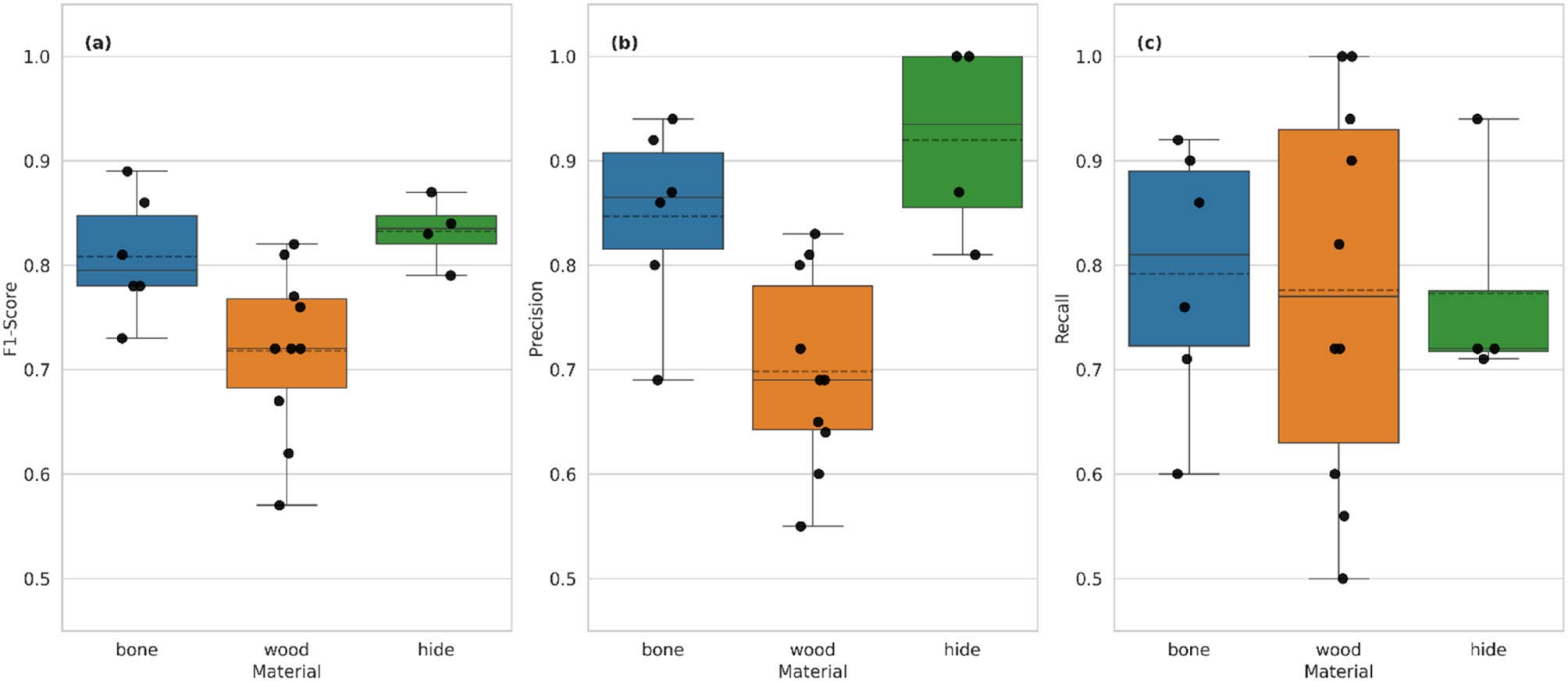
Boxplot comparing the F1-score, precision, and recall values of all CNN models for hide (green), bone (blue), and wood (orange). Data points representing the exact values are overlaid on the plot. The solid line within each box denotes the median, while the dashed line represents the mean value.

Figure 6 compares the classification performance of three material categories—hide, bone, and wood—based on F1-score, precision, and recall. Hide exhibits the highest overall classification performance, with a median F1-score of 0.83 and low variance (0.79–0.87). It also achieved the highest precision (median: 0.94, variance: 0.79–1.00), although its recall was the lowest (mean: 0.73, variance: 0.71–0.93). Bone polish ranks second in classification performance, with a median F1-score of 0.79 (variance: 0.73–0.89), precision of 0.86 (variance: 0.69–0.94), and recall of 0.81 (variance: 0.60–0.92). Wood demonstrated the lowest classification performance with the lowest F1-score (median: 0.74, variance: 0.55–0.84) and precision (median: 0.69, variance: 0.55–0.83). Although wood achieved the second highest recall (median: 0.77), it also exhibited the greatest variance among all materials (0.50–1.00).

### Effect of model, patch size and objective

Supplementary Fig. S1 illustrates the impact of contact material, objective and patch size on polish classification based on model type. Both the custom and ResNet50 CNNs performed similarly, showing hide as the best classified class (custom CNN: median = 0.835, variance = 0.83–0.84, ResNet50: median = 0.83, variance = 0.73–0.87), followed by bone and wood. Overall, the custom CNN showed higher median F1-scores and lower variance for the majority of classes. In terms of the objective, both models agree that 20x (median F1-score: custom = 0.82, ResNet50 = 0.75) is better classified than 10x (median F1-score: custom = 0.75, ResNet50 = 0.70). The median F1-scores are similar for both models, however the custom CNN has considerably lower variance (20x = 0.72–0.89, 10x: 0.74–0.76) than ResNet50 (20x = 0.55–0.86, 10x = 0.62–0.77). The effect of patch size on classification performance differs for each model. The custom CNN shows the division in 9 areas to raise slightly better results (median F1-score: 0.82, variance: 0.72–0.83) than the division in 16 areas (median F1-score: 0.79, variance: 0.73–0.89). ResNet50 shows the opposite pattern with the division in 16 areas to raise better results (median F1-score: 0.75, variance: 0.62–0.86) than the division in 9 areas (median F1-score: 0.73, variance: 0.55–0.82).

### Research question 2: classification based on number of strokes

#### Performance by stroke count

**Fig. 7:**
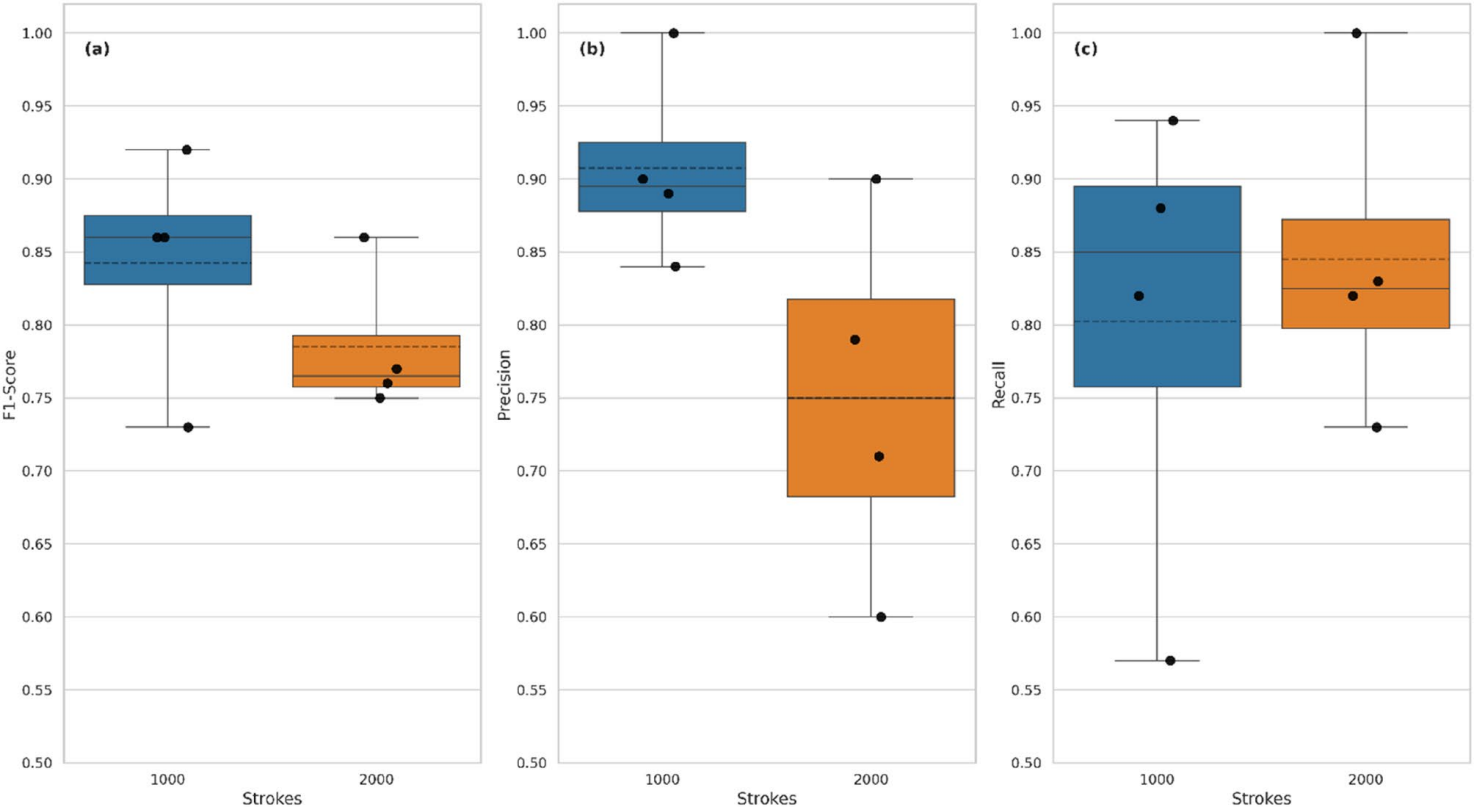
Boxplot comparing the F1-score, precision, and recall values of all CNN models for polish created with 1000 (blue) and 2000 (orange) strokes. Data points representing the exact values are overlaid on the plot. The solid line within each box denotes the median, while the dashed line represents the mean value.

An overview of the classification performance of the different numbers of strokes can be found in Fig. 7. Polish developed with 1000 strokes is the best classified in terms of F1 score (custom median: 0.91, variance: 0.73–0.92), precision (median: 0.89, variance: 0.84-1.00) and recall (mean: 0.85, variance: 0.57–0.94). Polish developed with 2000 strokes exhibits lower median and variance values (F1-score median: 0.76, variance: 0.75–0.86, precision median: 0.75, variance: 0.60–0.90, recall median: 0.83, variance: 0.73-1.00).

### Effect of model and patch size

Supplementary Fig. S2 illustrates the impact of model type and patch size on polish classification based on the number of strokes. In terms of model type, custom CNNs achieve similar median F1-scores (1000 strokes: 0.82; 2000 strokes: 0.81), but exhibit large variance (1000 strokes: 0.73–0.92; 2000 strokes: 0.75–0.86). In comparison, ResNet50 shows smaller variance (1000 strokes: 0.86; 2000 strokes: 0.760–0.765) and higher F1-scores for 1000 strokes. Furthermore, ResNet50 models perform similarly regardless of patch size (9 patches median: 0.815, variance: 0.77–0.86, 16 patches median: 0.81, variance: 0.76–0.86), while custom models show higher F1-scores for 16 patches (median: 0.88; range: 0.855–0.92) compared to 9 patches (median: 0.74; range: 0.73–0.75).

## Discussion

This study demonstrates the potential and challenges of employing CNNs for lithic use wear analysis and polish classification. The primary objective was to develop models capable of distinguishing microscopy images of experimental polish based on the contact material and intensity of use, while also assessing the impact of key parameters such as the size of the ‘window of analysis’ (quantified by the objective and patch size) and model configuration (custom vs. pre-trained).

The custom CNN models demonstrate better performance, as evidenced by the evaluation metrics (i.e., high and consistent values of precision, recall, F1-score, accuracy, and ROC) and interpretability methods (e.g., saliency maps that effectively highlight areas of polish). In comparison, ResNet50 models tended to show lower scores across these metrics. Although there are cases where ResNet50 outperforms custom CNNs in certain areas, such as overall loss values and higher accuracy for specific classes, its overall performance is hindered by two key limitations. First, the learning curves of certain models exhibit more intense and frequent fluctuations, periods of inconsistent learning characterized by short plateaus during training, and instances where training and validation loss curves fail to converge, suggesting potential stagnation in the model’s learning process. Second, the saliency maps produced by ResNet50 exhibit discrepancies across different interpretability methods and predominantly focus on the central region of the image, typically highlighting only one or two areas. This tendency, referred to as center bias, has also been identifying by other scholars and is considered an artifact of transfer learning, resulting from ResNet50’s pretraining on the ImageNet dataset, where images predominantly contain a single, centrally located object of interest^26^.

While these findings indicate that custom CNNs perform well in terms of classification accuracy, learning stability, and interpretability, it is important to note that the observed differences may also be influenced by the limited dataset size and should therefore be interpreted with caution. Although these trends may not reflect statistically significant differences in a strict sense, they are consistent across experiments (see Supplementary Table S2 for more details) and provide a valuable starting point for future research. To the best of our knowledge, previous research using CNNs in use-wear analysis has only employed pre-trained model architectures^22,27^. Therefore, a direct comparison with other studies on the suitability of custom versus pre-trained models is not possible. Additionally, a comparison of ResNet50’s application between these studies and ours is challenging due to methodological differences. For instance, Zhang et al.^22^ used different data types as input (texture scans or heightmaps), so any performance discrepancies may be attributed to variations in data type. Similarly, Sferrazza^27^ used ResNet50 solely for feature map extraction, with classification performed independently by a Multi-Layer Perceptron, thus a comparison is not feasible as ResNet50 was not involved in the classification process.

For an AI system to accurately model and predict the phenomenon it is intended to measure, reliable and representative data is essential^28^. The distribution of misclassified images in our dataset is uniform across classes, with no particular samples consistently predicted incorrectly across models or patch sizes. This suggests that the errors stem from the difficulty of the model in distinguishing between classes rather than flaws in the dataset, which would otherwise cause certain images to be repeatedly misclassified. Only one image of bone polish was consistently misclassified, which may be due to the polish in that area being atypical or not well developed.

The models consistently demonstrate better performance when using images acquired with a 20× objective compared to a 10× objective, likely because the information captured at lower magnifications is insufficient for the polish patterns studied (Fig. 2 and Supplementary Fig.S1). In line with Zhang et al.^22^, our findings suggest that a higher optical objective captures the characteristics of polish more effectively. However, this result should be interpreted with caution, as the smaller sample size for the 10× objective may have contributed to the observed lower performance. Further research using different objectives, microscopic image techniques, contact materials, and models is necessary to draw more secure conclusions. The finding that images capturing smaller areas of a tool’s surface are better suited for classifying polish may also be influenced by distortion introduced when resizing images to 224 × 224 pixels, which could favor one objective over another. Since this image size was selected to meet ResNet50’s requirements, a more detailed investigation of this effect was not possible, however, it remains an important avenue for future research.

The choice of an optimal window of analysis (patch size) is not straightforward, as it appears to depend on the model and the contact material. However, the analysis of smaller surface areas (division into 16 regions) most frequently yielded better and more consistent F1-scores, as shown in Fig. 2 and Supplementary Figs. 1, 2. Once again, our results align with Zhang’s^22^ findings that dividing the image into more patches leads to better performance, which, according to their interpretation, occurs because smaller patches maintain a resolution closer to the original image, preserving more detail and improving the model accuracy. A similar study was conducted by Sferrazza^13^, in which specific areas of images were cropped to 300 × 300 and 600 × 600 pixels, with larger patches yielding better results. However, we cannot directly compare our results or Zhang’s^22^ findings with those of Sferrazza’s approach, as the images were not systematically cropped into regions or resized for input into models, which could lead to a loss of resolution. Further research is needed to determine whether the analysis of smaller surface areas improves performance by better capturing the pattern of polish, or simply it is better suited to specific datasets or contact materials.

Considering the median and variation of the materials’ f1-scores, precision and recall, hide was the material most consistently classified correctly, followed by bone, whereas wood exhibited the lowest classification performance. Several factors could account for the model’s difficulty in classifying wood polish. One possible explanation is data imbalance, as the wood polish samples may have been underrepresented. However, this is unlikely, as the class imbalance was mitigated through weighted adjustments during training, assigning greater importance to the minority class (see Supplementary Table S3). Another potential factor is model bias, which can arise from inconsistencies in data processing and analysis. However, this is improbable, as preprocessing was standardized across all classes (see scripts in Code availability section). Given that methodological limitations have been addressed, the most plausible explanation for the misclassification of wood polish is its intrinsic characteristics, which may lack sufficient distinctiveness for a reliable classification.

A direct comparison between this study and previous research applying CNNs in polish images is challenging due to differences in models, hyperparameters, contact materials, raw materials, and experimental methodologies, all of which significantly influence classification performance. However, to contextualize these findings within the existing literature, the classification performance reported in key studies is hereby summarized. Pedergrana et al.^11^, who quantified polish on quartzite using Decision Trees (DT) and Support Vector Machines (SVM), reported 100% accuracy for bone and hide, 67% for wood, and 40% for cane and antler. While the raw materials and models used differ from those in this study, the overall trend of higher accuracy for bone and hide compared to wood aligns with our findings. Sferrazza^13^ reported classification accuracies of 85% for butchering traces and wood, 68% for shell, and 56% for antler. In a subsequent study, Sferrazza^27^ achieved 97% accuracy for plant traces, 84% for hide, 65% for shell, 63% for butchering, 42% for wood, 37% for bone, and 32% for antler. These variations underscore the impact of methodological choices on classification performance. The general trend of higher accuracy for certain contact materials, such as hide and bone, is consistent across most studies, suggesting that intrinsic material properties may play a significant role in their successful classification.

According to Vaughan^29^, use intensity correlates with the development of polish progressing through three distinct phases: generic weak, smooth pitted, and well-developed or diagnostic. This study aimed to classify polish based on stroke count using samples generated with 1000 and 2000 strokes. According to Ibáñez and Mazzucco^10^, their chosen stroke counts approximate 12,5–16,7 min (1000 strokes) and 25–33,3 min (2000 strokes) at 60–80 strokes per minute, aligning with their classification of polish development stages (10–20 min, 30–40 min, and 50–60 min). Owing to limited data availability, the analysis was restricted to wood polish, precluding the examination of other contact materials. The results demonstrate that it is feasible to classify the dataset based on stroke count with an accuracy of up to 93%, with the polish created using 1000 strokes exhibiting better classification performance than that created using 2000 strokes. This finding suggests that for the CNN models, the polish produced with 1,000 strokes differs markedly from that produced with 2,000 strokes. The higher error rate for the 2000-stroke polish could suggest that, at higher stroke counts, polish may lose distinguishing characteristics or exhibit greater variability. This aligns with prior research indicating that polish reaches a phase of stability characterized by increased homogeneity and overlapping classifications^10,17,30,31^. To investigate whether 2000 strokes represent a potential boundary or a transition toward a more homogeneous phase, further experiments are needed to assess the differentiation of polish, under controlled conditions, at progressively higher stroke counts.

Nevertheless, this study has several limitations stemming from both the data and nature of CNNs. First, the need to eliminate unfocused areas from the images may have led to a significant loss of information. Second, the dataset size was significantly smaller than that typically used for CNN models, which likely contributed to the instabilities observed during model training. Even though data augmentation, weighted training, and stratified dataset splitting were used to mitigate this issue, techniques such as data augmentation do not create new information; they only generate variations of the existing data. Third, in their current state, the models can only be successfully applied to datasets that are comparable in terms of raw material, activity, and acquisition settings, as the dataset used had only a certain level of variability. Fourth, the pre-trained CNNs show lower performance as they are trained on datasets with patterns that are significantly different and simpler compared to those in the use-wear data.

Advancing the application of CNNs in use-wear analysis requires further research by both use-wear analysts and computational archaeologists. To develop robust and generalizable models, larger and more diverse datasets are essential^32^. Expanding datasets with additional raw materials (e.g., different types of flint, quartzite, and obsidian), contact materials (e.g., antler, shell, cane, and various types of wood and bone), and acquisition settings (e.g., different objectives and microscopes) will enhance model stability by providing complementary information to existing models, improving classification confidence. Furthermore, exposing CNNs to a broader range of data is essential for more accurately capturing the complexity inherent in the archaeological record^11^. Further research is also needed to optimize CNN architectures, refine preprocessing techniques, incorporate robust evaluation strategies such as cross-validation, and develop models that can better handle the needs of use-wear patterns. Given the inherent challenges of polish patterns, characterized by their complexity, lack of clear boundaries, variability, and frequent overlap, archaeologists need to adapt existing tools or develop new methodologies tailored to these data^22^. Optimizing CNN architectures for use-wear analysis is a trial-and-error process requiring extensive experimentation, highlighting the importance of further research and the exchange of data and methodologies within the research community. Adopting open science practices will facilitate and accelerate this process by fostering collaboration, enhancing transparency, and ensuring that research adheres to FAIR principles (Findable, Accessible, Interoperable, and Reusable)^33,34^. Open access to datasets, models, and methodologies will not only improve reproducibility, but also support the collective effort required to establish CNNs as a reliable tool in use-wear analysis.

In summary, this study demonstrates that custom CNN architectures can offer a promising approach for classifying experimental use-wear polish on lithic artifacts. However, the lower performance observed for wood polish and the higher error rate for images acquired with a 10× objective and 9-patch size highlight the complexity of the dataset and the need for further research. Future work should focus on expanding the dataset in terms of size and variability, optimizing CNN architectures, and refining preprocessing techniques. Additionally, promoting collaborative research and adopting open science practices, including data sharing, transparent methodology, and reproducible research, are critical for improving the robustness and reliability of deep learning techniques in use-wear analysis.

## Methods

The classification of use-wear images based on contact material and the number of strokes revolves around two research axes: the effect of the surface area analyzed, as determined by patch size and objective, and the suitability of a custom-trained or pre-trained model for this task. With the exception of image acquisition, all processes involved in conducting the analyses and synthesizing this study are openly shared in a Zenodo repository (see Data availability). Python scripts were used for data preprocessing (including cleaning, feature extraction, and standardization) and analysis (model development and evaluation), and a Quarto file was created to visualize the results and draft the current manuscript. The reproducibility of our analysis was further ensured by providing a text file listing the names and versions of the libraries used along with the Python version. To ensure consistent results across different runs, a random seed (= 42) was set for both Python and TensorFlow operations. A preprocessed and cropped subset of the dataset is provided in the repository to facilitate the reproducibility of the current analysis, while the full dataset will be provided in a separate publication.

### Data acquisition and organization

The dataset consists of 69 grey-scale images of polish created using controlled experimental protocols from 43 flint tools. Each tool was knapped by hand using a hard hammer and used with unidirectional movements (one stroke per movement) for scraping, and bidirectional movements (two strokes per movement) for sawing, depending on the working material. Each tool was used with a single motion on a single material for a specified number of strokes (1000 or 2000). Three different working materials were processed during this experiment: wood (*Pinus pinaster*), bone (*Ovis aries*), and hide (*Ovis aries*). The number of variables selected (i.e., contact materials and strokes) was intentionally limited to isolate their individual effects on polish classification, to focus on use-wear types that analysts can reliably distinguish visually (see Vaughan^29^on polish type interpretability) and to ensure consistency with parameters established in previous studies (e.g., Pedergnana et al.^11^ for contact materials; Ibanez and Mzzucco^10^ for stroke counts). The resulting polishes were documented using a reflected light microscope (ZEISS Axio Scope.A1 MAT, objective EC Epiplan 10x/0.25 M27, Objective EC Epiplan 20x/0.4 M27). All images were acquired using the ZEISS Zen Core software and the Extended Depth of Focus (EDF) stacking module. The image frame size is 2464 × 2056 pixels, and they were saved in TIFF format. Images acquired at 10x magnification have a numerical aperture (NA) of 0.25, a field of view (FOV) of 850.08 × 709.32 μm, a depth of field of 8.800 μm, and a pixel size of 0.345 μm. Images acquired at 20x magnification have an NA of 0.40, an FOV of 425.04 × 354.66 μm, a depth of field of 3.438 μm, and a pixel size of 0.173 μm. Detailed information on the data distribution by material, objective, and stroke count is presented in Supplementary Table S4 and a summary of experimental design and acquisition settings in Supplementary Table S5. The filenames of the images were standardized to encode the contact material, activity, number of strokes, area, subarea (a single tool may have multiple areas of polish), and objective. Duplicate images were removed during the process.

### Data preprocessing

#### Data cleaning

Due to the inherent limitations of the microscopy equipment and the position of the polish relative to the shape of the tools, the images included areas that were (a) out of focus and (b) beyond the surface of the tool. The former results from the microscope’s limited depth-of-field^24^, and the latter occurs because the polish is primarily located at the edge of the tool, causing some parts of the image to show the background beyond the tool surface. To isolate the in-focus regions, the Sobel operator (cv2.Sobel) was applied to detect changes in pixel intensity (edges) as a proxy for texture^35,36^. In-focus areas exhibit sharp intensity changes (strong edges), whereas out-of-focus or background areas exhibit smoother transitions (weak or no edges)^24^. Sobel’s output was then processed using Otsu’s method (cv2.THRESH_OTSU) to determine the optimal threshold between the in-focus and out-of-focus areas^35^. Morphological operations (cv2.MORPH_CLOSE, cv2.MORPH_OPEN) and watershed segmentation (cv2.watershed) were applied to remove noise and refine the final mask used to exclude unwanted areas (ibid.) (see Supplementary Fig. S3). The resulting masks and excluded areas were visually inspected by use-wear specialists to ensure accuracy, thereby incorporating expert knowledge into the workflow. For the majority of images, between 30% and 60% of the surface area was cropped (i.e., removed regions), while images with more than 80% cropped area were excluded from further analysis.

#### Image enhancement and feature extraction

Data preprocessing was performed to mitigate artifacts introduced during image acquisition, such as blurring from light diffraction and reduced contrast in in-focus objects due to out-of-focus light, and to enhance key features, specifically the polished areas^24^. Contrast-limited adaptive histogram equalization (CLAHE) was applied to improve and standardize the image contrast. Since pixel values are constrained within a specific range, CLAHE segments the image into small tiles and redistributes their intensity values more uniformly^24^. To minimize outliers, extreme intensity values were clipped and reassigned before applying histogram equalization^37^.

#### Image division and standardization

To investigate the effect of patch size on CNN performance, the images were divided into equally sized, non-overlapping patches (resampled) using either a 4 × 4 grid (resulting in 16 patches, 514 × 616 pixels) or a 3 × 3 grid (resulting in nine patches, 685 × 821 pixels). The pixel values of each patch were normalized to the range of 0 to 1 and resized to 224 × 224 pixels to ensure homogeneity and to meet the input size requirements of ResNet50^38^. The subsampling of the dataset increased the number of images available for analysis, with the 3 × 3 grid yielding 237 images for bone, 155 for wood, and 67 for hide, and the 4 × 4 grid yielding 337 images for bone, 273 for wood, and 117 for hide. Despite this improvement, we acknowledge that the dataset remains relatively small, particularly considering that convolutional neural networks (CNNs) typically require large amounts of data to train effectively^24^, however, this limitation is addressed through several considerations. First, the dataset was created independently of the present study and made available only after its collection, which precluded any control over its initial design or class distribution. Future work will aim to integrate data collection and computational design from the outset to better align with ML requirements. Second, the dataset was acquired using a standardized and consistent protocol (see Data Acquisition and Organization), with all experimental and imaging procedures conducted by a single individual. Although this approach naturally restricted the volume of data, it minimized technical and operator-related variability, thereby improving the internal consistency and quality of the dataset. Finally, it is important to emphasize that this study is exploratory in scope. Rather than presenting a fully developed classification tool, the objective was to assess the feasibility of applying CNNs to use-wear classification using a carefully controlled, albeit limited, dataset. In this context, working with a limited but high-quality dataset was considered appropriate for an initial evaluation of the method’s potential and to inform future research using larger and more diverse data.

#### Dataset splitting

The dataset was split into train (70%), test (15%), and validation (15%) sets using the train_test_split function from sklearn^38^ with stratification to preserve class distribution across all subsets. The training set (X_train, y_train) was used for data augmentation and model training, the validation set (X_val, y_val) was used to tune the hyperparameters and evaluate the model during training, and the test set (X_test, y_test) was used to evaluate the final performance of the model after training.

#### Handling class imbalance

Due to time constraints, the collection of additional data was not possible. However, several measures were taken to mitigate the effects of both the small size and unbalanced nature of the dataset on the robustness of CNN models. During the splitting of the dataset, the ‘stratify’ parameter was set to ensure that the distribution of classes was preserved in all sets, avoiding bias due to uneven class representation. The sklearn.utils.class_weight function was also used to assign higher weights to the minority class during training to balance the effect of class underrepresentation^38^.

#### Data augmentation

Data augmentation was used to artificially increase the size of the training dataset by creating modified versions of existing images. This was achieved using the ImageDataGenerator class from TensorFlow’s Keras API with augmentations including 30% zoom, horizontal flip, vertical flip, and 30% width and height shifts. These transformations were deliberately chosen to introduce realistic variations in image scale and orientation while minimizing distortions that could produce non-representative patterns. Preliminary tests with more aggressive and diverse augmentations (e.g., brightness adjustments or larger shift percentages) showed that such transformations introduced visually unrealistic alterations, which corresponded to decreased model performance. Depending on the model, five to seven augmented versions were generated for each of the original images. Supplementary Table S2 displays the dataset split sizes for each model before and after the data augmentation.

### Model architecture and development

The dataset was divided into seven unique subsets based on combinations of microscope objectives, stroke counts, and patch sizes. However, due to the specific parameter combinations (Supplementary Table S4), it was not feasible to include all three classes (bone, hide, wood) in a single model. As a result, binary classification was applied for each subset: bone vs. wood, bone vs. hide, and hide vs. wood. For use intensity, the models were trained exclusively on wood polish images, comparing 1000 strokes against 2000 strokes. Both custom and pre-trained CNNs were applied to these subsets, resulting in a total of 14 models.

Using a single custom and pre-trained CNN for each set was impractical because the varying preprocessing and acquisition parameters effectively created distinct datasets, each with unique characteristics that required different models.

**Fig. 8:**
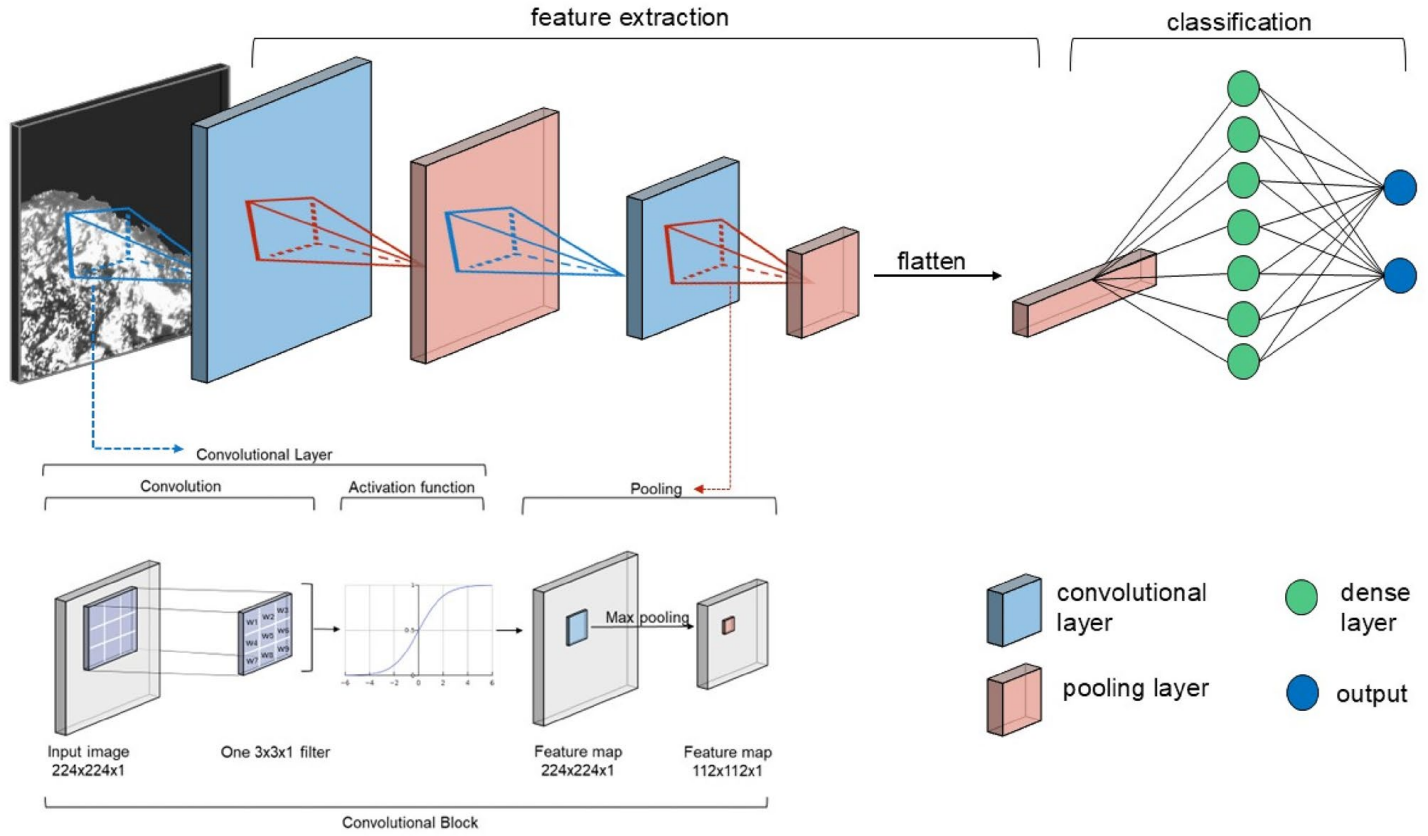
Schematic representation of a CNN architecture. The network is composed of multiple convolutional layers used for feature extraction, each followed by an activation function and pooling layer. The extracted features are then flattened and passed through fully connected (dense) layers for classification.

A typical CNN is composed of three types of layers: a convolutional layer, pooling layer, and fully connected layer (Fig. 8)^24^.

The convolutional layers slide a grid (called a kernel or filter) of numbers (called weights) over an image and perform element-wise multiplication with the corresponding region in an image^25^. The results are summed, and a given value (called bias) is added and then passed through an activation function to produce a single value at each position, thus generating a feature map that highlights different features^39^. Both weights and bias are parameters that are adjusted during training to minimize the error of the model^40^. The activation function enables the model to capture complex patterns and determine which features are most important^36^. Convolutional layers located earlier in the model typically identify simpler elements, such as edges and textures (low-level features), whereas later layers identify more complicated patterns, such as faces and objects (high-level features)^25^. In this study, low-level features correspond to elements such as texture and brightness, while high-level features capture the spatial distribution and intensity patterns among these elements that define the polish.

The pooling layer reduces the spatial dimensions of the feature maps by downsampling (making an image smaller by deleting pixels)^24^. Pooling is performed by sliding a window over the feature map, and at each location, either computing the average pixel value (average pooling) or selecting the maximum pixel value (max pooling)^36^. The resulting values form a lower-resolution version of the original feature map, while preserving important spatial information and reducing computational costs^25^. The fully connected (or dense) layer is a simple artificial neural network (ANN) where every input unit *(i.e.*,* pooling output)* is connected to every output unit (i.e., classification result)^24,25,36^. As a comprehensive theoretical discussion of convolutional neural networks (CNNs) is beyond the scope of this study, further details can be found in^24,25,36,39^.

#### Custom CNN architecture

The model architectures were developed based on empirical performance criteria. Due to computational constraints, fully systematic hyperparameter optimization was not feasible. Instead, the architecture design was guided by manual evaluation of multiple performance indicators, including accuracy relative to chance level (e.g., 50/50 classification), the shape of learning curves to identify under- or overfitting, and confusion matrices to assess class-specific performance. This approach allowed for a more nuanced interpretation than reliance on a single evaluation metric. Based on these criteria, the architecture of the seven models was adapted to the specific characteristics of each dataset, while maintaining a largely consistent overall structure (see Fig. 8). Most models incorporate three convolutional layers with the number of filters increasing in various combinations of 8, 16, 32, 64, and 128. Each convolutional layer employed 3 × 3 kernels, ReLU activations, and MaxPooling2D for downsampling. L2 regularization was applied across all models, and approximately half of them included dropout to mitigate overfitting. Following feature extraction, a Global Average Pooling (GAP) layer was used to reduce the spatial dimensions of the feature maps. The number of fully connected layers varied, ranging from two to four dense layers, with a progressively decreasing number of neurons in combinations of 128, 64, 32, and 16. All dense layers incorporate L2 regularization and batch normalization to enhance the model stability and generalization. ReLU activation was consistently applied, and the majority of models integrated dropout to prevent overfitting.

#### Pre-trained CNN architecture

All pre-trained models were built on the ResNet50 architecture, which was pre-trained on the ImageNet dataset (3 channel red-green-blue (RGB) images, 224 × 224 pixels)^40^. To align our single-channel image dataset with ResNet50’s expected input shape, we replicated the grayscale channel three times to mimic the shape of an RGB image. We acknowledge that replicating the same information across three channels introduces redundancies and increases the computational cost^32^; however, this approach is a common practice in other fields facing similar challenges and serves as a necessary adaptation to meet the requirements of ResNet50 (e.g^41–43^.

ResNet50 consists of 49 convolutional layers with the number of filters increasing progressively from 64 to 256, 512, 1024, and 2048 using 3 × 3 and 1 × 1 kernels, ReLU activations, batch normalization, and skip connections to enable deep network training^40^. In this model, all layers retained their pre-trained weights as part of the transfer learning process, while the final 30 layers were set to update their weights during training (a process known as unfreezing), enabling the model to fine-tune and adapt to the features specific of our dataset. This configuration was determined through iterative experimentation, as alternative setups involving the unfreezing of either more or fewer layers consistently yielded lower classification performance across all datasets (see Supplementary Table S6 for detailed performance metrics and learning curves). After feature extraction, a Global Average Pooling layer is used to reduce the spatial dimensions of the feature maps, and a dropout layer with a rate of 0.5 is added to prevent overfitting. Finally, a dense layer consisting of a single neuron with a sigmoid activation function was responsible for binary classification.

#### Optimization techniques

The goal of training is to minimize the error of the model, defined as the difference between its output and ground truth, quantified by a loss function^24^. The role of an optimizer in this process is to adjust the weights of the model after each training iteration such that the error reaches its lowest possible value (referred to as the global cost minimum)^36^. Given that the dataset for each model consists of only two classes (e.g., wood and bone, hide and bone, 1000 strokes and 2000 strokes), the binary cross-entropy loss function was selected for both the pre-trained and custom CNNs. The Adam optimizer was employed for all models^44^.

The learning rate is a user-defined parameter (aka hyperparameter) that controls the magnitude of the adjustments made by the optimizer to the model’s weights to reach the global cost minimum^25^. As model training is a dynamic process, using a fixed learning rate may not be optimal^25^. Therefore, a learning rate reduction algorithm (ReduceLROnPlateau) was used to monitor the validation loss and adaptively decrease the learning rate if no improvement was observed^45^. Similarly, to stop training at the point of minimal validation error, early stopping was applied (EarlyStopping)^45^. This procedure helps prevent overfitting, a phenomenon in which the model memorizes the training data instead of effectively generalizing unseen data, which is typically indicated by an increase in validation loss and a simultaneous decrease in training loss^39^.

#### Model evaluation

The CNN models were evaluated using various metrics to assess both their performance (how well the model makes predictions) and interpretability (how the model creates its predictions). The evaluation metrics used were accuracy, loss, precision, recall and the f1-score. These metrics were recorded as reports and visualized using graphs, including confusion matrices, learning curves, and ROC curves. To gain a deeper insight into the model’s performance, the filenames of misclassified images (e.g., those with a true label of ‘bone’ but classified as ‘wood’) were recorded to identify potential patterns associated with specific parameters, such as whether all misclassifications occurred for a certain model or contact material.

Neural networks are frequently referred to as “black boxes” as they provide limited or no insight into why and how specific features are important for the model’s decisions^46^. Given the importance of not only obtaining the correct predictions but also providing the right explanations for how those predictions are made, researchers have developed tools to enhance model interpretability, defined as ‘the ability to understand and reason about a model’s output‘^47,48^.

Interpretability methods can be categorized into different taxonomic frameworks, depending on the specific aspects used for classification^49^. These frameworks can be grouped into four approaches: the functioning-based approach (focusing on how a method extracts information from a model), result-based approach (categorizing methods based on their outcome), conceptual approach (using distinct conceptual dimensions, such as scale and time of implementation), and mixed approach (a combination of the above)^50^. For this study, we followed the taxonomy by Zhang et al.^51^ because of its ability to address multiple dimensions. Zhang’s taxonomy combines a result-based and conceptual approach and consists of three dimensions: (1) local or global, referring to whether the method explains individual prediction(s) or the overall decision-making process of the model (2) passive or active, depending on whether the method is applied after training or modifies parts of the model before training; (3) type of explanation, based on the method’s output.

The interpretability methods used in this study were Score-CAM, Integrated Gradients, and SmoothGrad. All three fall under the category of “local passive attribution” methods, and their main goal is to create heatmaps that highlight which input features (e.g., pixels in an image) are most important for the model’s predictions. Score-weighted visual explanations for convolutional neural networks (Score-CAM) modify the input image using activation maps and then pass it through the network^52^. The change in the predicted class probability was used to calculate the importance scores, which were combined to generate a heatmap highlighting the most influential regions^52^. Integrated Gradients compare the original input (e.g., an image) to a baseline (e.g., a gray image), compute gradients (how the model’s output changes) at multiple steps as the input transitions from the baseline to the original, and aggregate them into a heatmap^53^. SmoothGrad adds noise to the input image multiple times, computes gradients for each noisy image, and averages them to create a heatmap^54^.

## Supplementary Information

Below is the link to the electronic supplementary material.

Supplementary Material 1

## Data Availability

All materials associated with this paper are openly available in a Zenodo repository (https://zenodo.org/records/15106760?preview=1&token=eyJhbGciOiJIUzUxMiIsImlhdCI6MTc0MzMzNzYwNSwiZXhwIjoxNzY0NTQ3MTk5fQ.eyJpZCI6IjNmNzZkZmM1LTc2NDMtNDc4Yy1iNmQ3LTllZjNkMzk2NWYyYSIsImRhdGEiOnt9LCJyYW5kb20iOiJiYzg0MDVkN2VhZjI1NzgzZGQzM2U4YjBlMjIyOGFmOCJ9.WermUrWs5dIdw-6gHycikbXQPalfPYPmICX-9EDyQgh2jYpj0JVCy5kf0cyi3EbgUR6-cRaIvqk9Qy--K6oUTA). The following files are available: (a) Python scripts, including links to the GitHub repository containing all models and a CodeOcean capsule with a sample model and its environment (Dockerfile). (b) Quarto file used for manuscript and figure generation. (c) Supplementary information. (d) A preprocessed and cropped subset of the dataset to facilitate the reproducibility of this analysis. The full dataset will be provided in a separate publication.
