# Supplementary figures and images for: Classifying polish in use-wear analysis with convolutional neural networks (part 1 of 2)
Source: Sci Rep. 2025 Oct 22;15:36834. doi: 10.1038/s41598-025-18179-4 (PMC12546859; doi:10.1038/s41598-025-18179-4)

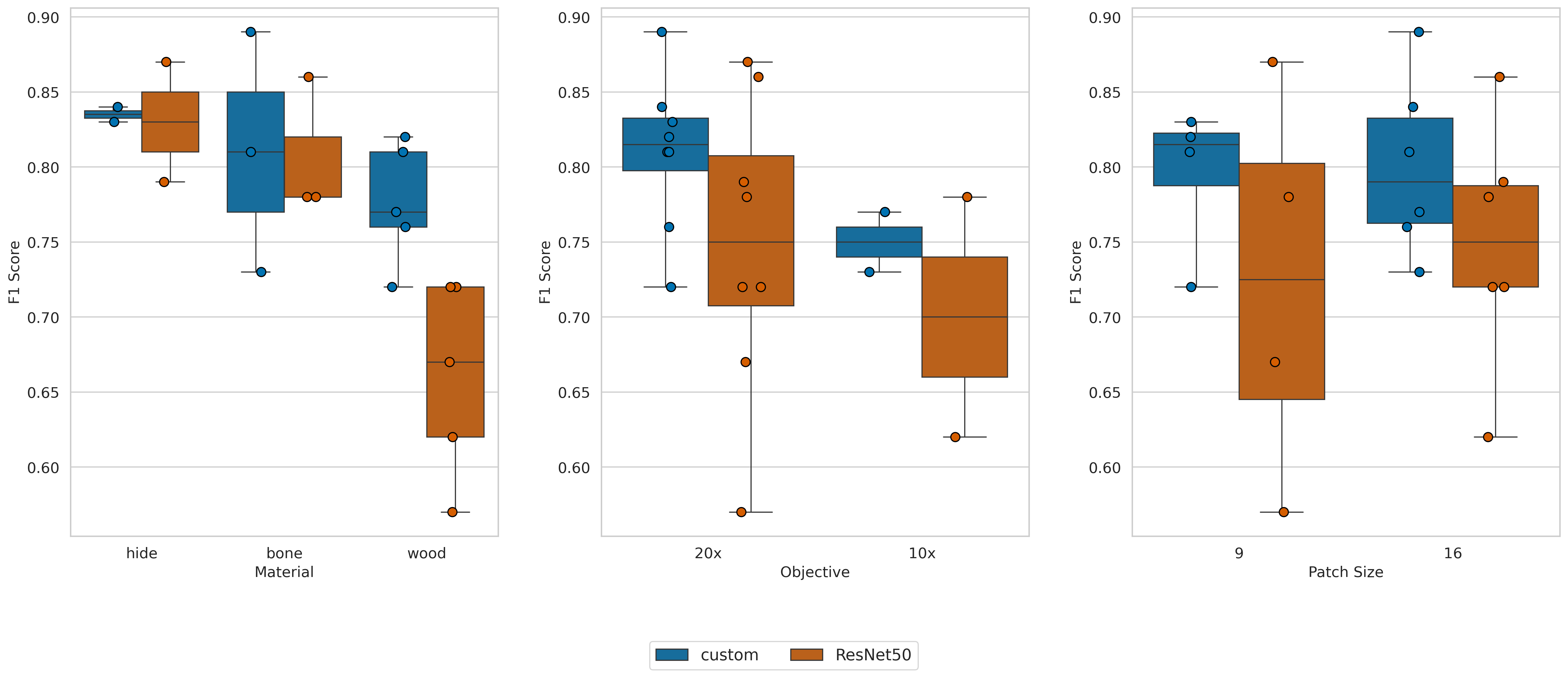

Supplement: Supplementary file 1 — Supplementary Material 1 [file 41598_2025_18179_MOESM1_ESM.tar › supplementary_material_resubmit1/Supplementary Figure S1.png]

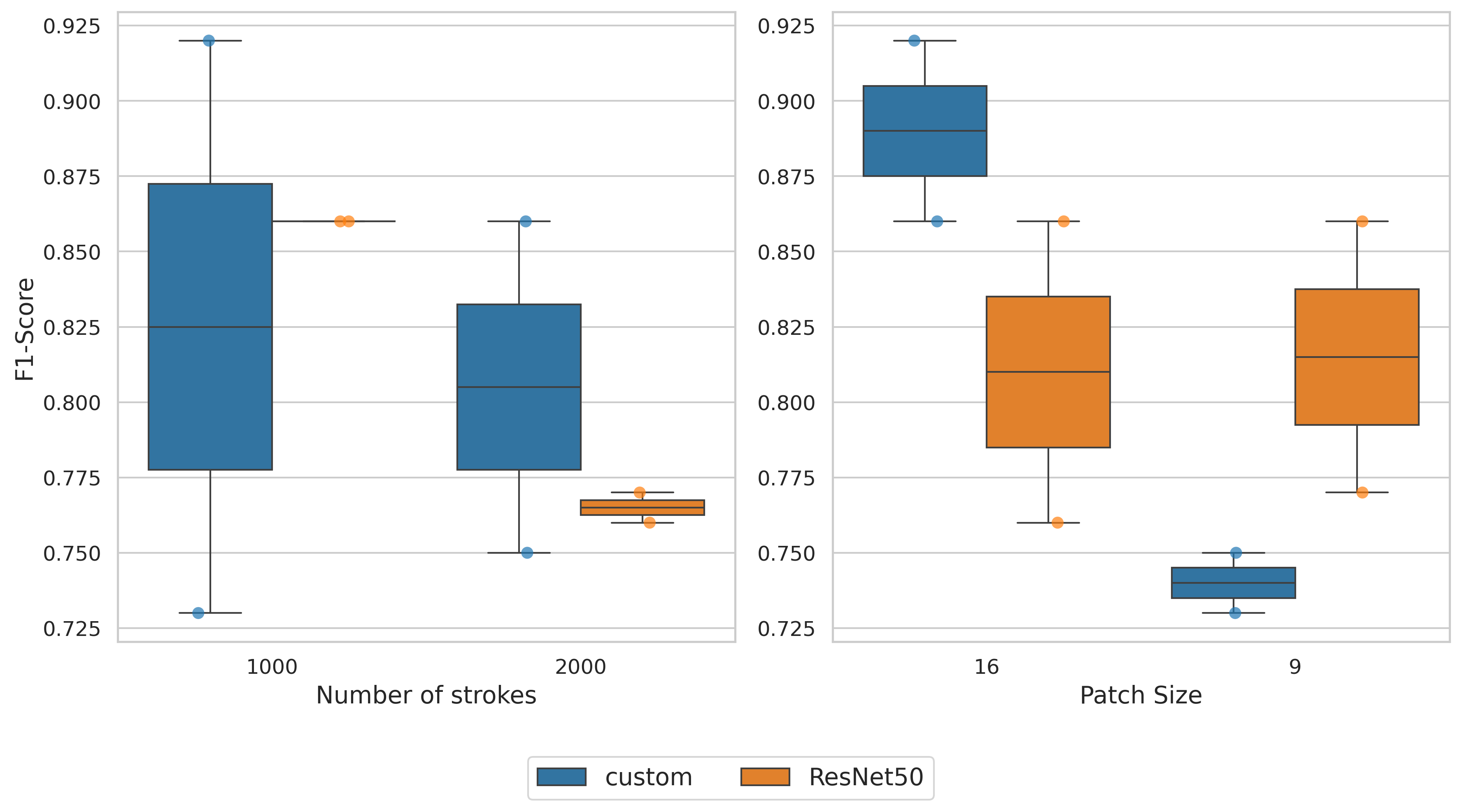

Supplement: Supplementary file 1 — Supplementary Material 1 [file 41598_2025_18179_MOESM1_ESM.tar › supplementary_material_resubmit1/Supplementary Figure S2.png]

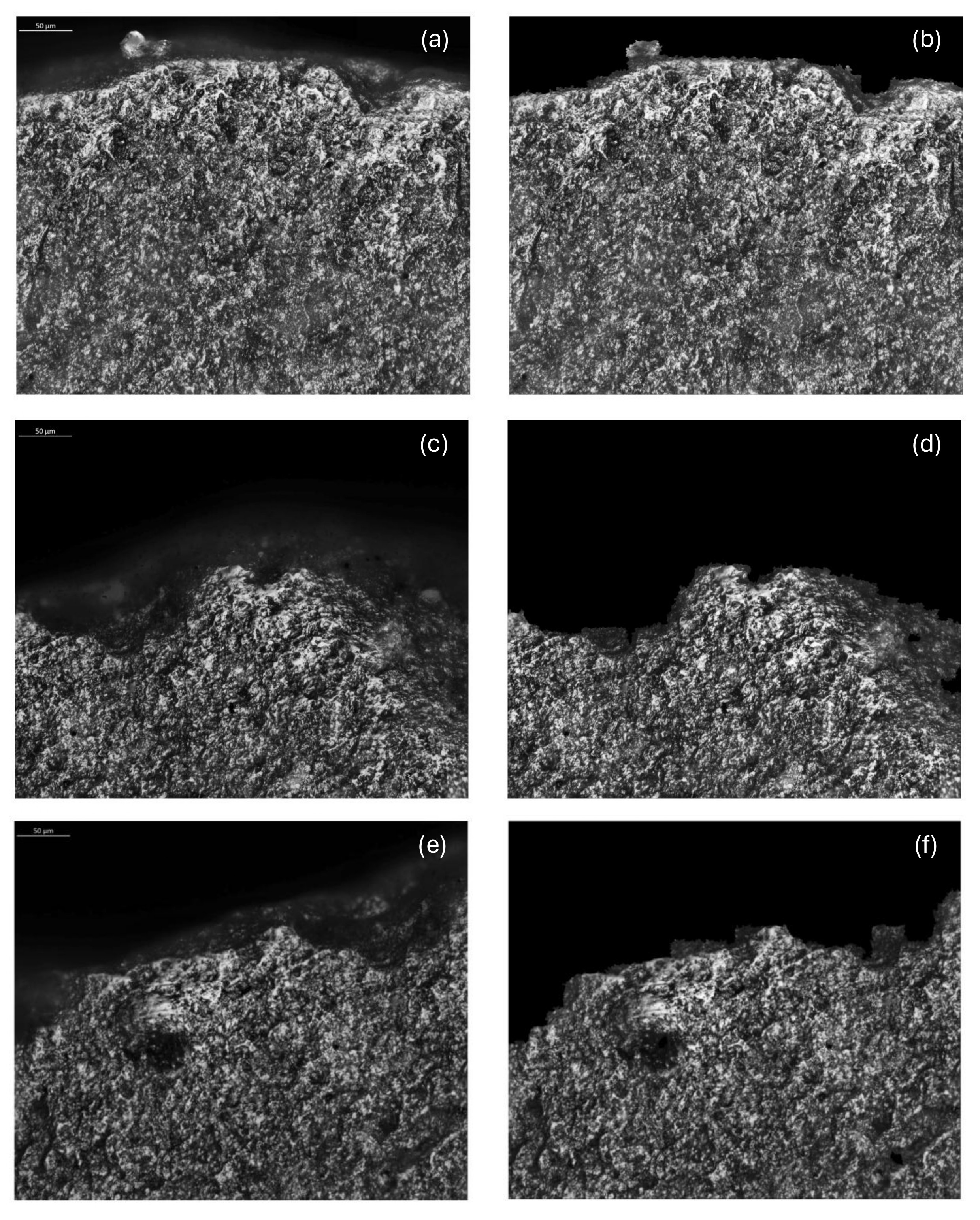

Supplement: Supplementary file 1 — Supplementary Material 1 [file 41598_2025_18179_MOESM1_ESM.tar › supplementary_material_resubmit1/Supplementary Figure S3.jpg]

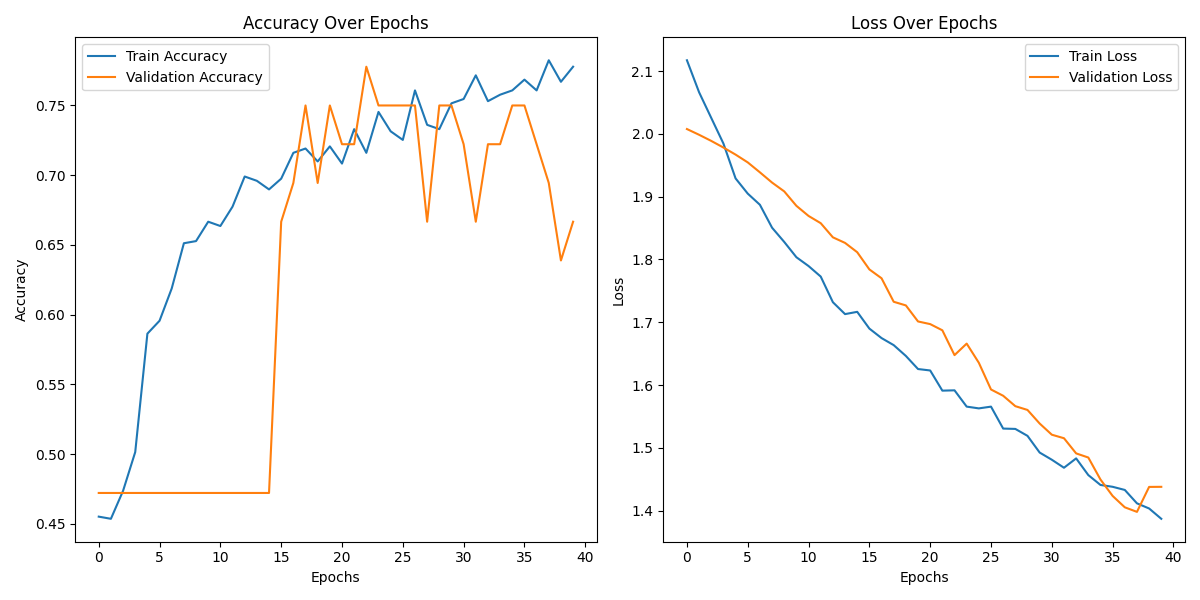

Supplement: Supplementary file 1 — Supplementary Material 1 [file 41598_2025_18179_MOESM1_ESM.tar › supplementary_material_resubmit1/Supplementary Figure S4/evaluation_metrics/custom/lc_10x_1000_16.png]

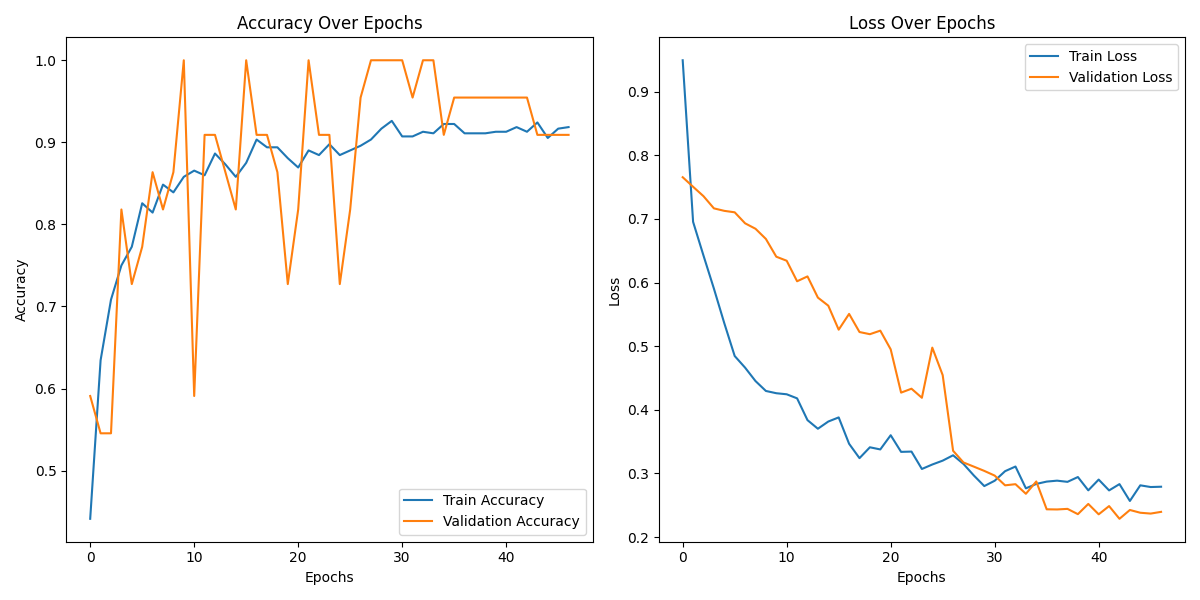

Supplement: Supplementary file 1 — Supplementary Material 1 [file 41598_2025_18179_MOESM1_ESM.tar › supplementary_material_resubmit1/Supplementary Figure S4/evaluation_metrics/custom/lc_10x_2000_9.png]

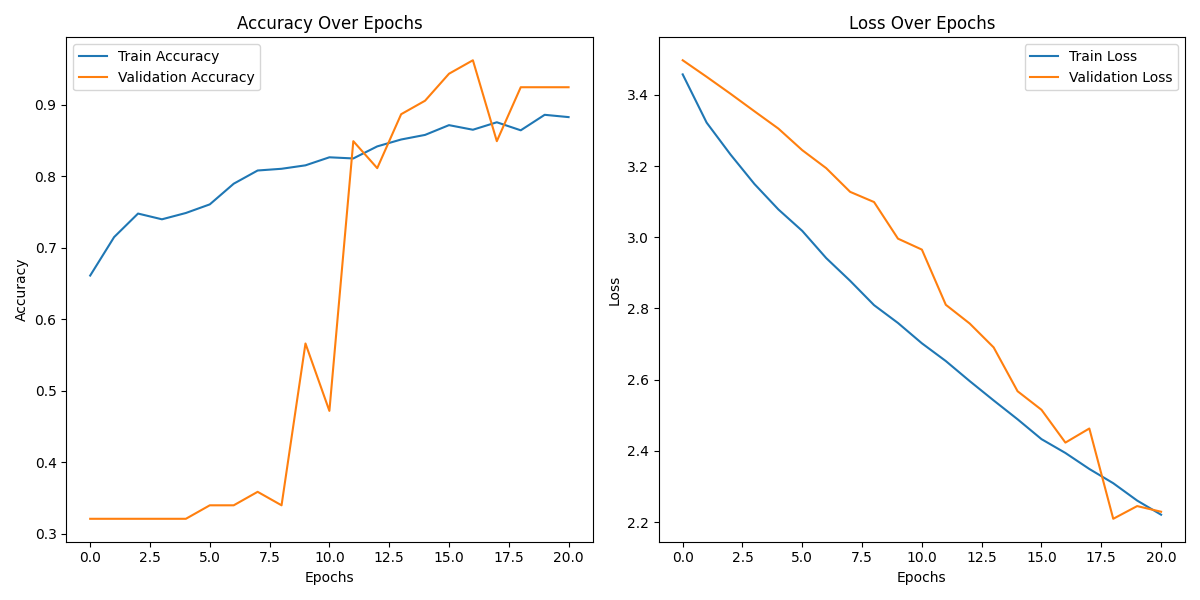

Supplement: Supplementary file 1 — Supplementary Material 1 [file 41598_2025_18179_MOESM1_ESM.tar › supplementary_material_resubmit1/Supplementary Figure S4/evaluation_metrics/custom/lc_20x_1000_16.png]

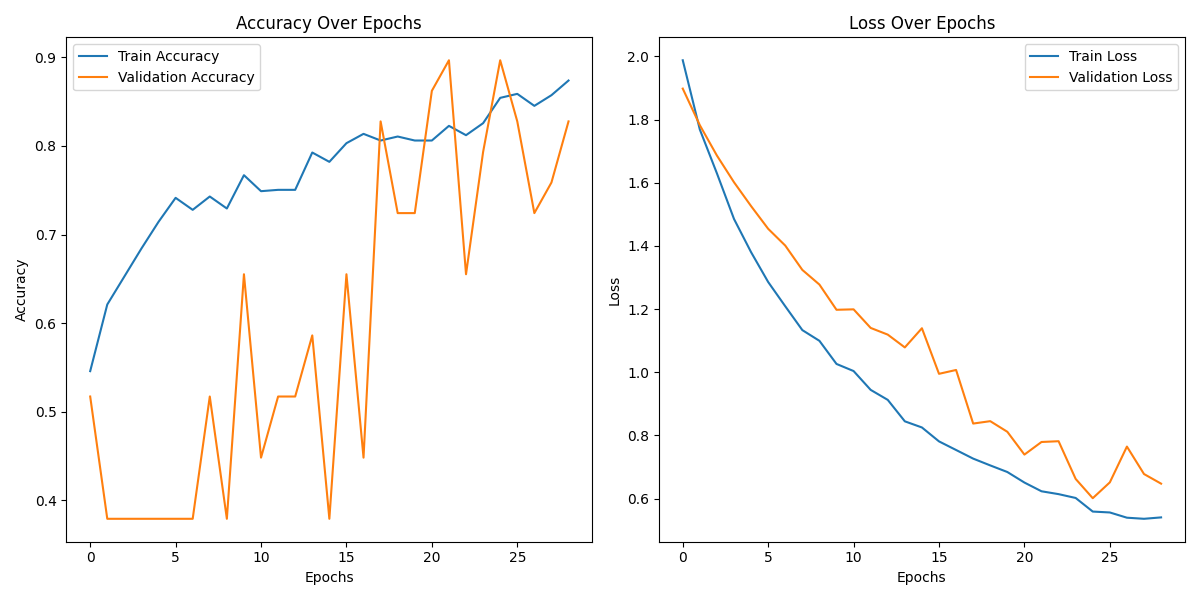

Supplement: Supplementary file 1 — Supplementary Material 1 [file 41598_2025_18179_MOESM1_ESM.tar › supplementary_material_resubmit1/Supplementary Figure S4/evaluation_metrics/custom/lc_20x_1000_2000_16.png]

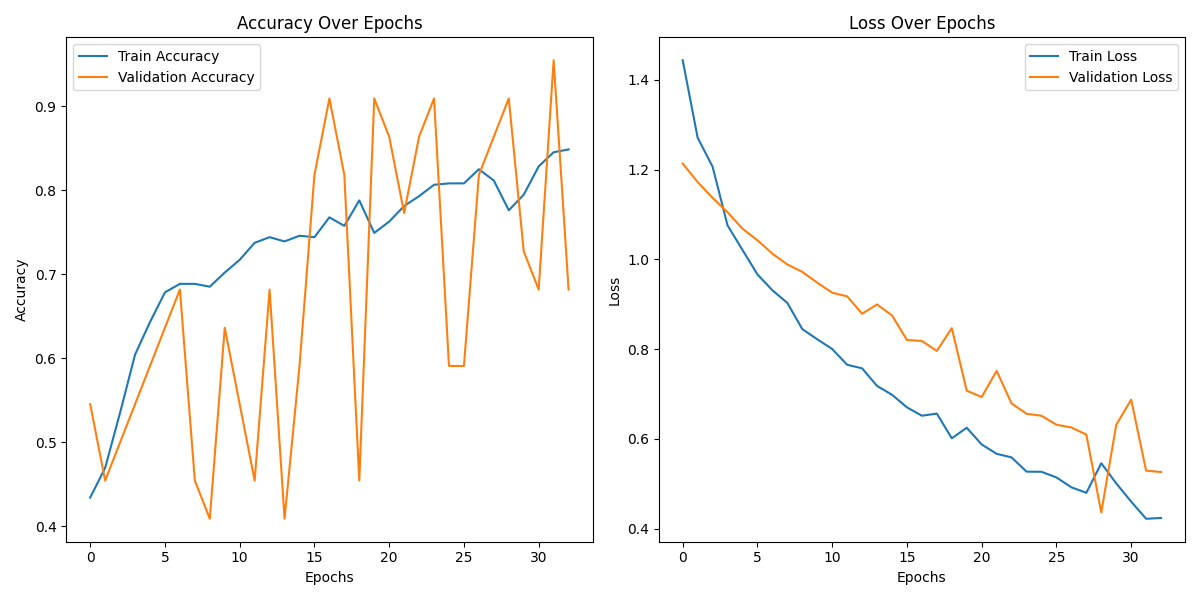

Supplement: Supplementary file 1 — Supplementary Material 1 [file 41598_2025_18179_MOESM1_ESM.tar › supplementary_material_resubmit1/Supplementary Figure S4/evaluation_metrics/custom/lc_20x_1000_2000_9.png]

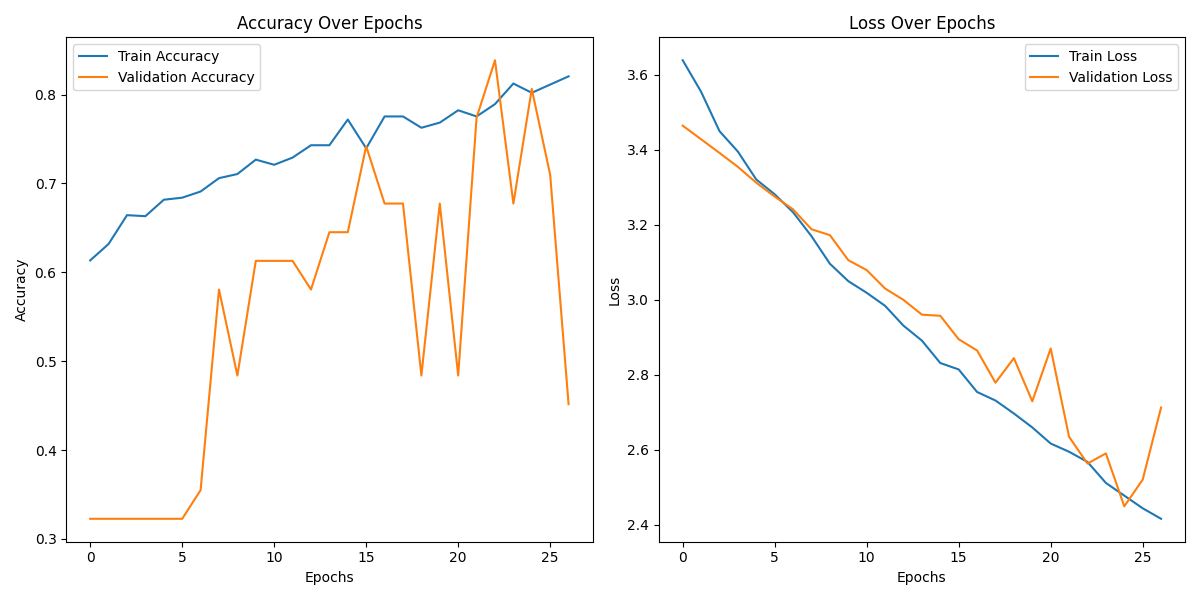

Supplement: Supplementary file 1 — Supplementary Material 1 [file 41598_2025_18179_MOESM1_ESM.tar › supplementary_material_resubmit1/Supplementary Figure S4/evaluation_metrics/custom/lc_20x_1000_9.png]

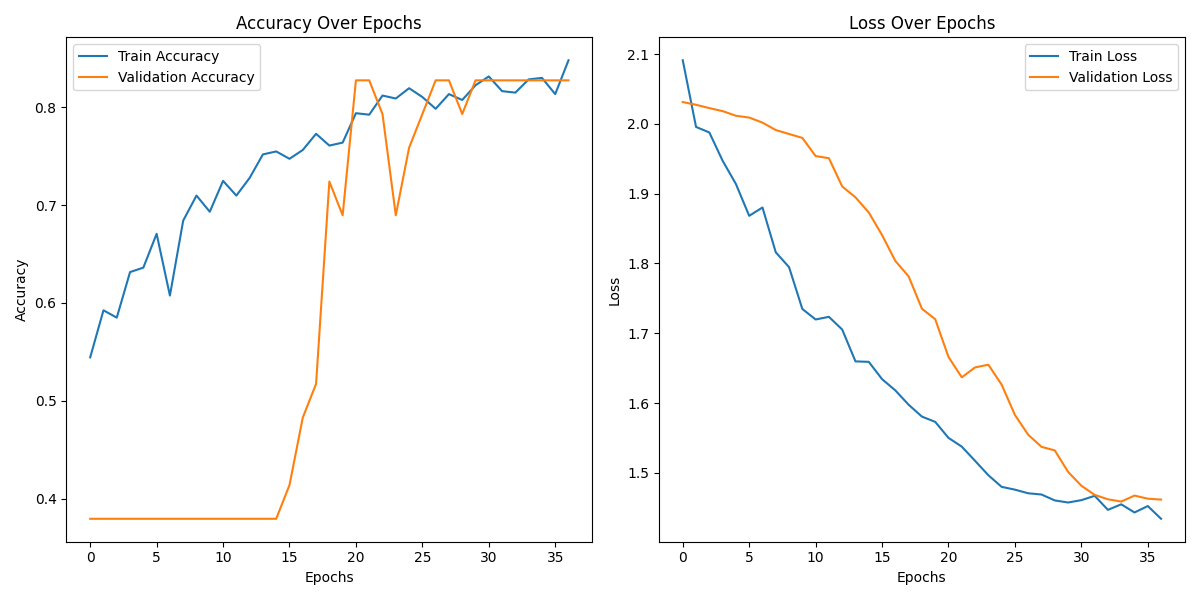

Supplement: Supplementary file 1 — Supplementary Material 1 [file 41598_2025_18179_MOESM1_ESM.tar › supplementary_material_resubmit1/Supplementary Figure S4/evaluation_metrics/custom/lc_20x_2000_16.png]

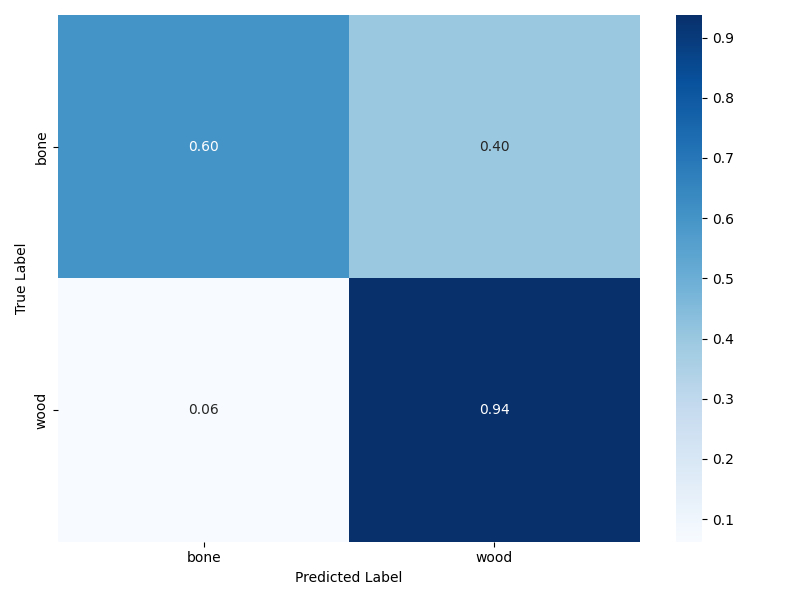

Supplement: Supplementary file 1 — Supplementary Material 1 [file 41598_2025_18179_MOESM1_ESM.tar › supplementary_material_resubmit1/Supplementary Figure S4/evaluation_metrics/custom/matrix_10x_1000_16.png]

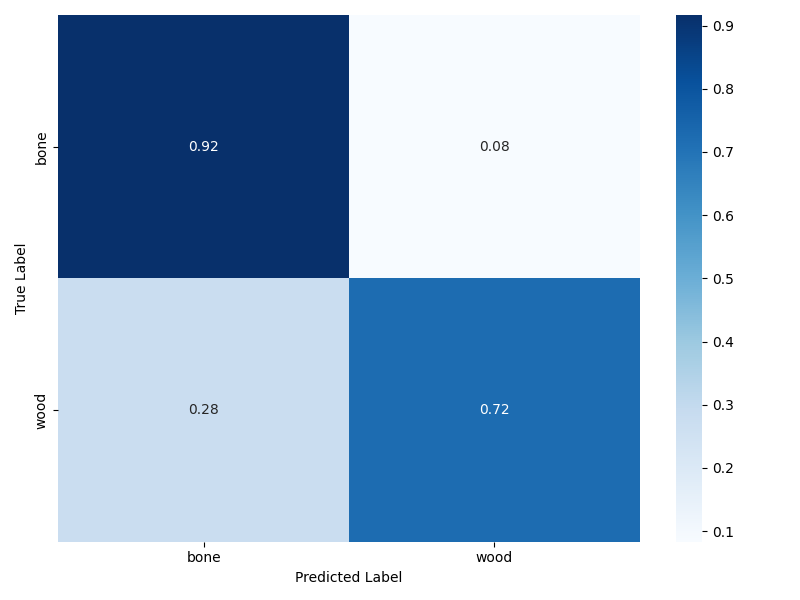

Supplement: Supplementary file 1 — Supplementary Material 1 [file 41598_2025_18179_MOESM1_ESM.tar › supplementary_material_resubmit1/Supplementary Figure S4/evaluation_metrics/custom/matrix_20x_1000_16.png]

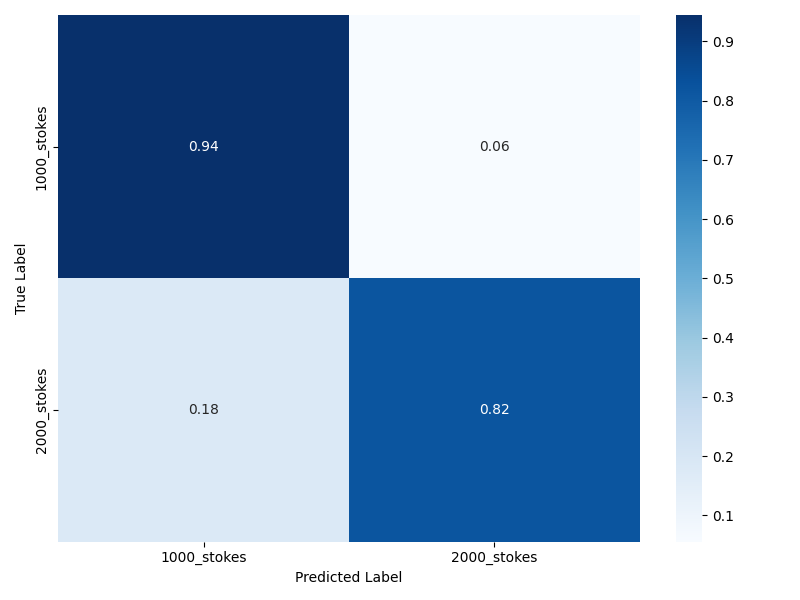

Supplement: Supplementary file 1 — Supplementary Material 1 [file 41598_2025_18179_MOESM1_ESM.tar › supplementary_material_resubmit1/Supplementary Figure S4/evaluation_metrics/custom/matrix_20x_1000_2000_16.png]

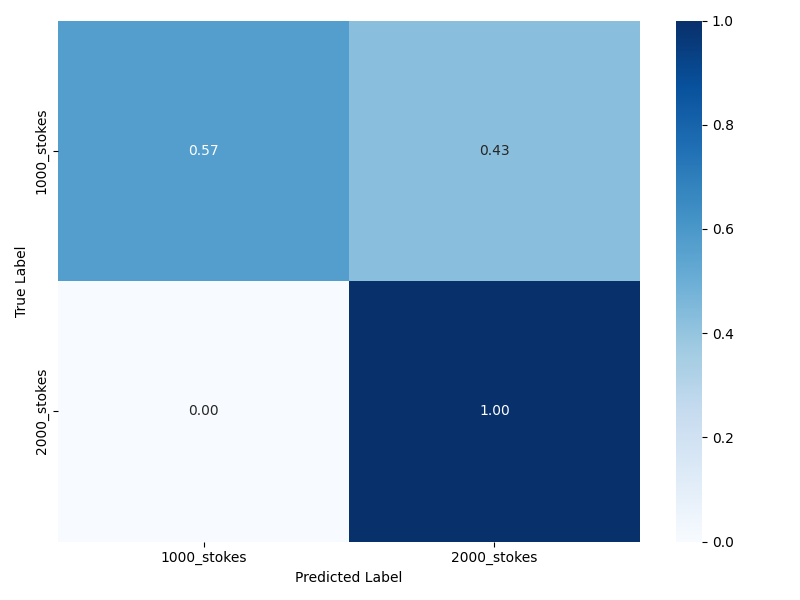

Supplement: Supplementary file 1 — Supplementary Material 1 [file 41598_2025_18179_MOESM1_ESM.tar › supplementary_material_resubmit1/Supplementary Figure S4/evaluation_metrics/custom/matrix_20x_1000_2000_9.png]

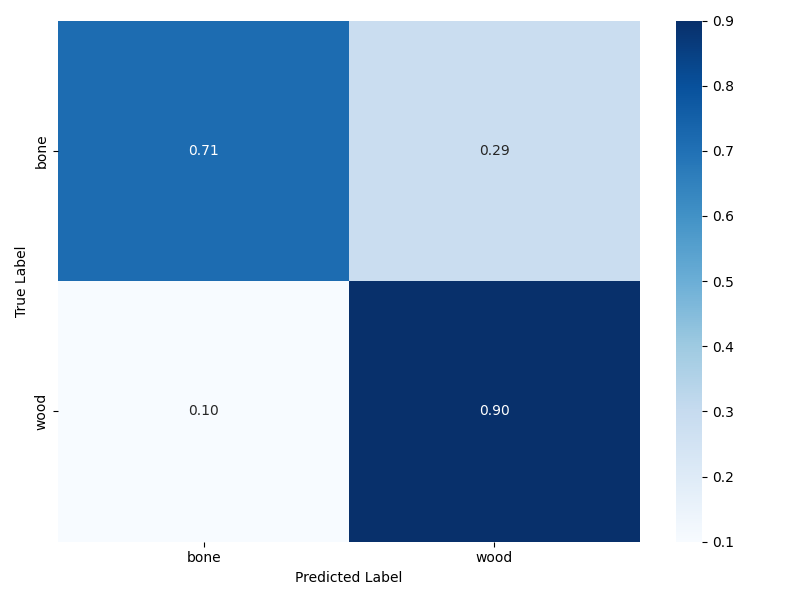

Supplement: Supplementary file 1 — Supplementary Material 1 [file 41598_2025_18179_MOESM1_ESM.tar › supplementary_material_resubmit1/Supplementary Figure S4/evaluation_metrics/custom/matrix_20x_1000_9.png]

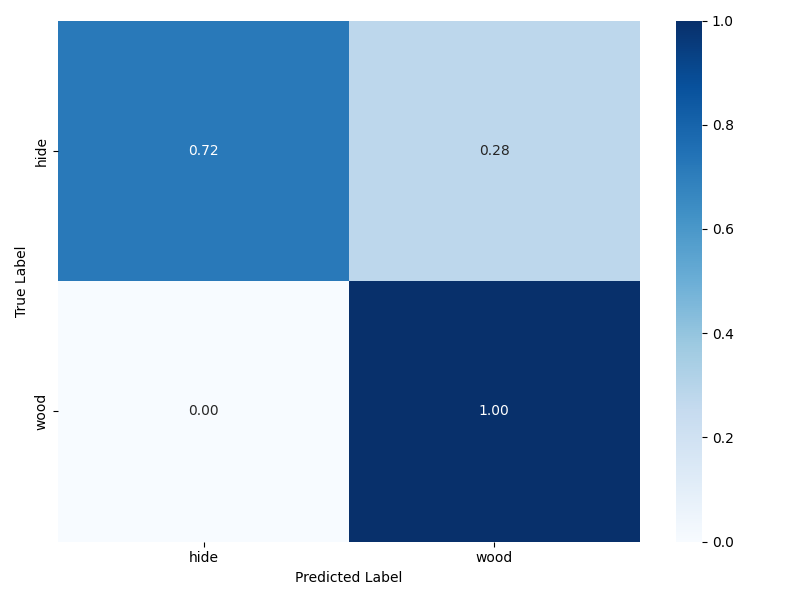

Supplement: Supplementary file 1 — Supplementary Material 1 [file 41598_2025_18179_MOESM1_ESM.tar › supplementary_material_resubmit1/Supplementary Figure S4/evaluation_metrics/custom/matrix_20x_2000_16.png]

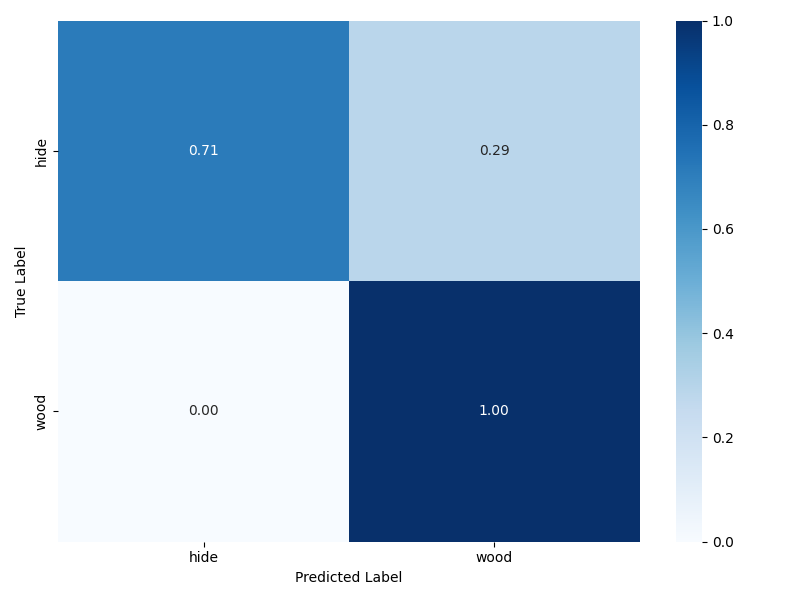

Supplement: Supplementary file 1 — Supplementary Material 1 [file 41598_2025_18179_MOESM1_ESM.tar › supplementary_material_resubmit1/Supplementary Figure S4/evaluation_metrics/custom/matrix_20x_2000_9.png]

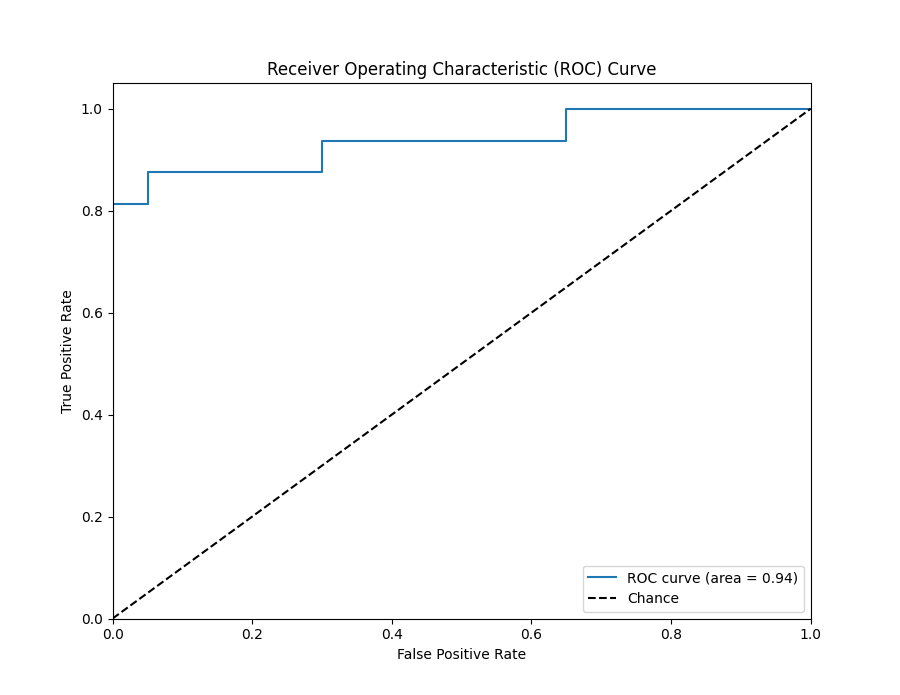

Supplement: Supplementary file 1 — Supplementary Material 1 [file 41598_2025_18179_MOESM1_ESM.tar › supplementary_material_resubmit1/Supplementary Figure S4/evaluation_metrics/custom/roc_10x_1000_16.png]

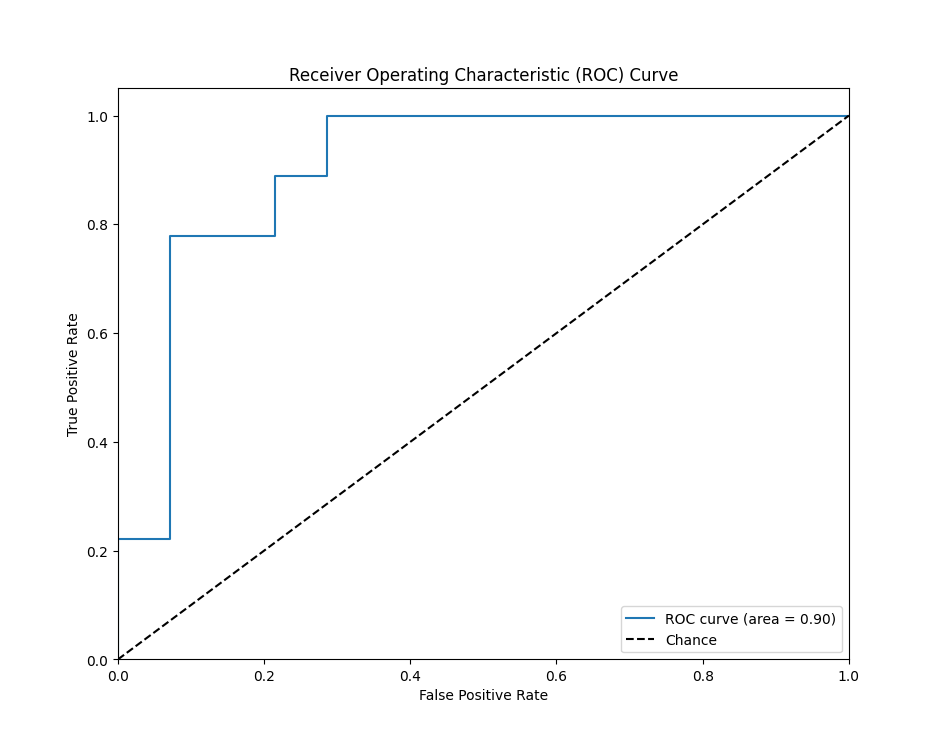

Supplement: Supplementary file 1 — Supplementary Material 1 [file 41598_2025_18179_MOESM1_ESM.tar › supplementary_material_resubmit1/Supplementary Figure S4/evaluation_metrics/custom/roc_10x_2000_9.png]

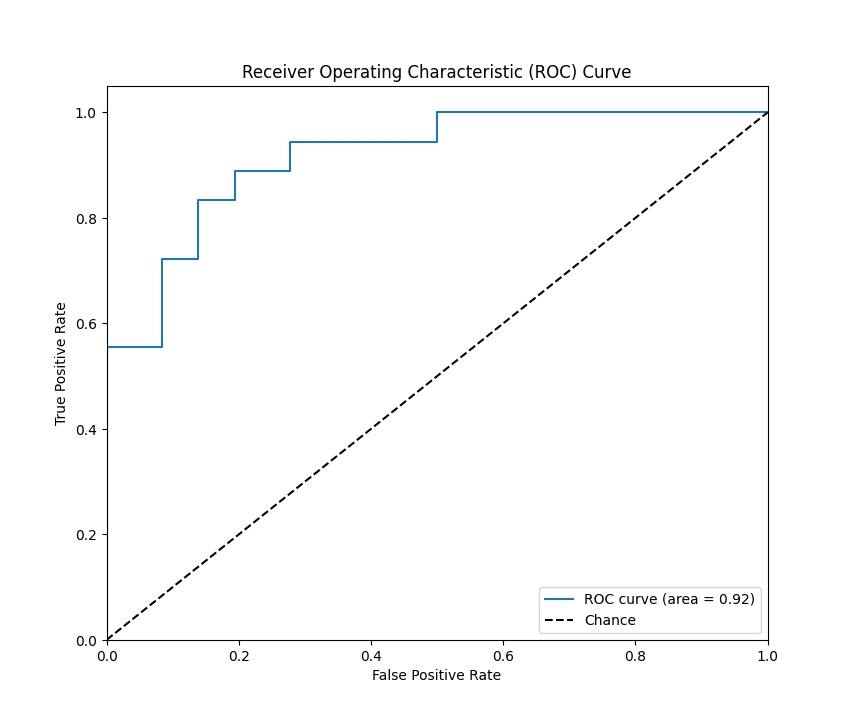

Supplement: Supplementary file 1 — Supplementary Material 1 [file 41598_2025_18179_MOESM1_ESM.tar › supplementary_material_resubmit1/Supplementary Figure S4/evaluation_metrics/custom/roc_20x_1000_16.png]

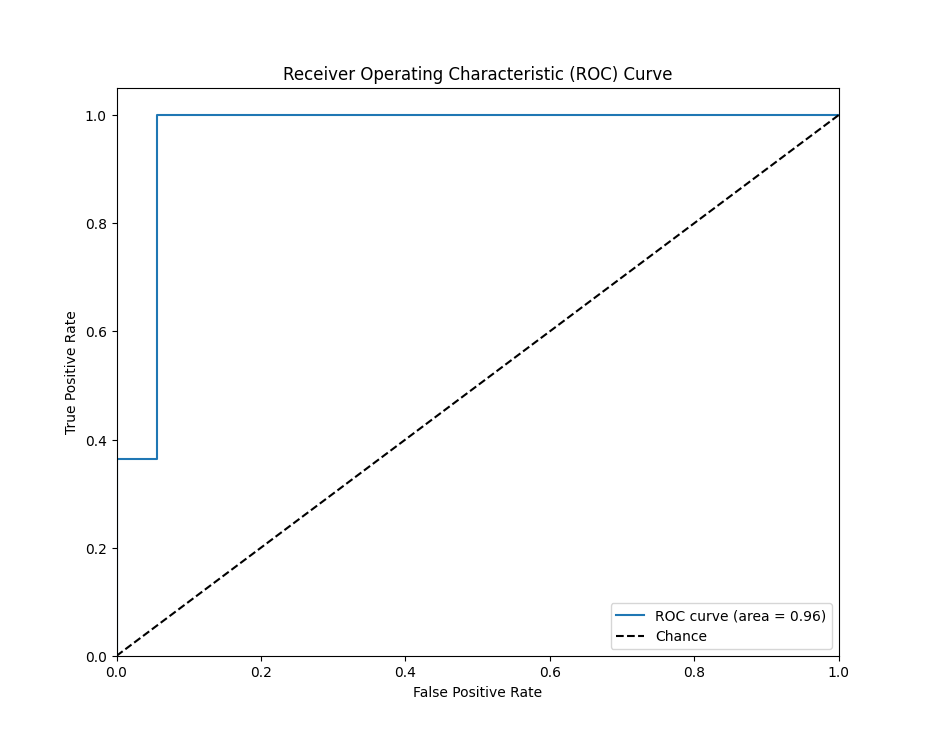

Supplement: Supplementary file 1 — Supplementary Material 1 [file 41598_2025_18179_MOESM1_ESM.tar › supplementary_material_resubmit1/Supplementary Figure S4/evaluation_metrics/custom/roc_20x_1000_2000_16.png]

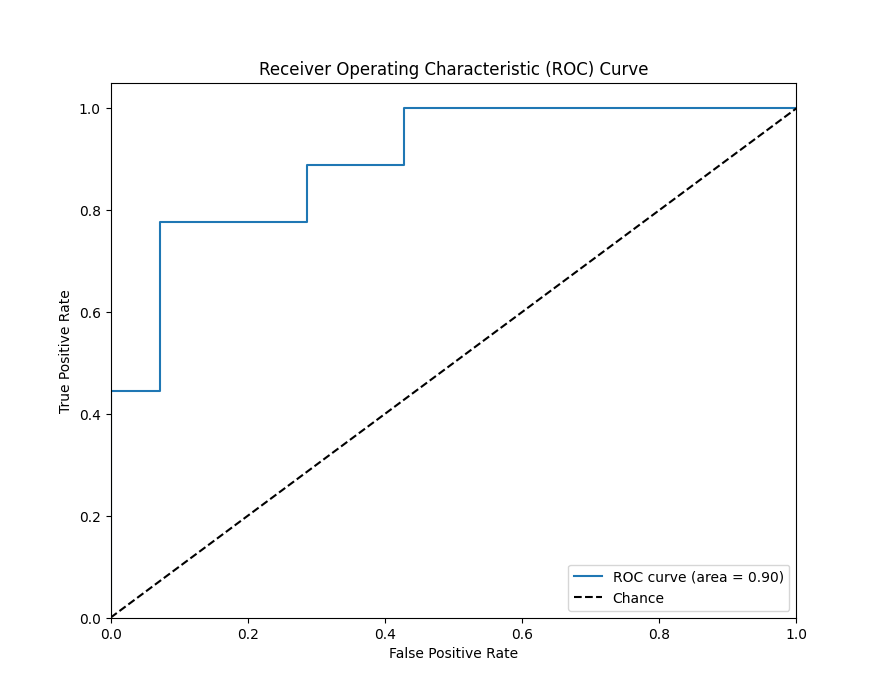

Supplement: Supplementary file 1 — Supplementary Material 1 [file 41598_2025_18179_MOESM1_ESM.tar › supplementary_material_resubmit1/Supplementary Figure S4/evaluation_metrics/custom/roc_20x_1000_2000_9.png]

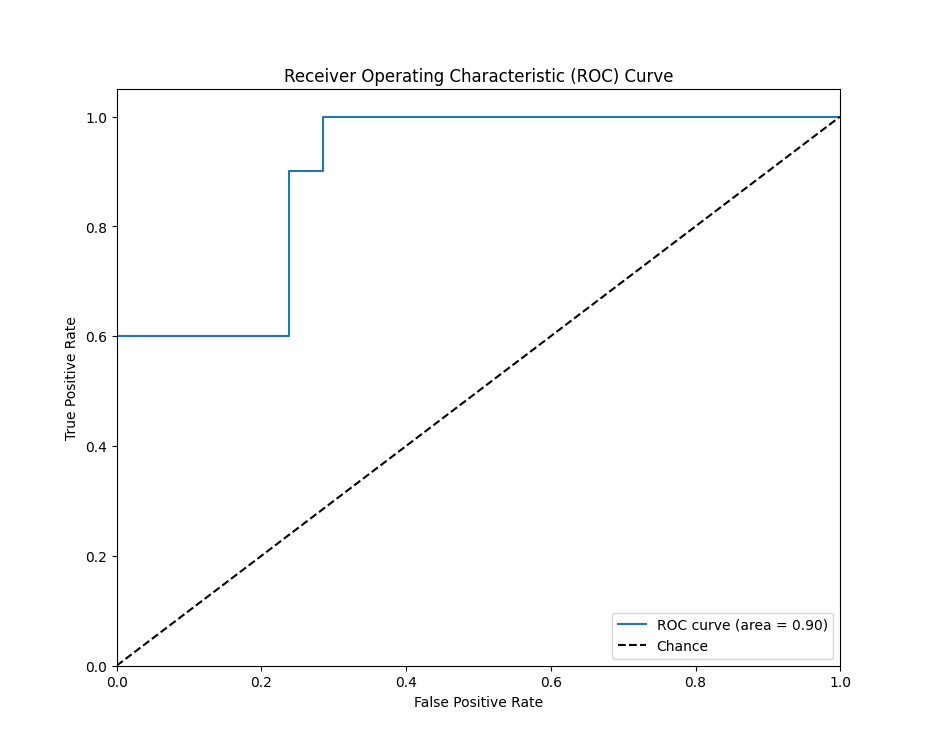

Supplement: Supplementary file 1 — Supplementary Material 1 [file 41598_2025_18179_MOESM1_ESM.tar › supplementary_material_resubmit1/Supplementary Figure S4/evaluation_metrics/custom/roc_20x_1000_9.png]

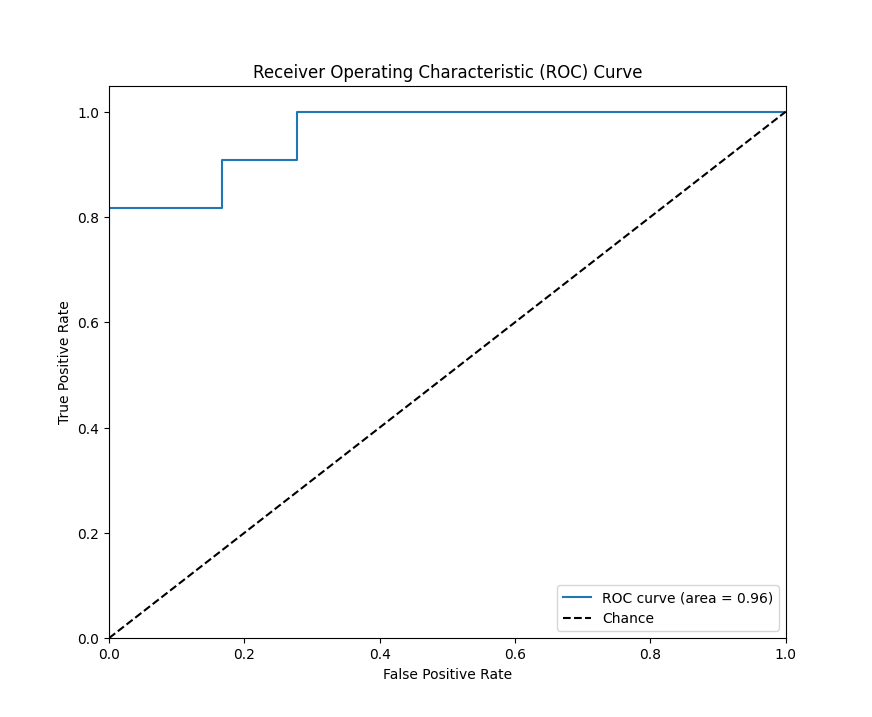

Supplement: Supplementary file 1 — Supplementary Material 1 [file 41598_2025_18179_MOESM1_ESM.tar › supplementary_material_resubmit1/Supplementary Figure S4/evaluation_metrics/custom/roc_20x_2000_16.png]

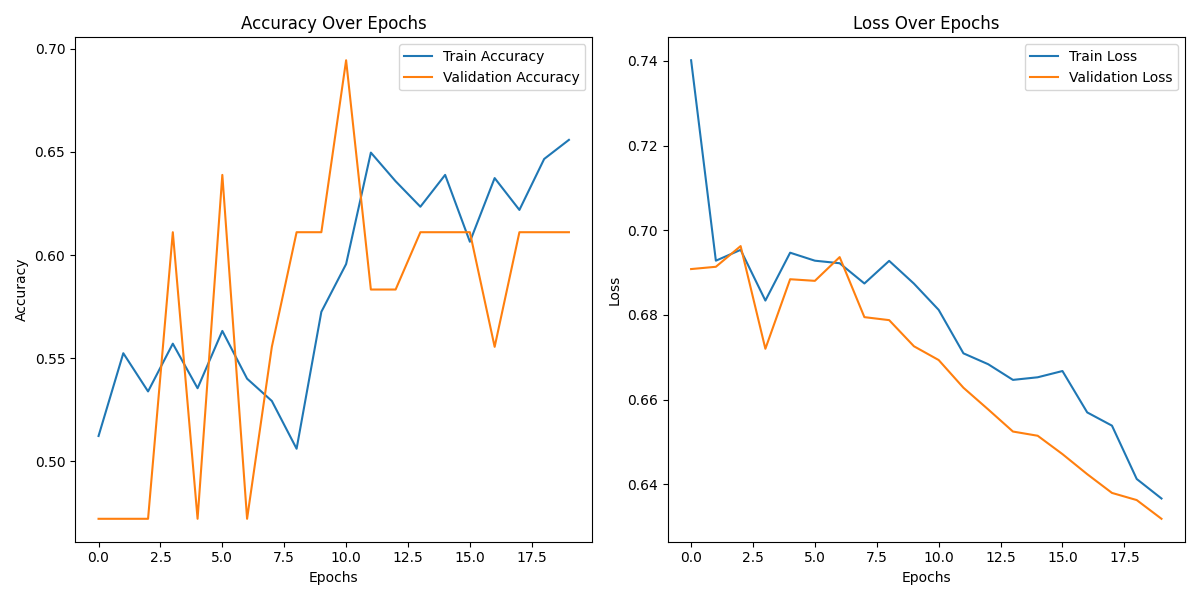

Supplement: Supplementary file 1 — Supplementary Material 1 [file 41598_2025_18179_MOESM1_ESM.tar › supplementary_material_resubmit1/Supplementary Figure S4/evaluation_metrics/ResNet/lc_10x_1000_16.png]

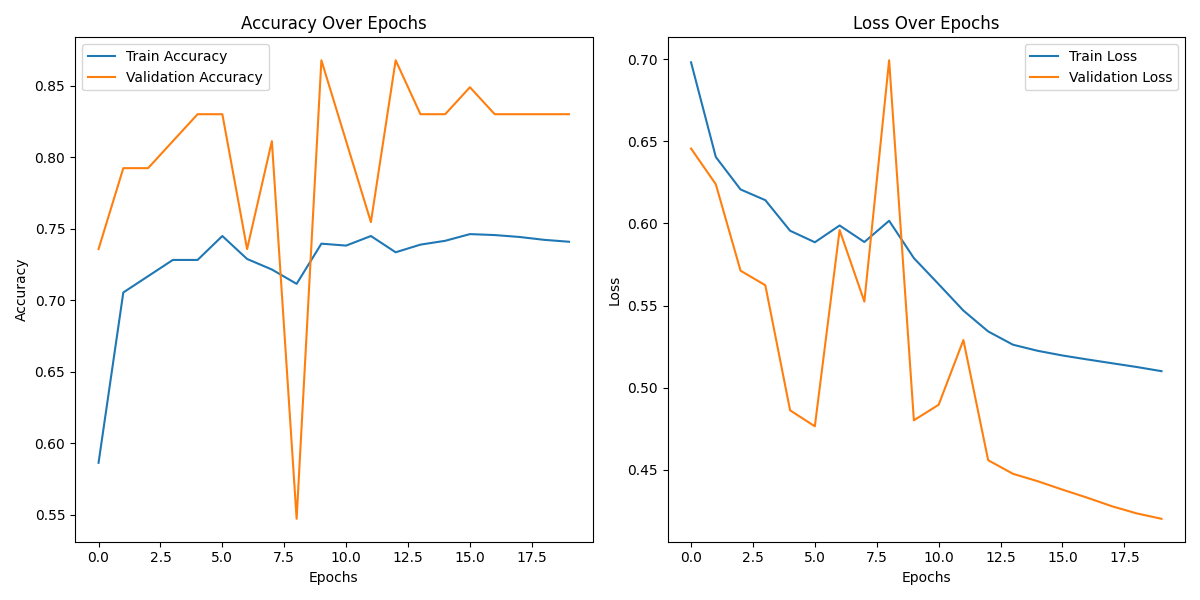

Supplement: Supplementary file 1 — Supplementary Material 1 [file 41598_2025_18179_MOESM1_ESM.tar › supplementary_material_resubmit1/Supplementary Figure S4/evaluation_metrics/ResNet/lc_20x_1000_16.png]

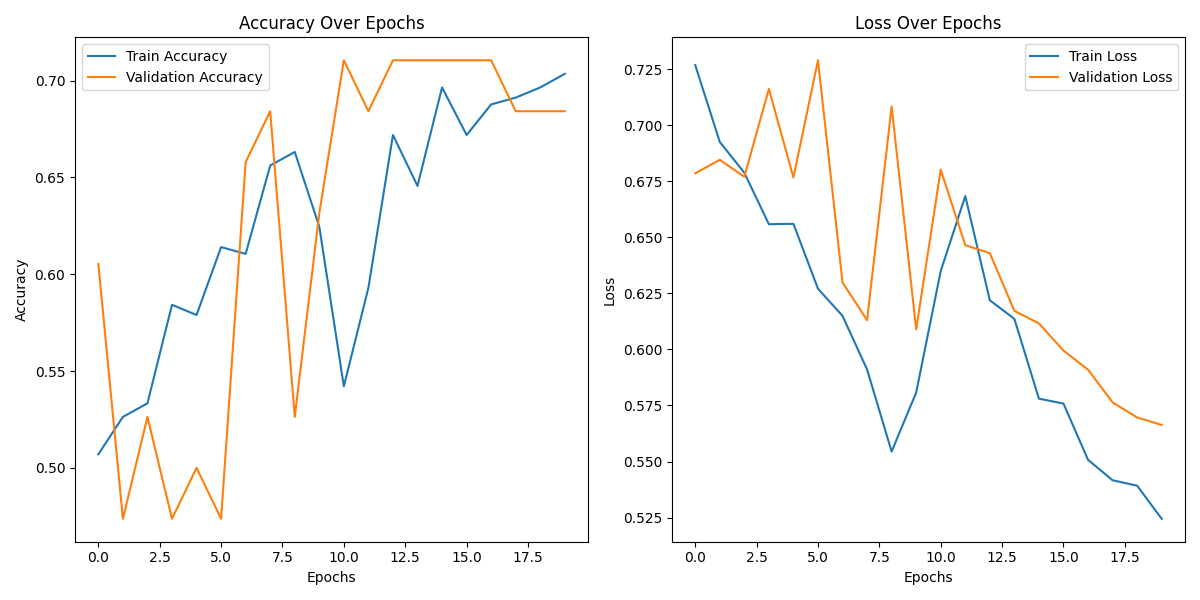

Supplement: Supplementary file 1 — Supplementary Material 1 [file 41598_2025_18179_MOESM1_ESM.tar › supplementary_material_resubmit1/Supplementary Figure S4/evaluation_metrics/ResNet/lc_20x_1000_2000_16.png]

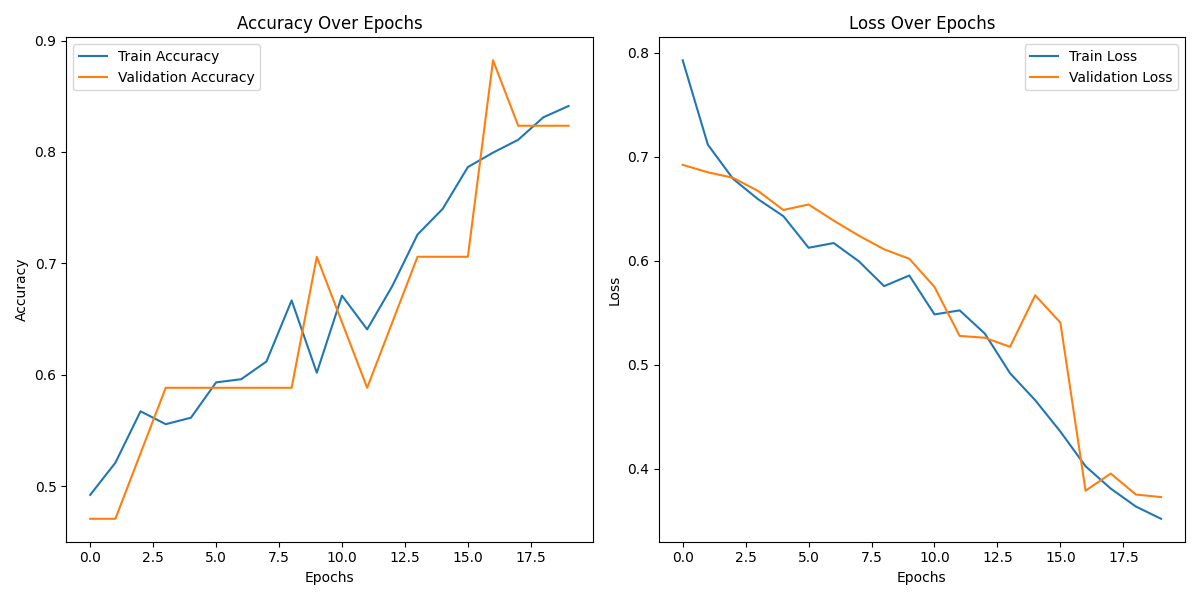

Supplement: Supplementary file 1 — Supplementary Material 1 [file 41598_2025_18179_MOESM1_ESM.tar › supplementary_material_resubmit1/Supplementary Figure S4/evaluation_metrics/ResNet/lc_20x_1000_2000_9.png]

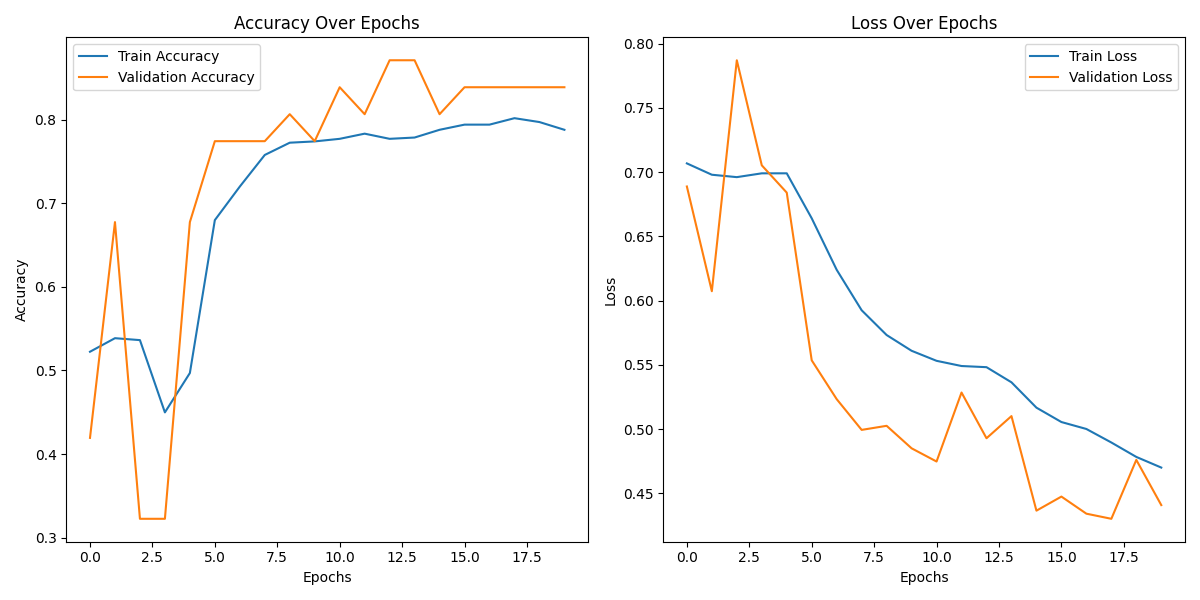

Supplement: Supplementary file 1 — Supplementary Material 1 [file 41598_2025_18179_MOESM1_ESM.tar › supplementary_material_resubmit1/Supplementary Figure S4/evaluation_metrics/ResNet/lc_20x_1000_9.png]

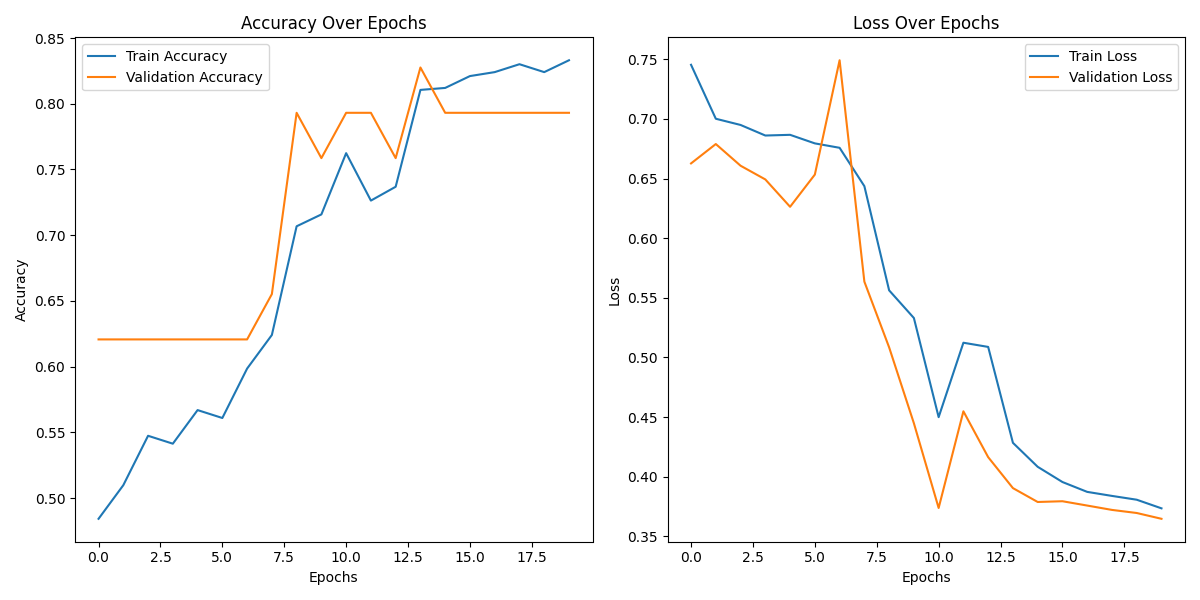

Supplement: Supplementary file 1 — Supplementary Material 1 [file 41598_2025_18179_MOESM1_ESM.tar › supplementary_material_resubmit1/Supplementary Figure S4/evaluation_metrics/ResNet/lc_20x_2000_16.png]

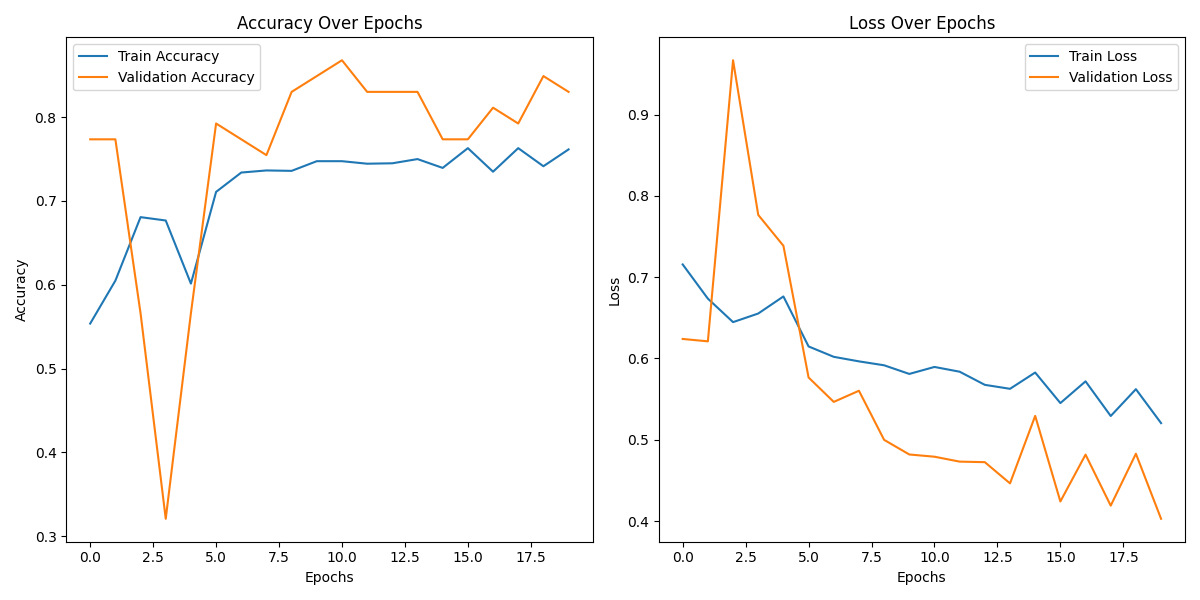

Supplement: Supplementary file 1 — Supplementary Material 1 [file 41598_2025_18179_MOESM1_ESM.tar › supplementary_material_resubmit1/Supplementary Figure S4/evaluation_metrics/ResNet/lc_20x_2000_9.png]

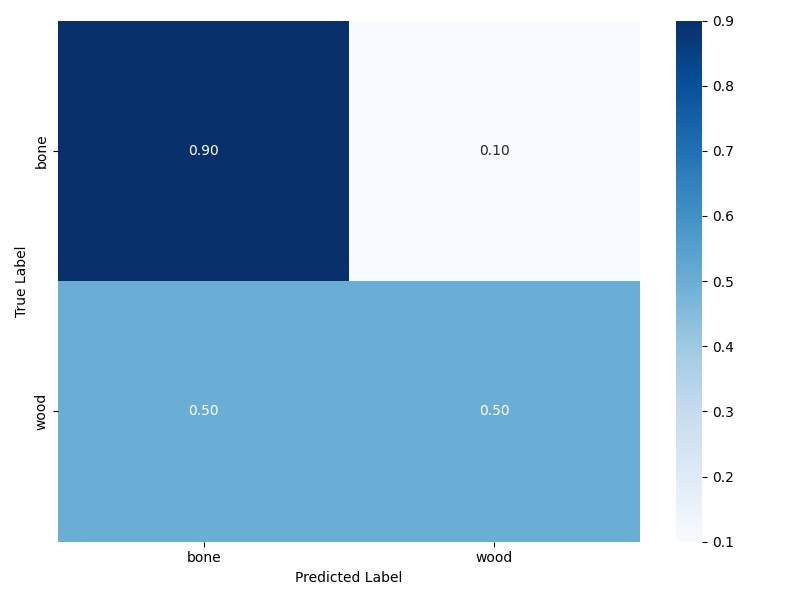

Supplement: Supplementary file 1 — Supplementary Material 1 [file 41598_2025_18179_MOESM1_ESM.tar › supplementary_material_resubmit1/Supplementary Figure S4/evaluation_metrics/ResNet/matrix_10x_1000_16.png]

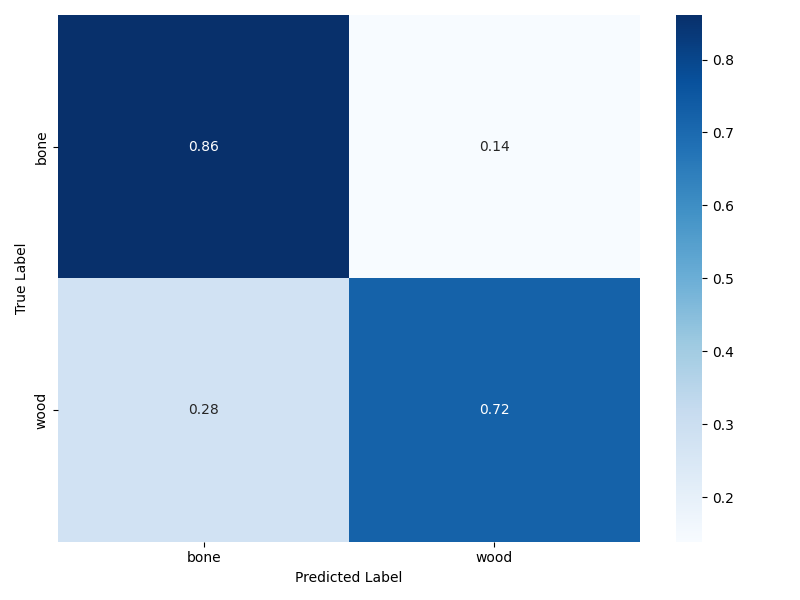

Supplement: Supplementary file 1 — Supplementary Material 1 [file 41598_2025_18179_MOESM1_ESM.tar › supplementary_material_resubmit1/Supplementary Figure S4/evaluation_metrics/ResNet/matrix_20x_1000_16.png]

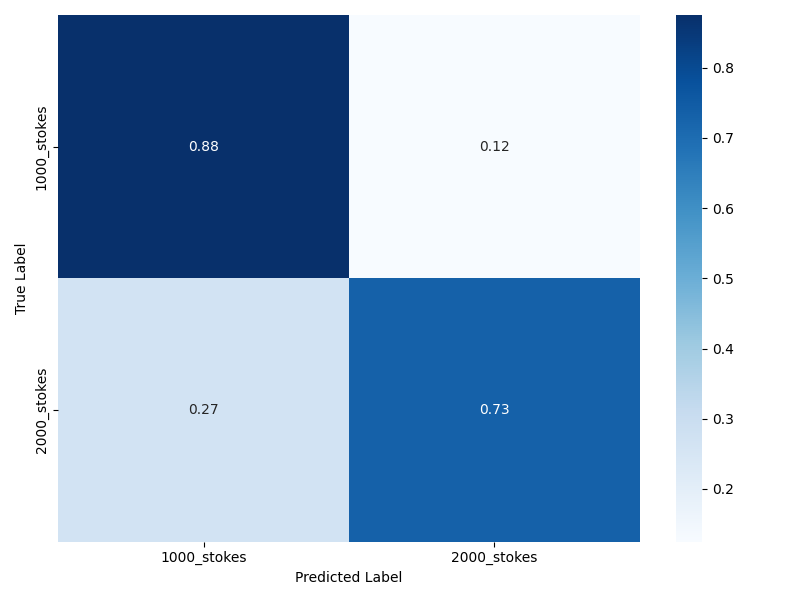

Supplement: Supplementary file 1 — Supplementary Material 1 [file 41598_2025_18179_MOESM1_ESM.tar › supplementary_material_resubmit1/Supplementary Figure S4/evaluation_metrics/ResNet/matrix_20x_1000_2000_16.png]

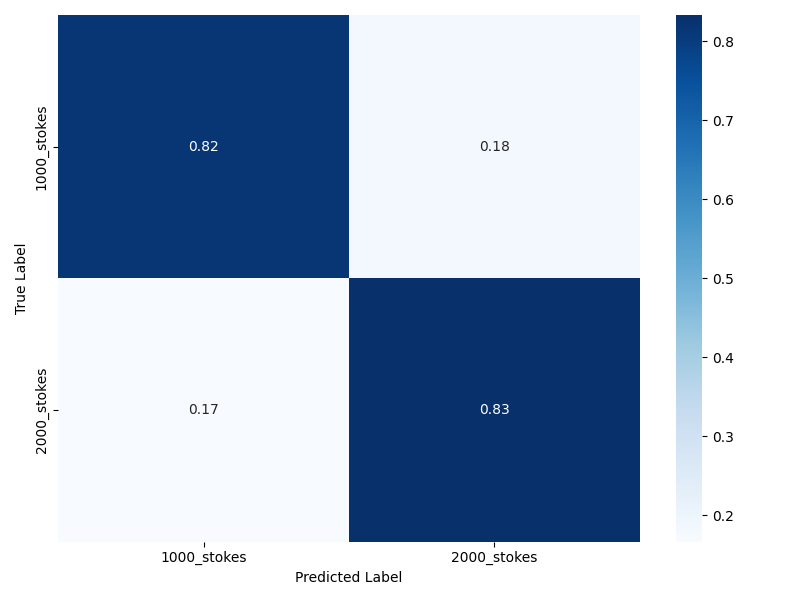

Supplement: Supplementary file 1 — Supplementary Material 1 [file 41598_2025_18179_MOESM1_ESM.tar › supplementary_material_resubmit1/Supplementary Figure S4/evaluation_metrics/ResNet/matrix_20x_1000_2000_9.png]

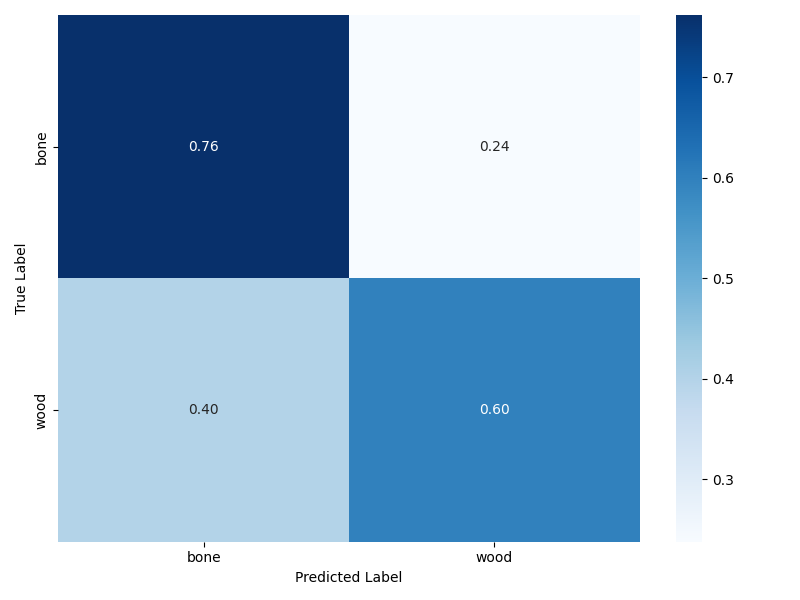

Supplement: Supplementary file 1 — Supplementary Material 1 [file 41598_2025_18179_MOESM1_ESM.tar › supplementary_material_resubmit1/Supplementary Figure S4/evaluation_metrics/ResNet/matrix_20x_1000_9.png]

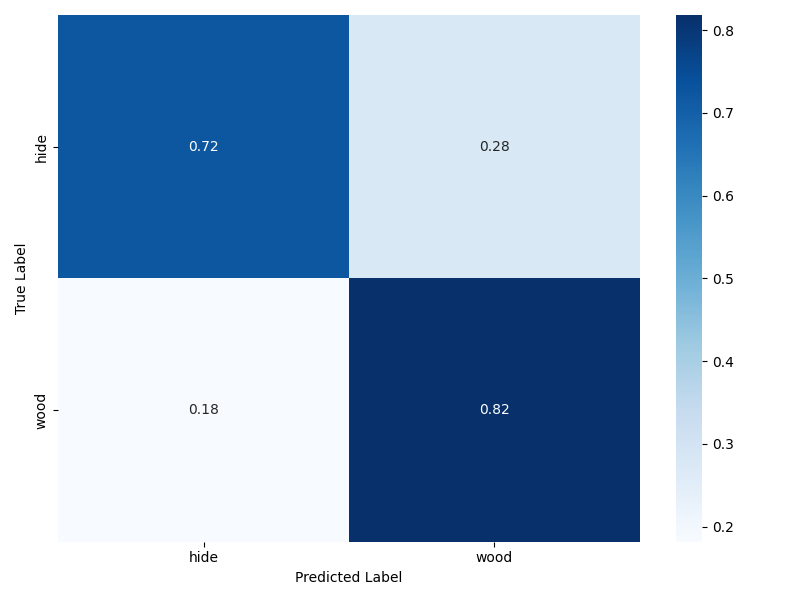

Supplement: Supplementary file 1 — Supplementary Material 1 [file 41598_2025_18179_MOESM1_ESM.tar › supplementary_material_resubmit1/Supplementary Figure S4/evaluation_metrics/ResNet/matrix_20x_2000_16.png]

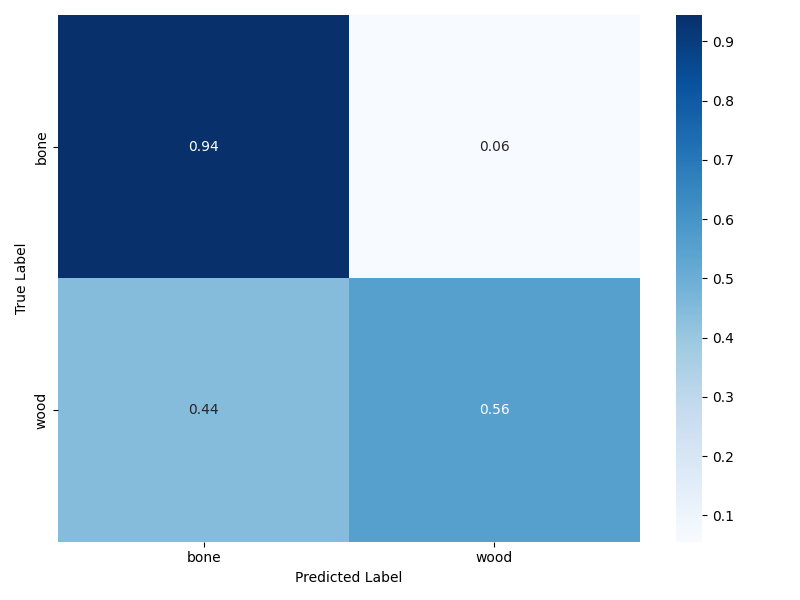

Supplement: Supplementary file 1 — Supplementary Material 1 [file 41598_2025_18179_MOESM1_ESM.tar › supplementary_material_resubmit1/Supplementary Figure S4/evaluation_metrics/ResNet/matrix_20x_2000_9.png]

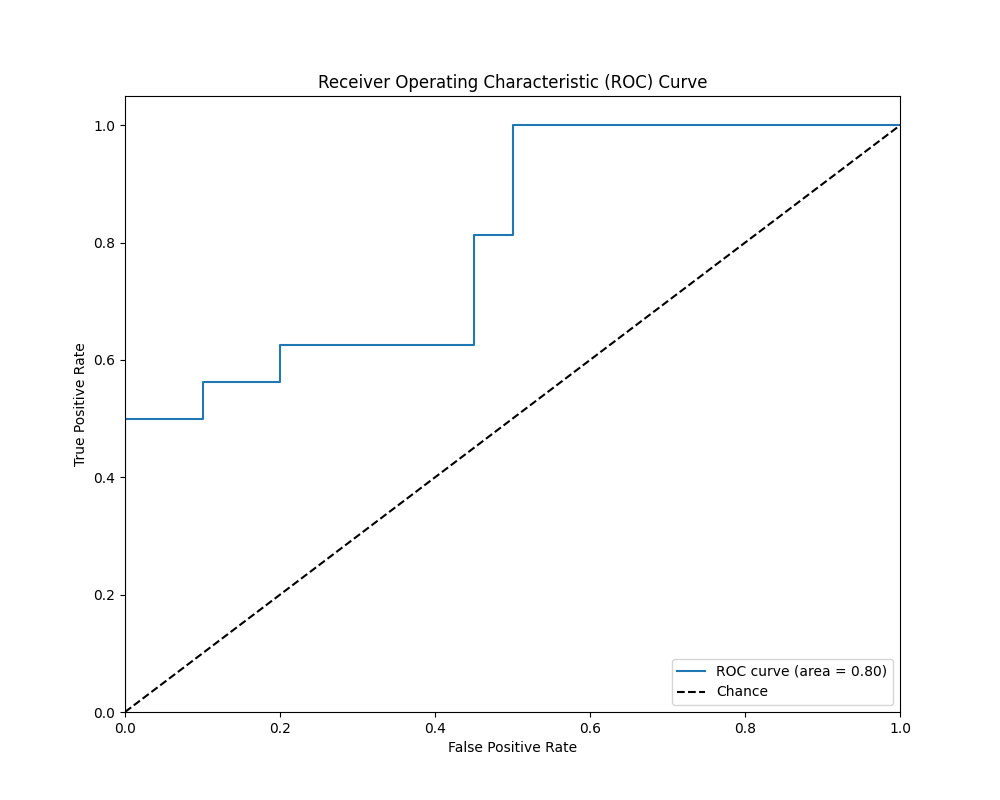

Supplement: Supplementary file 1 — Supplementary Material 1 [file 41598_2025_18179_MOESM1_ESM.tar › supplementary_material_resubmit1/Supplementary Figure S4/evaluation_metrics/ResNet/roc_10x_1000_16.png]

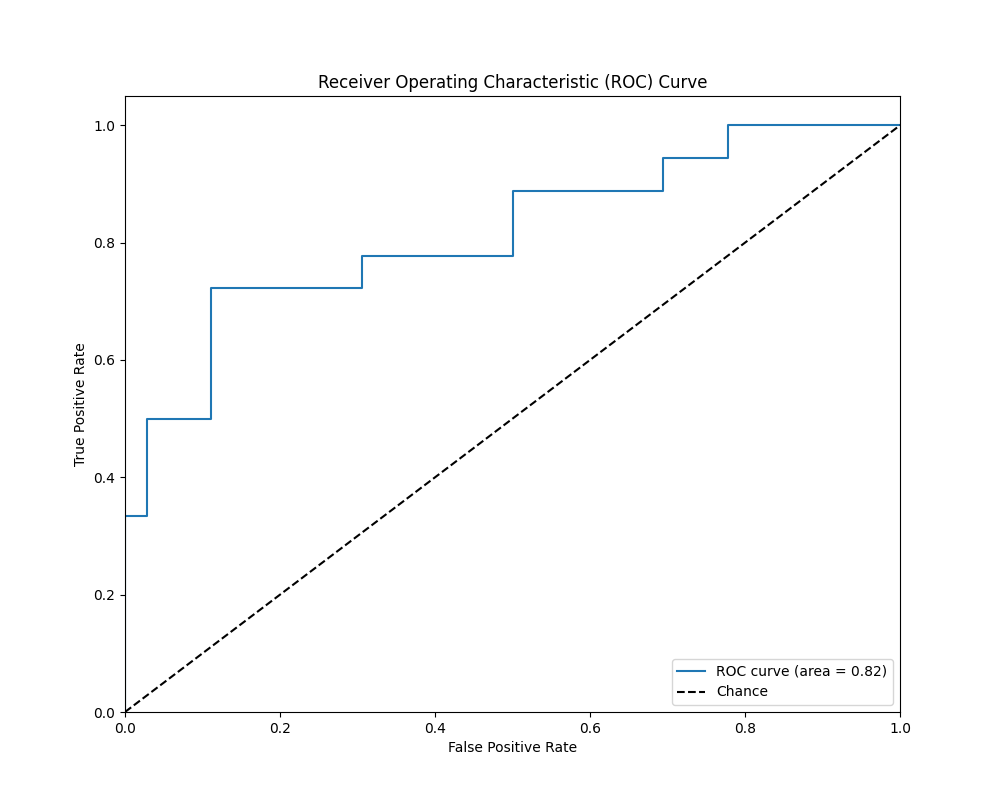

Supplement: Supplementary file 1 — Supplementary Material 1 [file 41598_2025_18179_MOESM1_ESM.tar › supplementary_material_resubmit1/Supplementary Figure S4/evaluation_metrics/ResNet/roc_20x_1000_16.png]

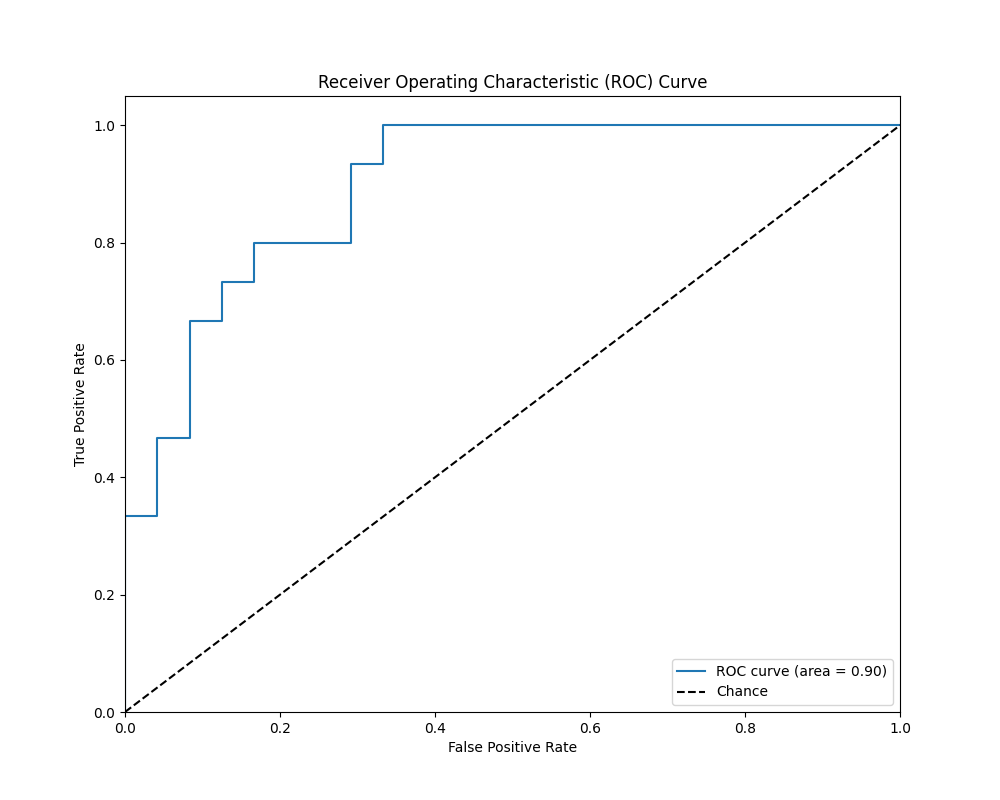

Supplement: Supplementary file 1 — Supplementary Material 1 [file 41598_2025_18179_MOESM1_ESM.tar › supplementary_material_resubmit1/Supplementary Figure S4/evaluation_metrics/ResNet/roc_20x_1000_2000_16.png]

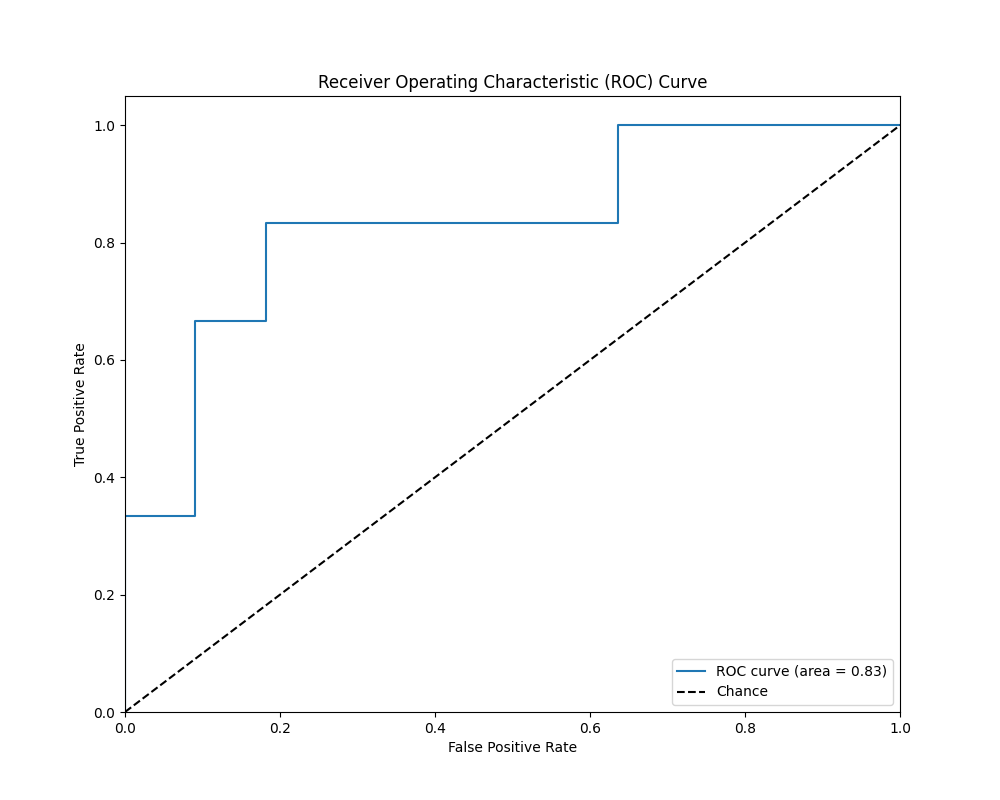

Supplement: Supplementary file 1 — Supplementary Material 1 [file 41598_2025_18179_MOESM1_ESM.tar › supplementary_material_resubmit1/Supplementary Figure S4/evaluation_metrics/ResNet/roc_20x_1000_2000_9.png]

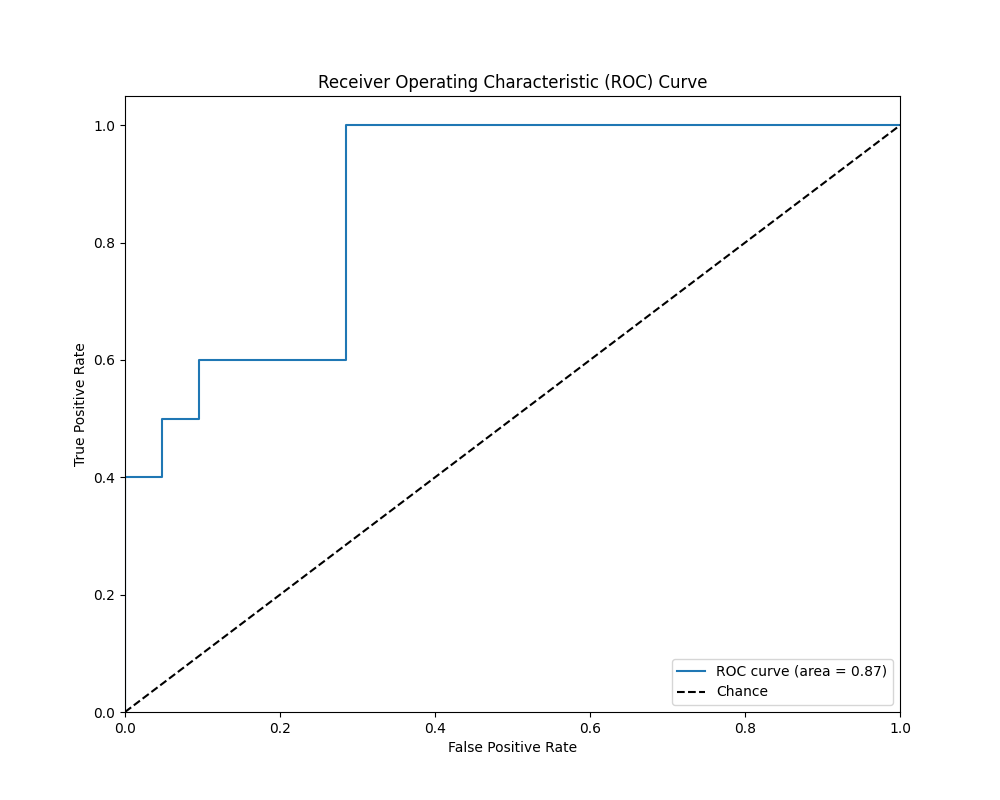

Supplement: Supplementary file 1 — Supplementary Material 1 [file 41598_2025_18179_MOESM1_ESM.tar › supplementary_material_resubmit1/Supplementary Figure S4/evaluation_metrics/ResNet/roc_20x_1000_9.png]

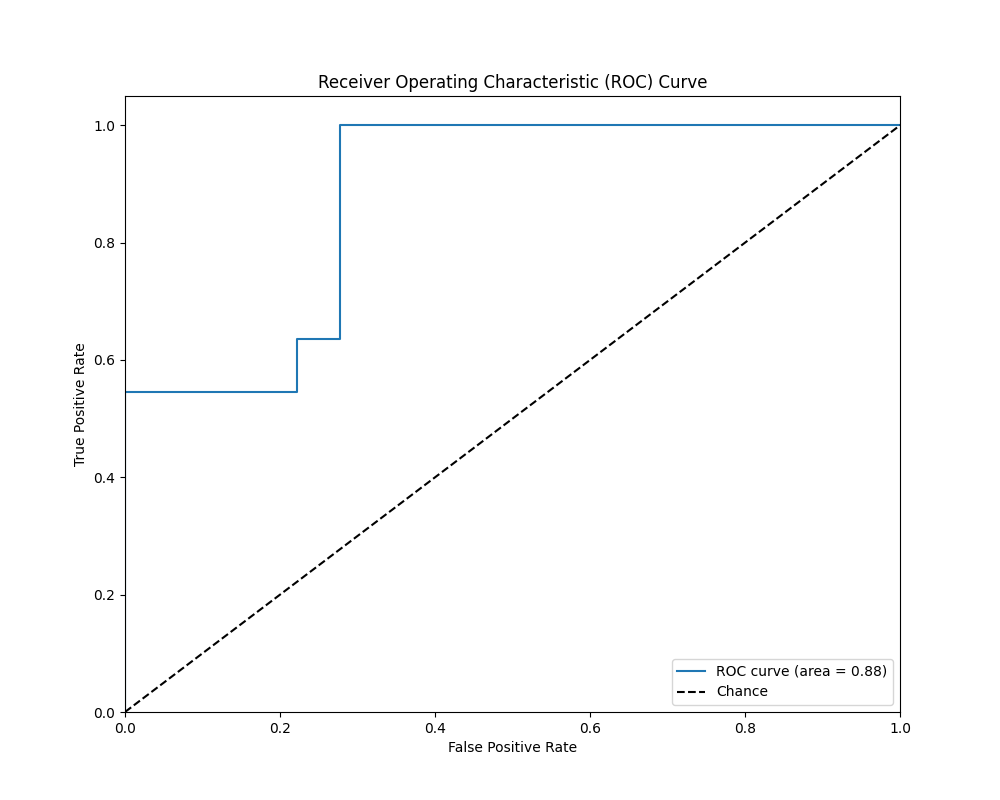

Supplement: Supplementary file 1 — Supplementary Material 1 [file 41598_2025_18179_MOESM1_ESM.tar › supplementary_material_resubmit1/Supplementary Figure S4/evaluation_metrics/ResNet/roc_20x_2000_16.png]

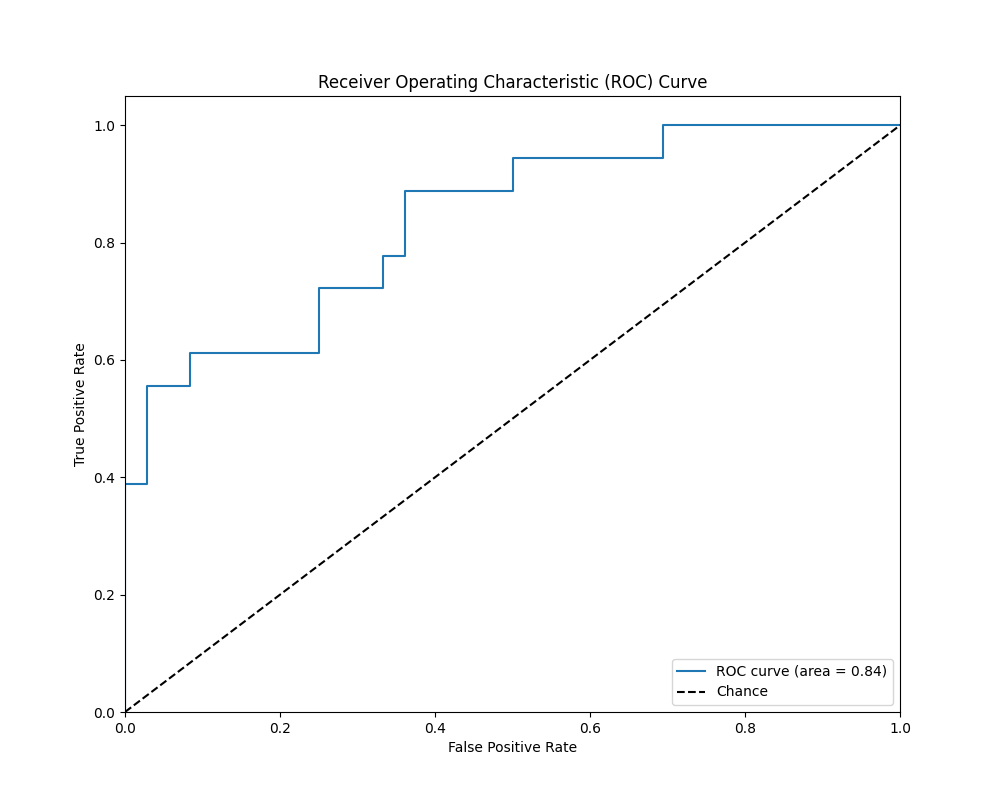

Supplement: Supplementary file 1 — Supplementary Material 1 [file 41598_2025_18179_MOESM1_ESM.tar › supplementary_material_resubmit1/Supplementary Figure S4/evaluation_metrics/ResNet/roc_20x_2000_9.png]

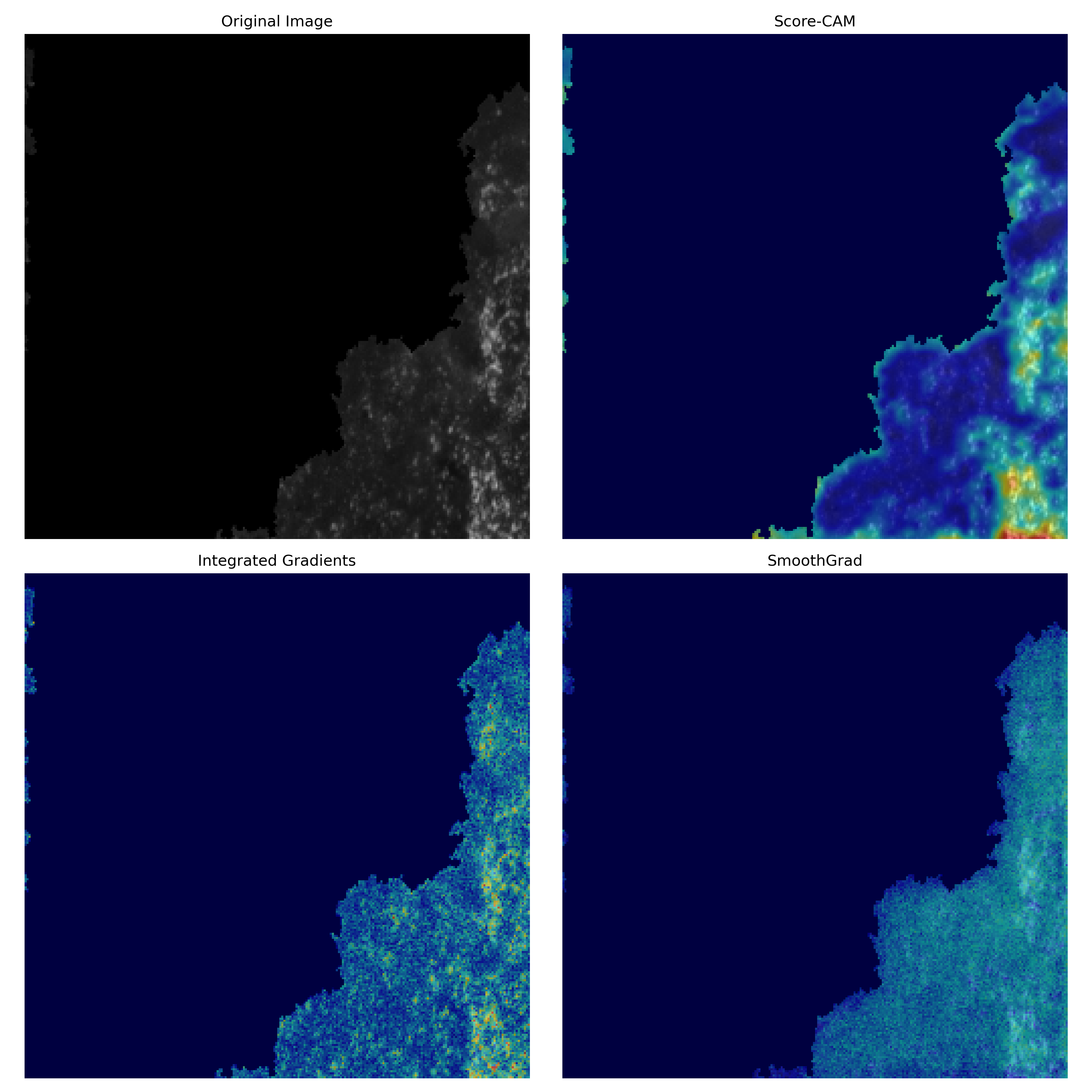

Supplement: Supplementary file 1 — Supplementary Material 1 [file 41598_2025_18179_MOESM1_ESM.tar › supplementary_material_resubmit1/Supplementary Figure S4/saliency maps/custom_CNN/100x_1000_16/bone_chichaoua_flint_CT_1000_area_2_area_2_x100_1_quadrant_2.tif_visualization.png]

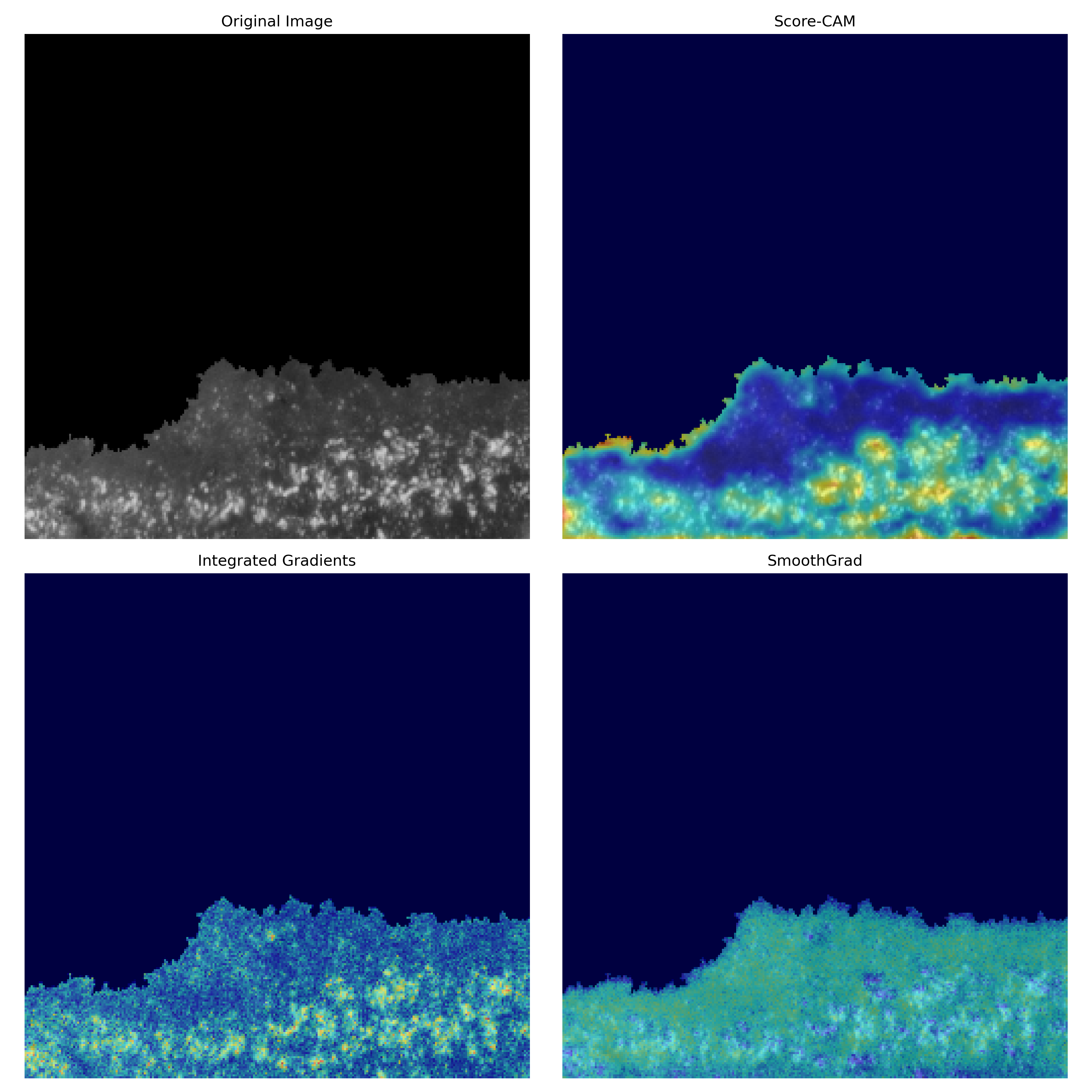

Supplement: Supplementary file 1 — Supplementary Material 1 [file 41598_2025_18179_MOESM1_ESM.tar › supplementary_material_resubmit1/Supplementary Figure S4/saliency maps/custom_CNN/100x_1000_16/bone_chichaoua_flint_CT_1000_area_4_area_2_x100_1_quadrant_1.tif_visualization.png]

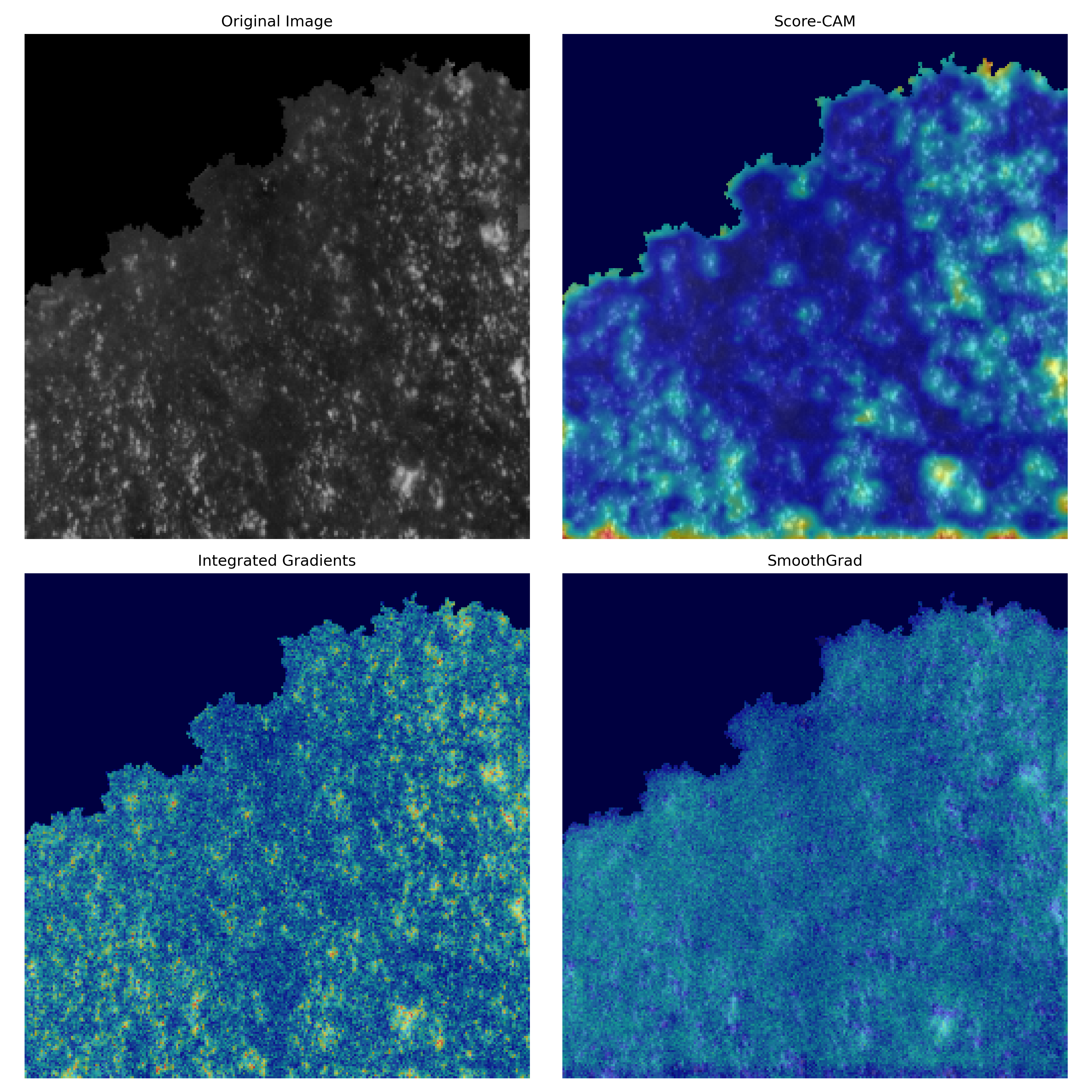

Supplement: Supplementary file 1 — Supplementary Material 1 [file 41598_2025_18179_MOESM1_ESM.tar › supplementary_material_resubmit1/Supplementary Figure S4/saliency maps/custom_CNN/100x_1000_16/bone_chichaoua_flint_CT_1000_area_4_area_2_x100_1_quadrant_3.tif_visualization.png]

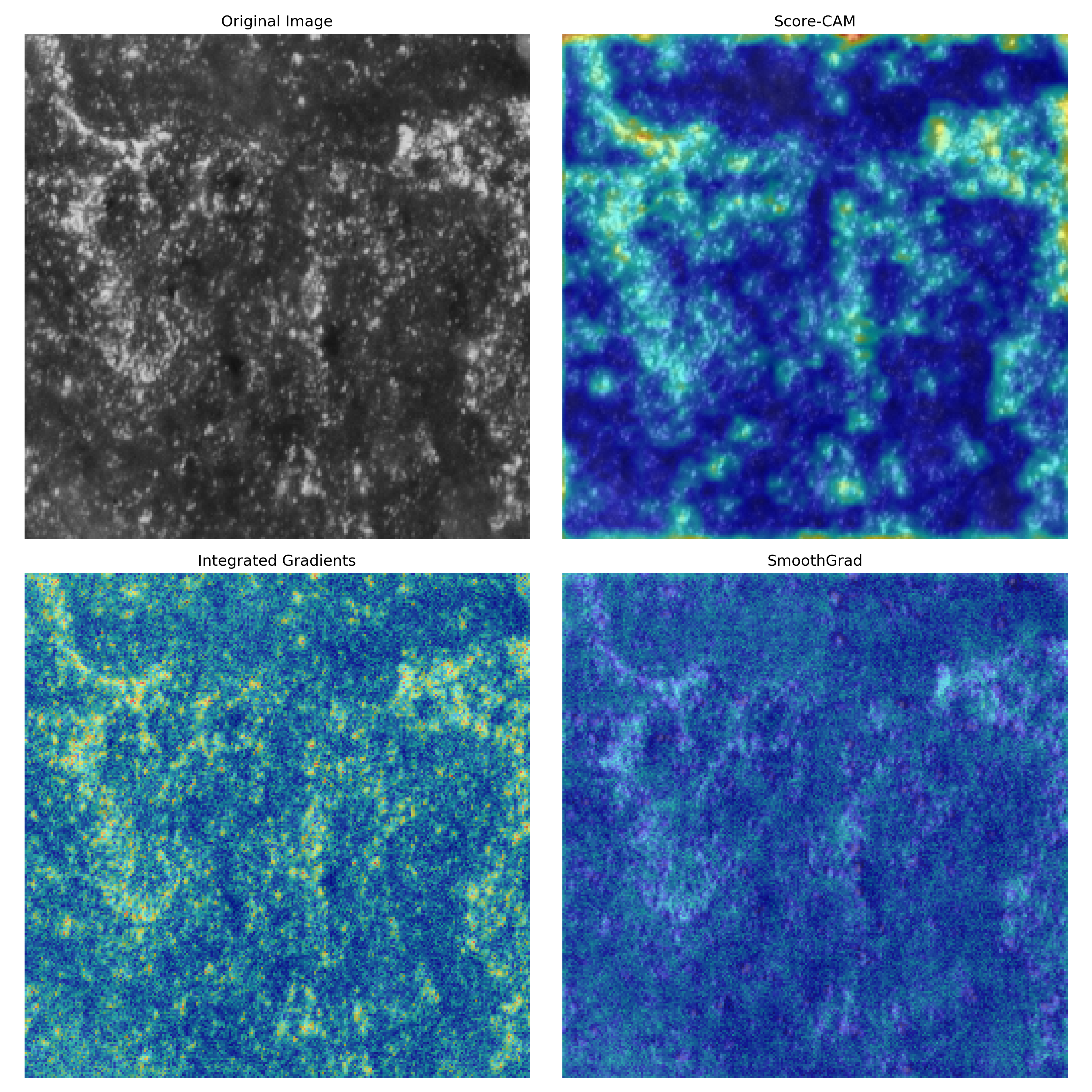

Supplement: Supplementary file 1 — Supplementary Material 1 [file 41598_2025_18179_MOESM1_ESM.tar › supplementary_material_resubmit1/Supplementary Figure S4/saliency maps/custom_CNN/100x_1000_16/bone_chichaoua_flint_CT_1000_area_4_area_2_x100_1_quadrant_5.tif_visualization.png]

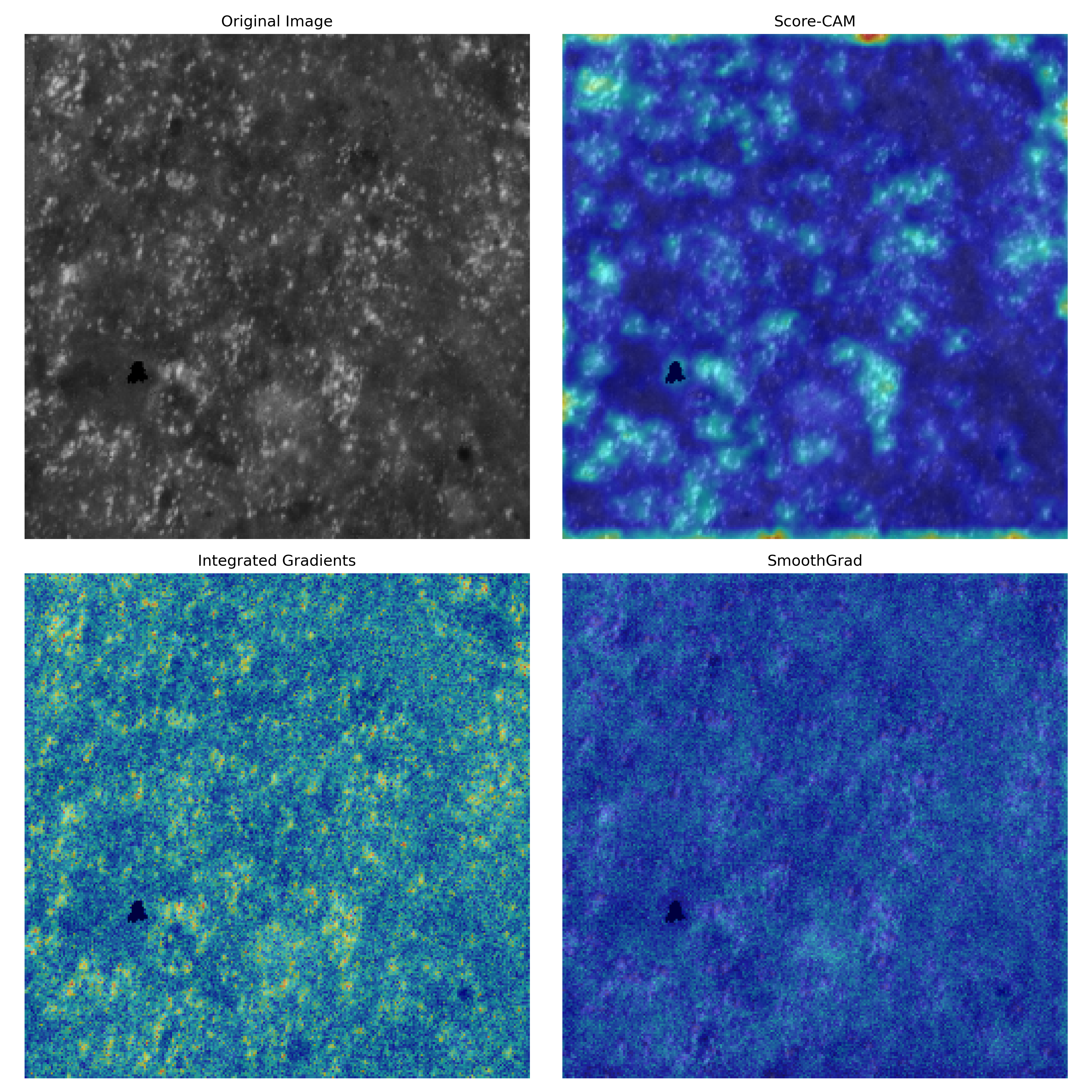

Supplement: Supplementary file 1 — Supplementary Material 1 [file 41598_2025_18179_MOESM1_ESM.tar › supplementary_material_resubmit1/Supplementary Figure S4/saliency maps/custom_CNN/100x_1000_16/bone_chichaoua_flint_CT_1000_area_4_area_2_x100_1_quadrant_8.tif_visualization.png]

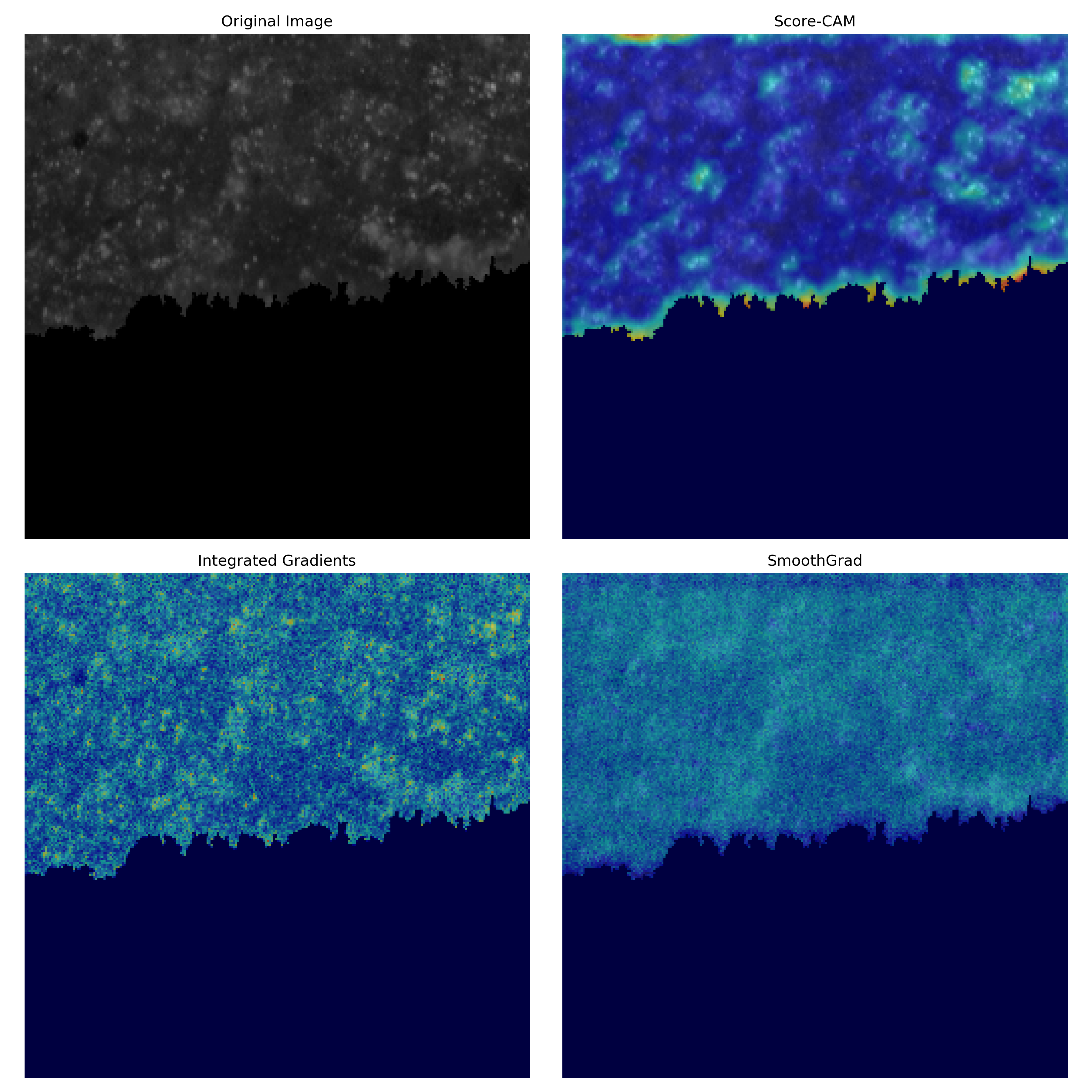

Supplement: Supplementary file 1 — Supplementary Material 1 [file 41598_2025_18179_MOESM1_ESM.tar › supplementary_material_resubmit1/Supplementary Figure S4/saliency maps/custom_CNN/100x_1000_16/bone_chichaoua_flint_SCB_1000_2_area_1_maybe_area_1_x100_1_quadrant_11.tif_visualization.png]

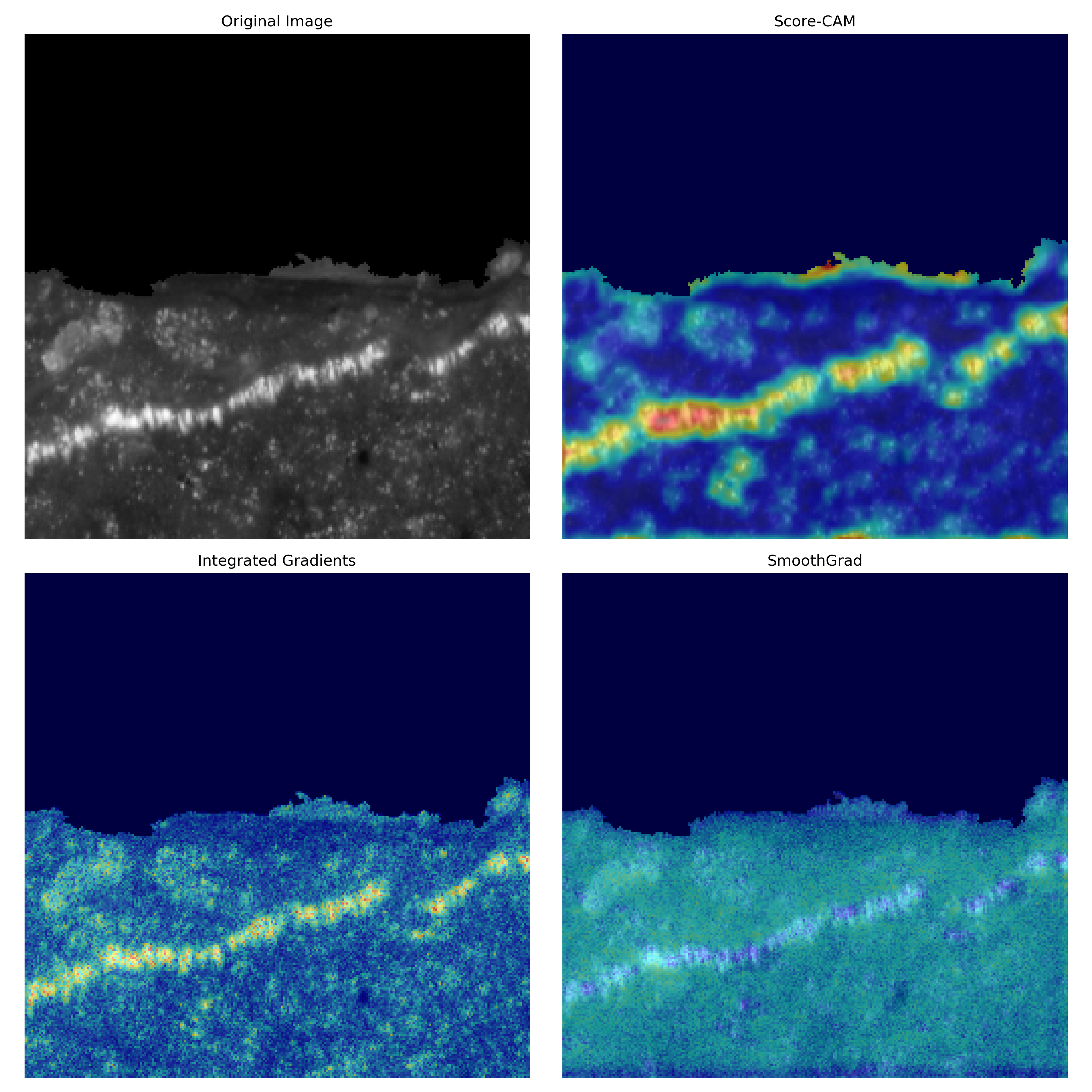

Supplement: Supplementary file 1 — Supplementary Material 1 [file 41598_2025_18179_MOESM1_ESM.tar › supplementary_material_resubmit1/Supplementary Figure S4/saliency maps/custom_CNN/100x_1000_16/bone_chichaoua_flint_SCB_1000_2_area_1_maybe_area_1_x100_1_quadrant_2.tif_visualization.png]

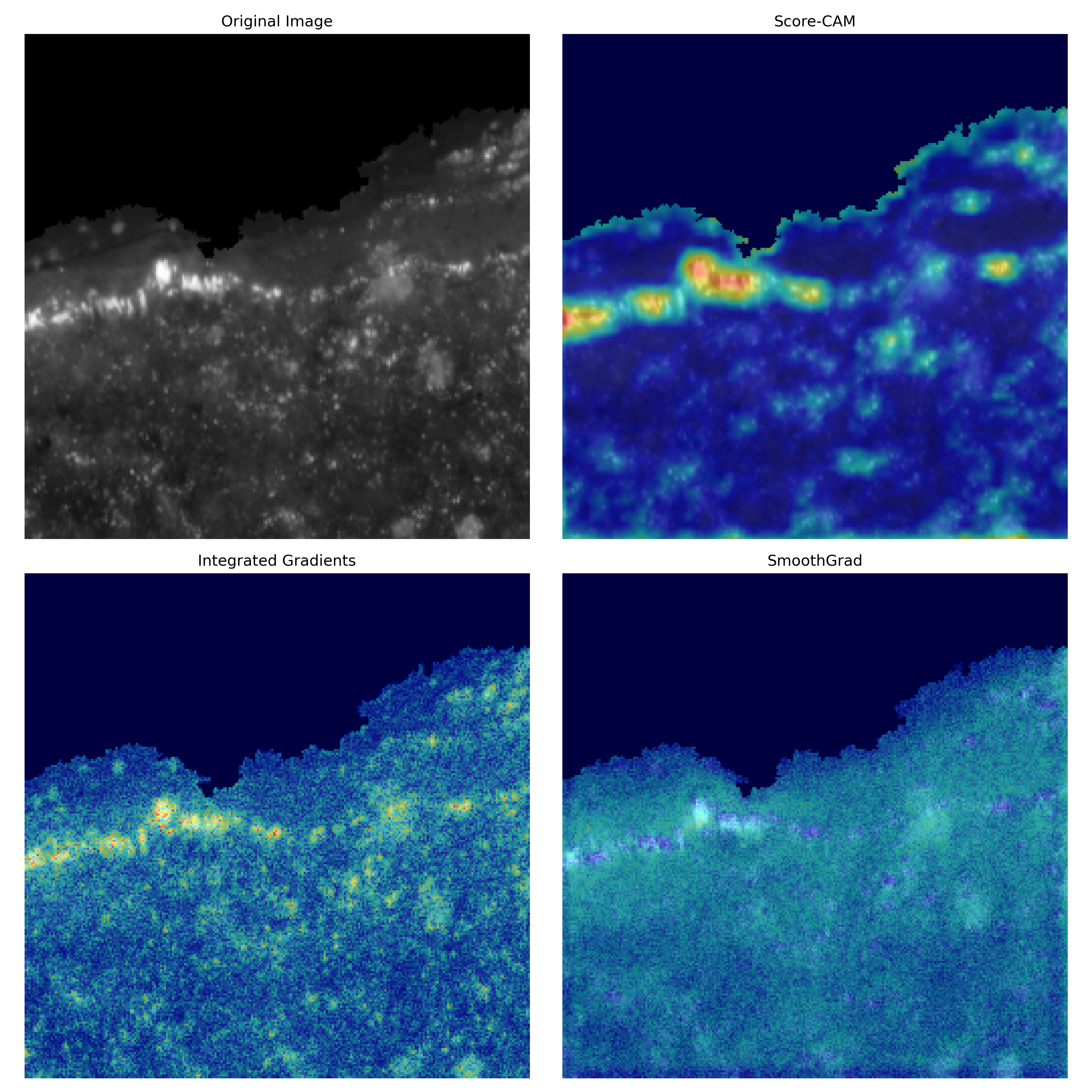

Supplement: Supplementary file 1 — Supplementary Material 1 [file 41598_2025_18179_MOESM1_ESM.tar › supplementary_material_resubmit1/Supplementary Figure S4/saliency maps/custom_CNN/100x_1000_16/bone_chichaoua_flint_SCB_1000_2_area_1_maybe_area_1_x100_1_quadrant_3.tif_visualization.png]

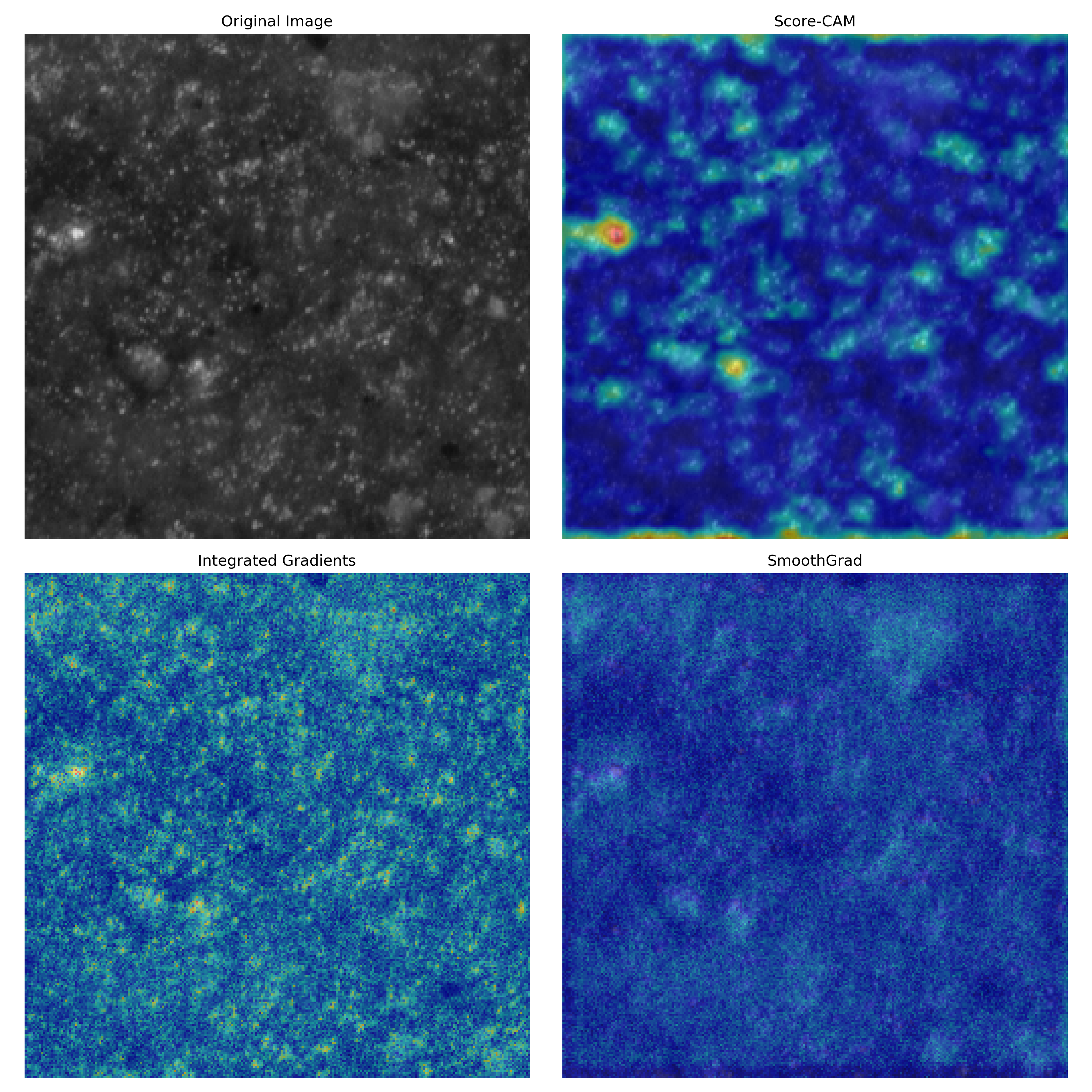

Supplement: Supplementary file 1 — Supplementary Material 1 [file 41598_2025_18179_MOESM1_ESM.tar › supplementary_material_resubmit1/Supplementary Figure S4/saliency maps/custom_CNN/100x_1000_16/bone_chichaoua_flint_SCB_1000_2_area_1_maybe_area_1_x100_1_quadrant_5.tif_visualization.png]

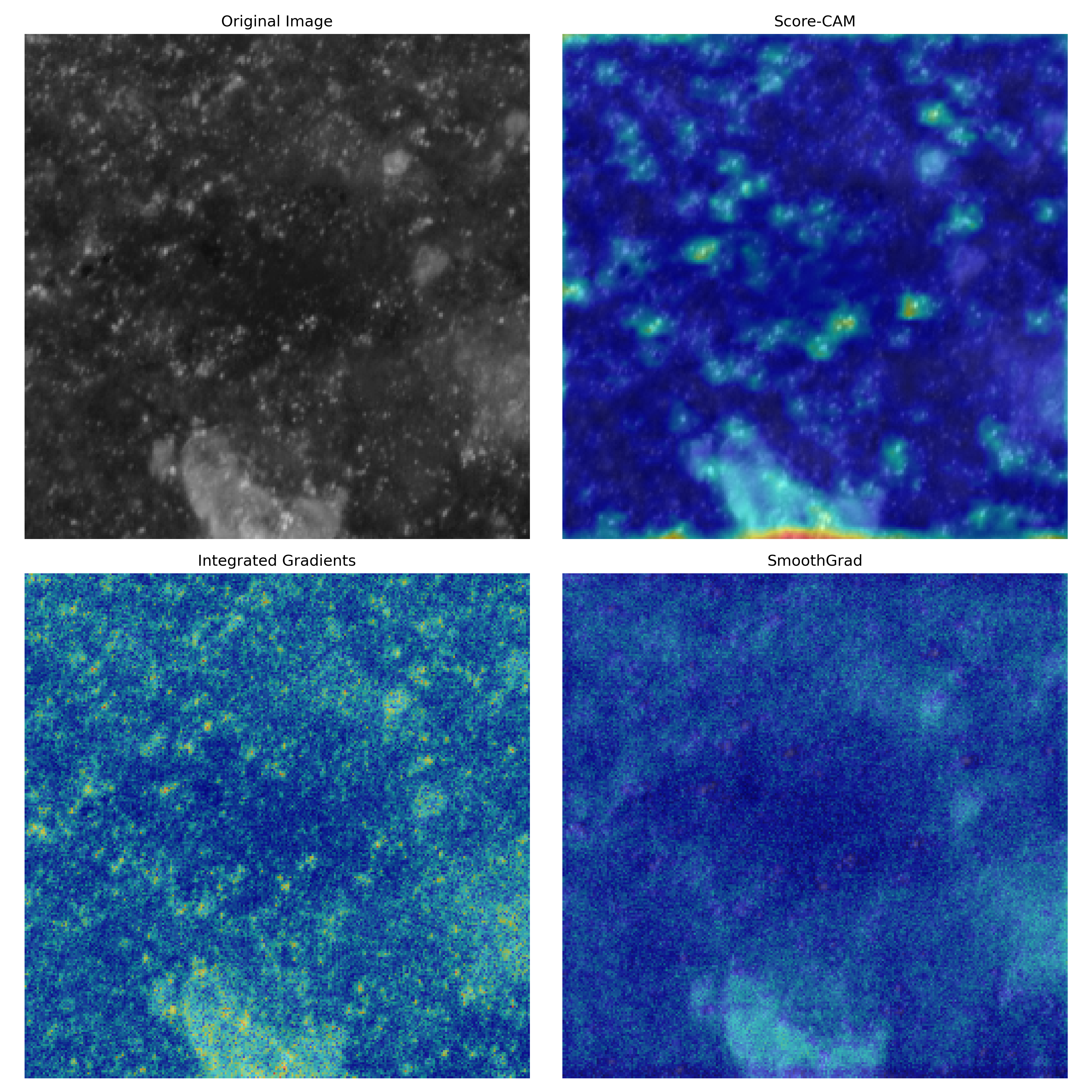

Supplement: Supplementary file 1 — Supplementary Material 1 [file 41598_2025_18179_MOESM1_ESM.tar › supplementary_material_resubmit1/Supplementary Figure S4/saliency maps/custom_CNN/100x_1000_16/bone_chichaoua_flint_SCB_1000_2_area_1_maybe_area_1_x100_1_quadrant_8.tif_visualization.png]

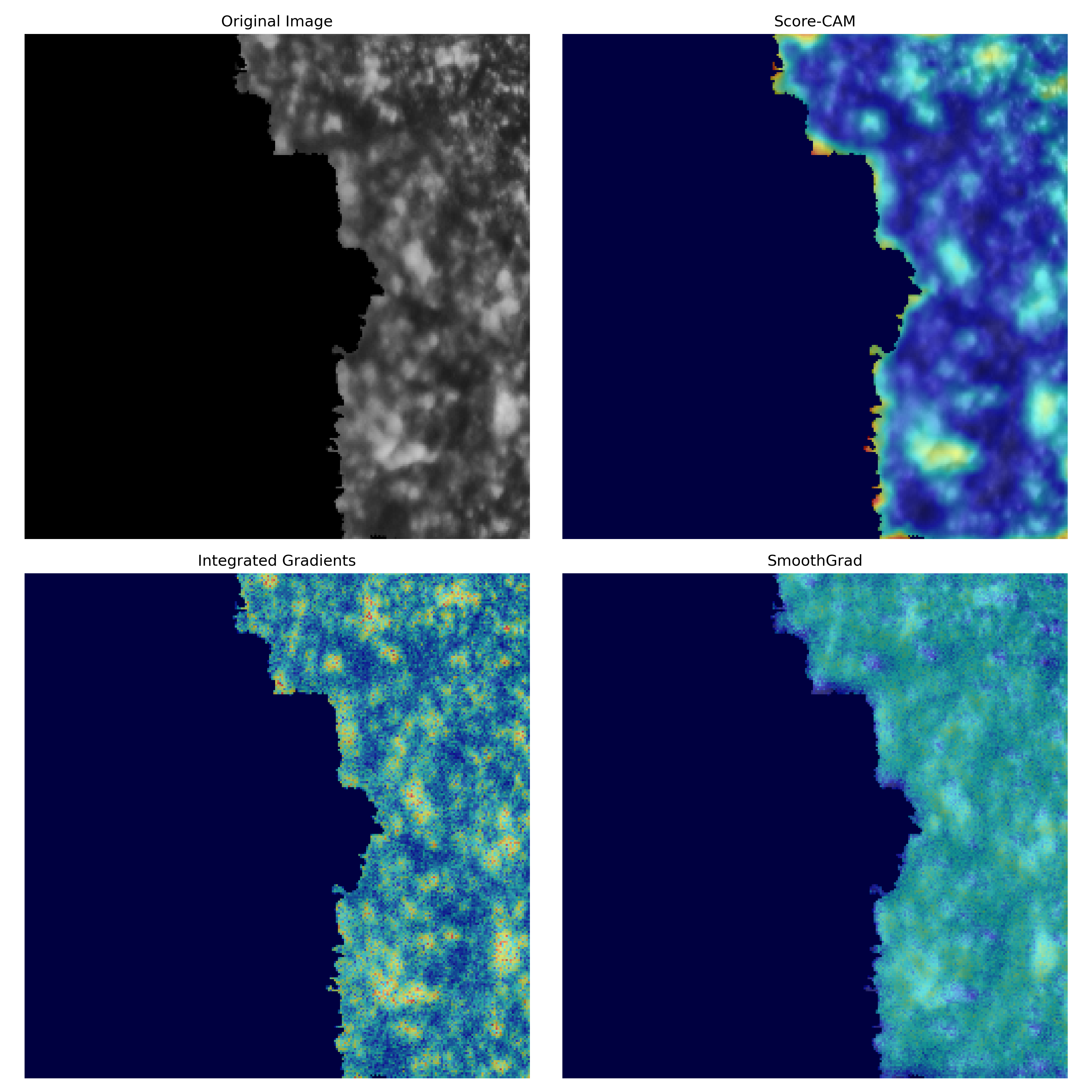

Supplement: Supplementary file 1 — Supplementary Material 1 [file 41598_2025_18179_MOESM1_ESM.tar › supplementary_material_resubmit1/Supplementary Figure S4/saliency maps/custom_CNN/100x_1000_16/bone_chichaoua_flint_SC_1000_1_area_1_area_1_x100_1_quadrant_1.tif_visualization.png]

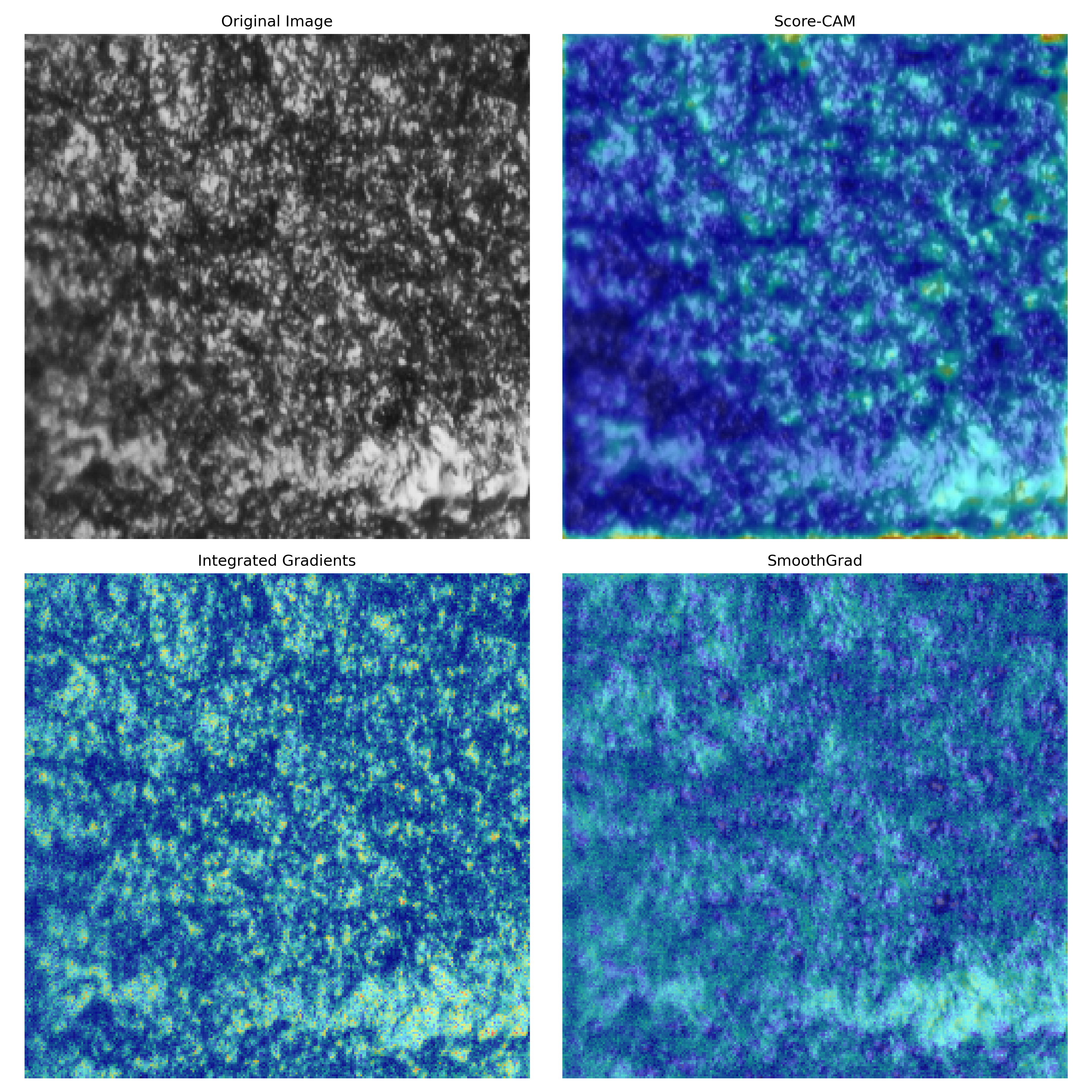

Supplement: Supplementary file 1 — Supplementary Material 1 [file 41598_2025_18179_MOESM1_ESM.tar › supplementary_material_resubmit1/Supplementary Figure S4/saliency maps/custom_CNN/100x_1000_16/bone_chichaoua_flint_SC_1000_1_area_1_area_1_x100_1_quadrant_2.tif_visualization.png]

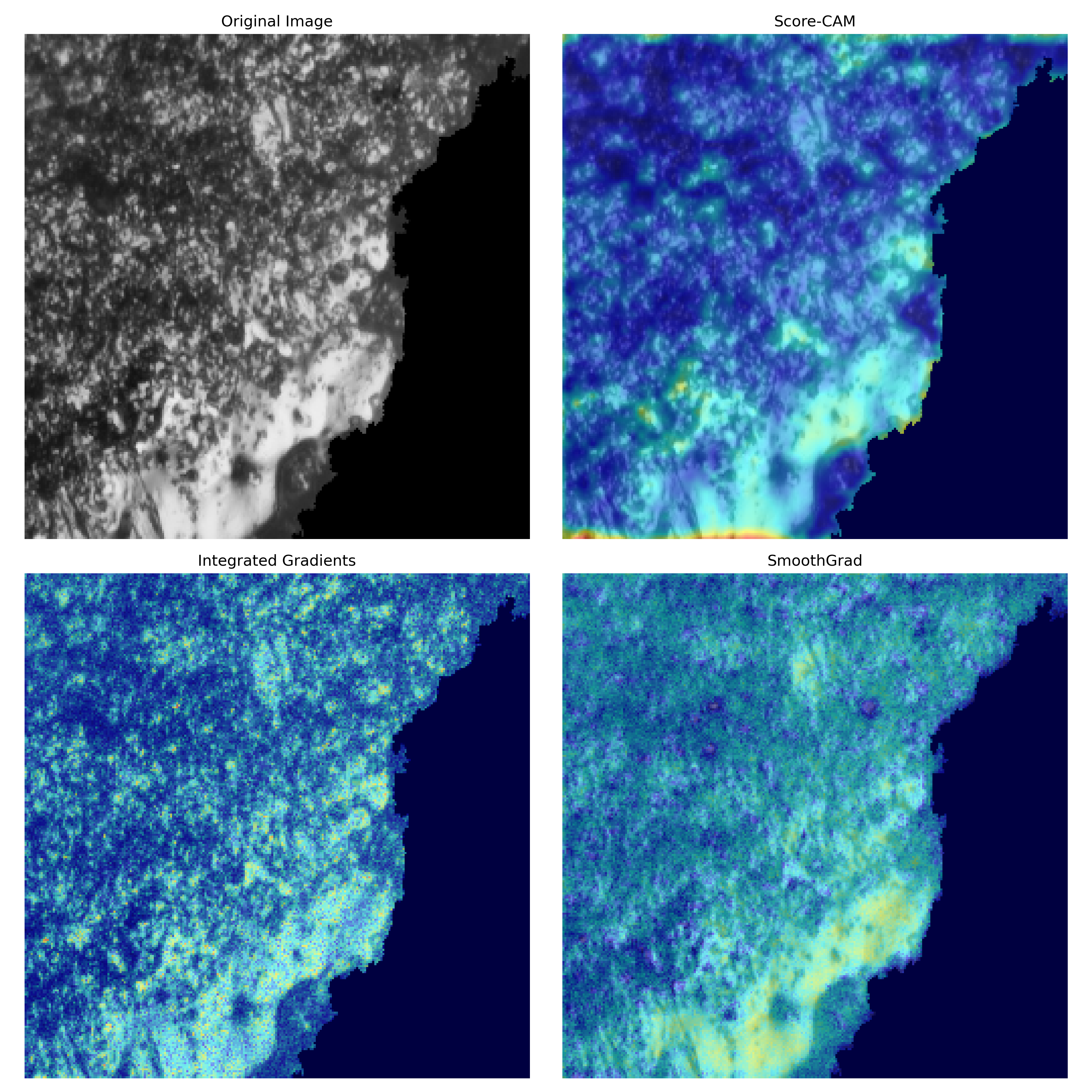

Supplement: Supplementary file 1 — Supplementary Material 1 [file 41598_2025_18179_MOESM1_ESM.tar › supplementary_material_resubmit1/Supplementary Figure S4/saliency maps/custom_CNN/100x_1000_16/bone_chichaoua_flint_SC_1000_1_area_1_area_1_x100_1_quadrant_5.tif_visualization.png]

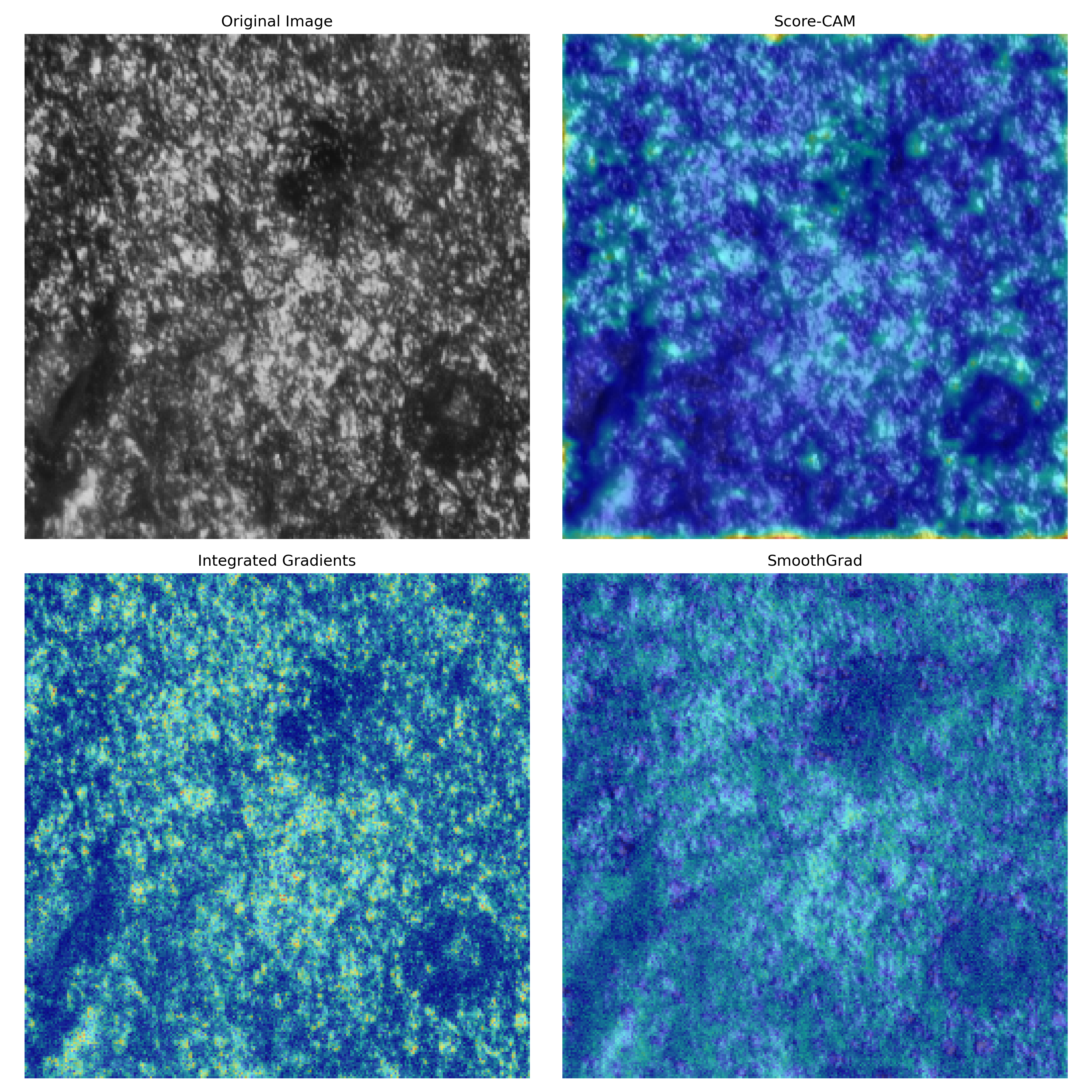

Supplement: Supplementary file 1 — Supplementary Material 1 [file 41598_2025_18179_MOESM1_ESM.tar › supplementary_material_resubmit1/Supplementary Figure S4/saliency maps/custom_CNN/100x_1000_16/bone_chichaoua_flint_SC_1000_1_area_2_area_2_x100_1_quadrant_1.tif_visualization.png]

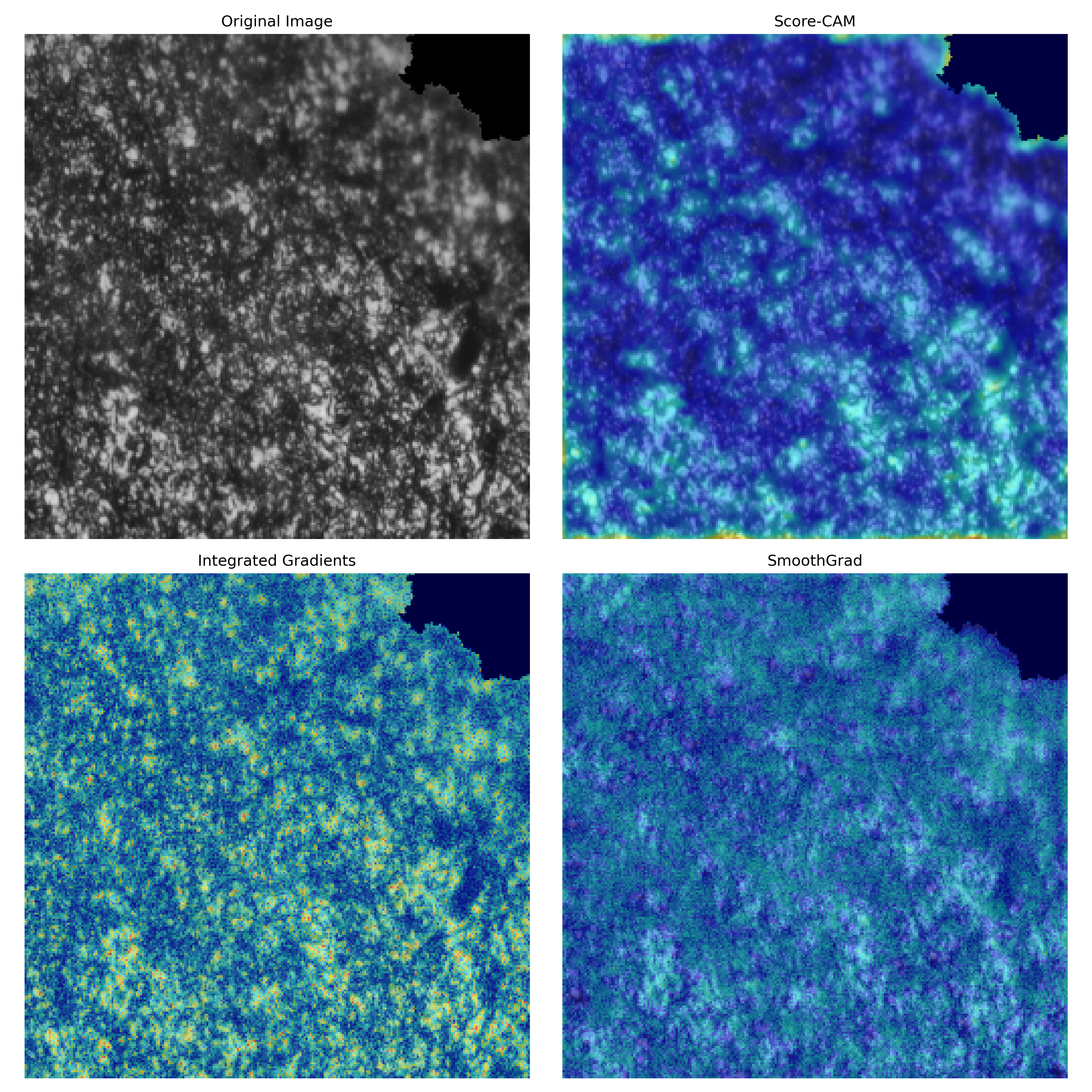

Supplement: Supplementary file 1 — Supplementary Material 1 [file 41598_2025_18179_MOESM1_ESM.tar › supplementary_material_resubmit1/Supplementary Figure S4/saliency maps/custom_CNN/100x_1000_16/bone_chichaoua_flint_SC_1000_1_area_2_area_2_x100_1_quadrant_14.tif_visualization.png]

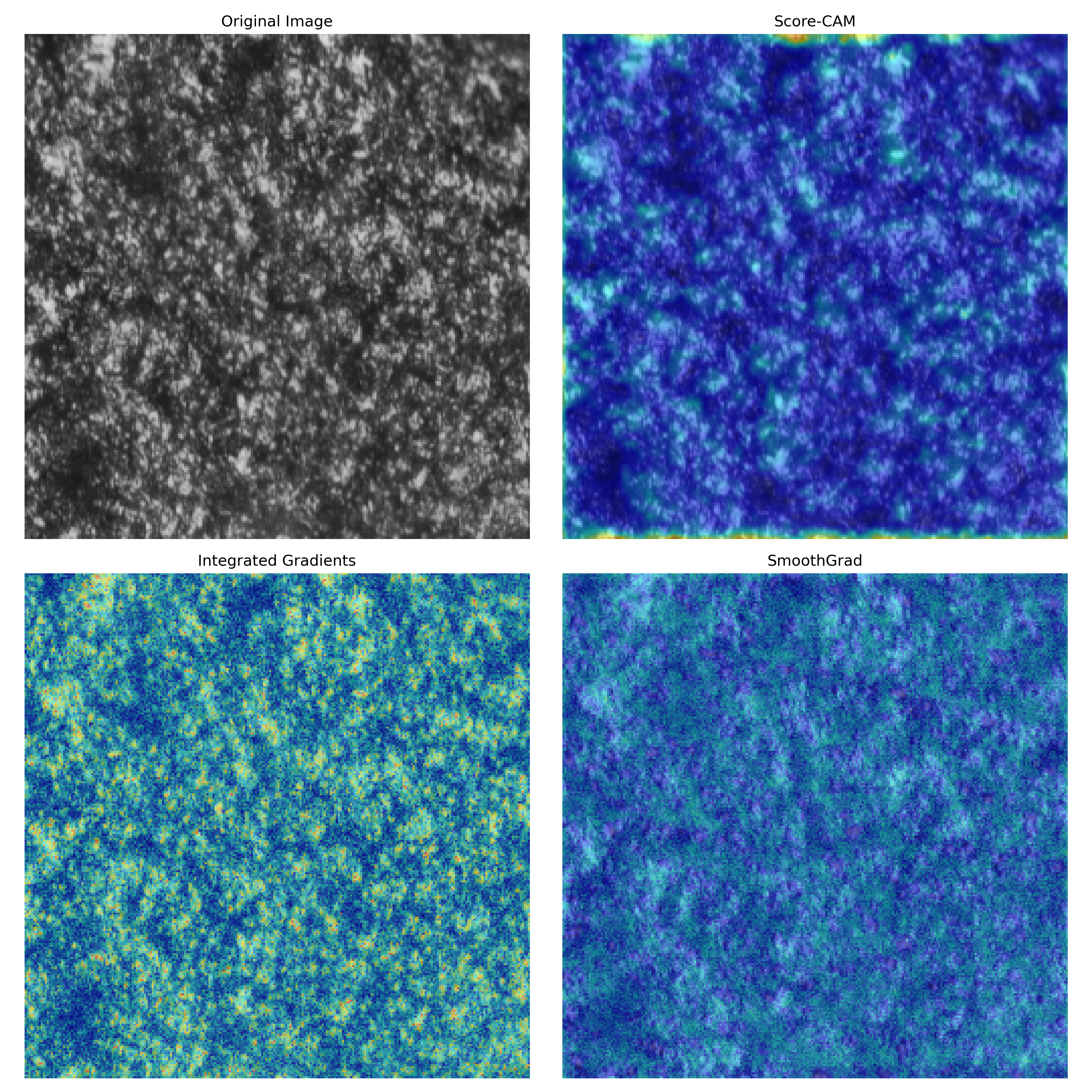

Supplement: Supplementary file 1 — Supplementary Material 1 [file 41598_2025_18179_MOESM1_ESM.tar › supplementary_material_resubmit1/Supplementary Figure S4/saliency maps/custom_CNN/100x_1000_16/bone_chichaoua_flint_SC_1000_1_area_2_area_2_x100_1_quadrant_3.tif_visualization.png]

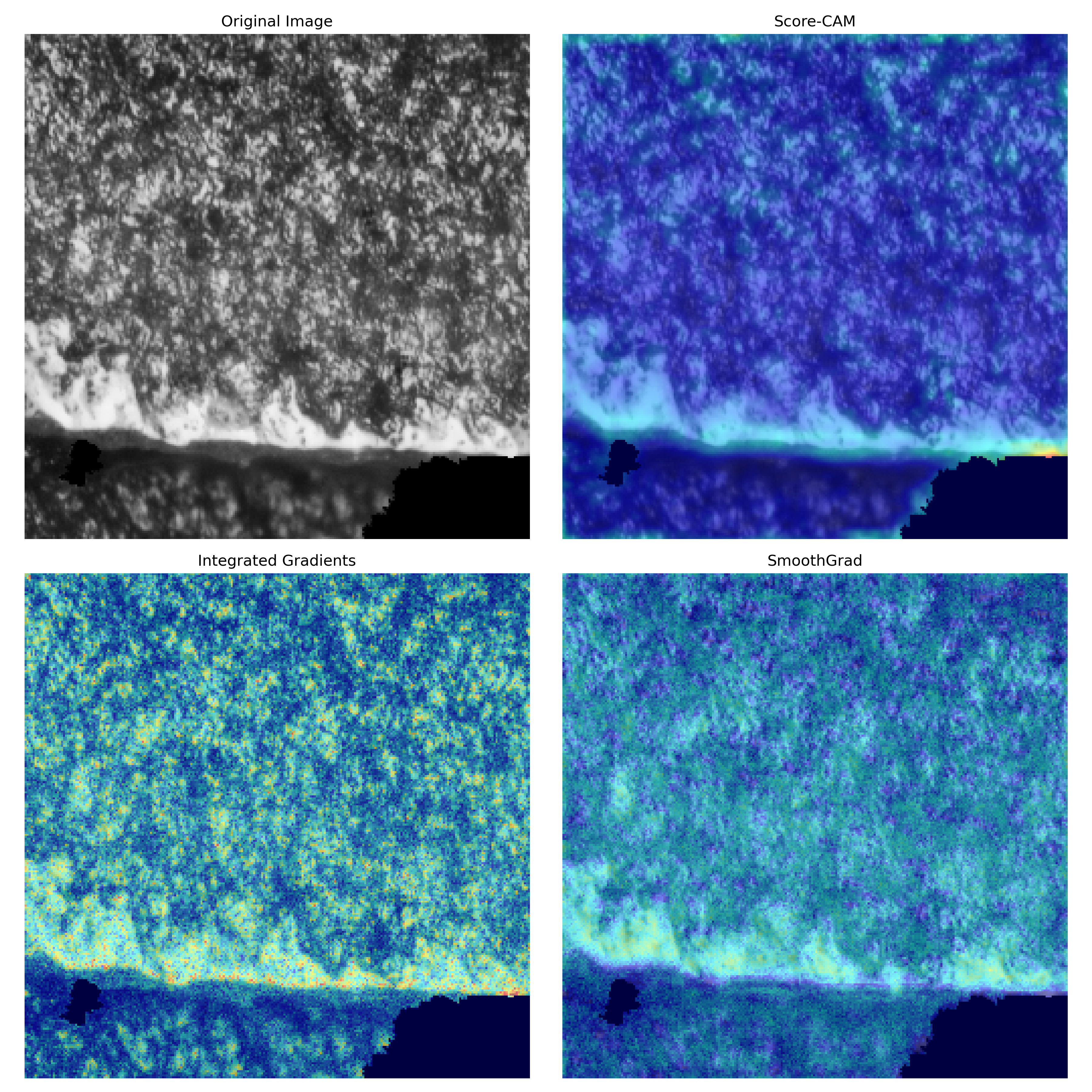

Supplement: Supplementary file 1 — Supplementary Material 1 [file 41598_2025_18179_MOESM1_ESM.tar › supplementary_material_resubmit1/Supplementary Figure S4/saliency maps/custom_CNN/100x_1000_16/bone_chichaoua_flint_SC_1000_1_area_2_area_2_x100_1_quadrant_6.tif_visualization.png]

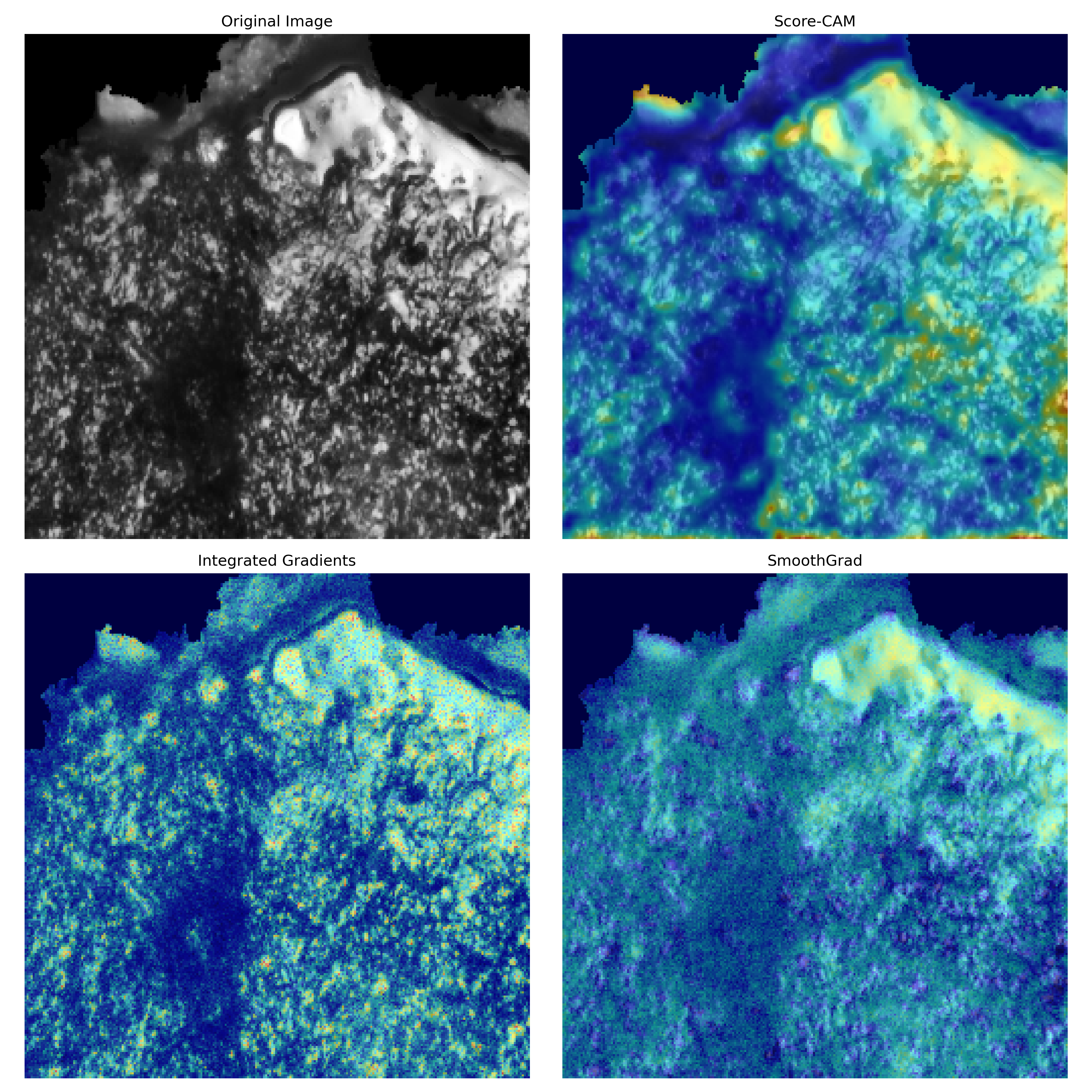

Supplement: Supplementary file 1 — Supplementary Material 1 [file 41598_2025_18179_MOESM1_ESM.tar › supplementary_material_resubmit1/Supplementary Figure S4/saliency maps/custom_CNN/100x_1000_16/bone_chichaoua_flint_SC_1000_1_area_3_area_2_x100_1_quadrant_1.tif_visualization.png]

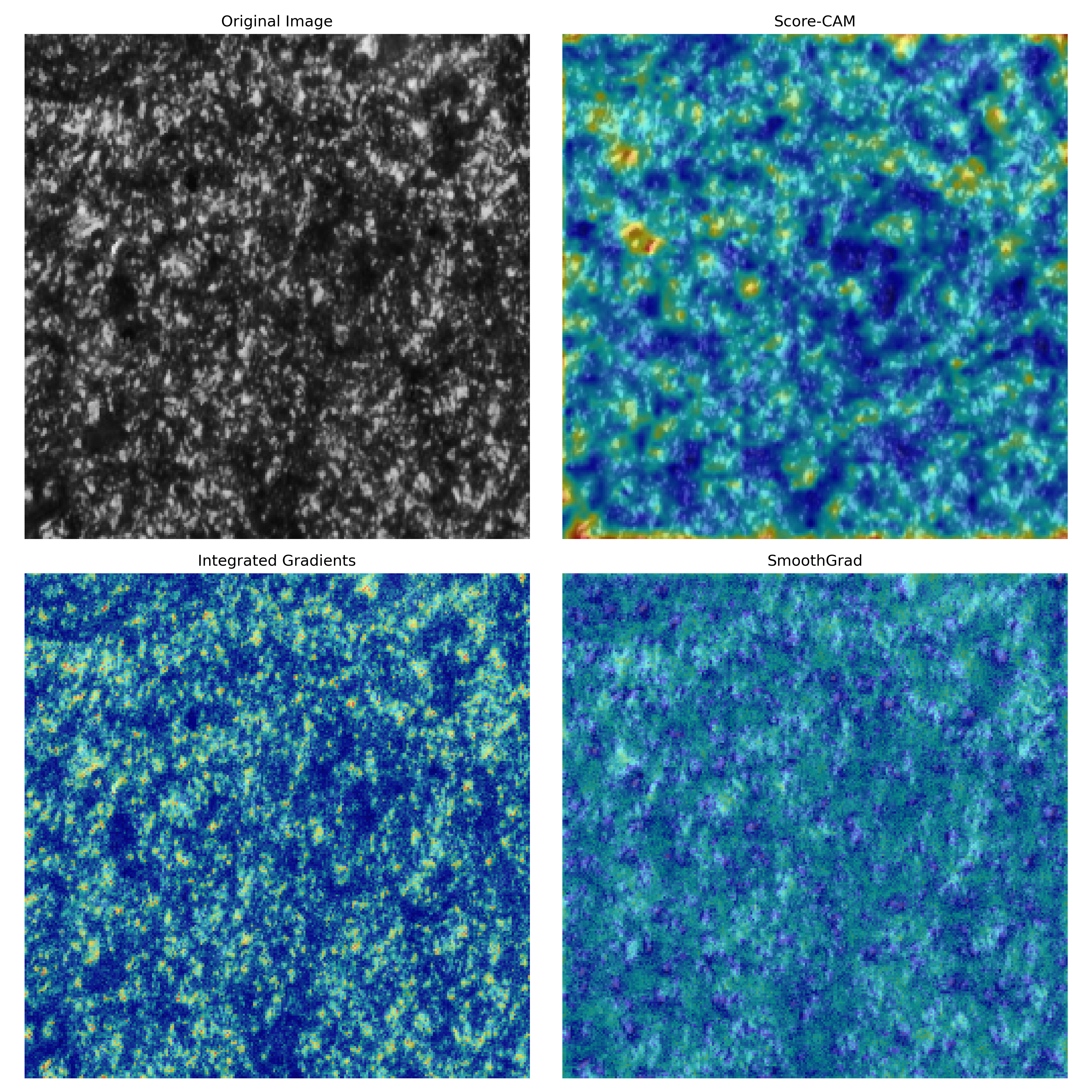

Supplement: Supplementary file 1 — Supplementary Material 1 [file 41598_2025_18179_MOESM1_ESM.tar › supplementary_material_resubmit1/Supplementary Figure S4/saliency maps/custom_CNN/100x_1000_16/bone_chichaoua_flint_SC_1000_1_area_3_area_2_x100_1_quadrant_3.tif_visualization.png]

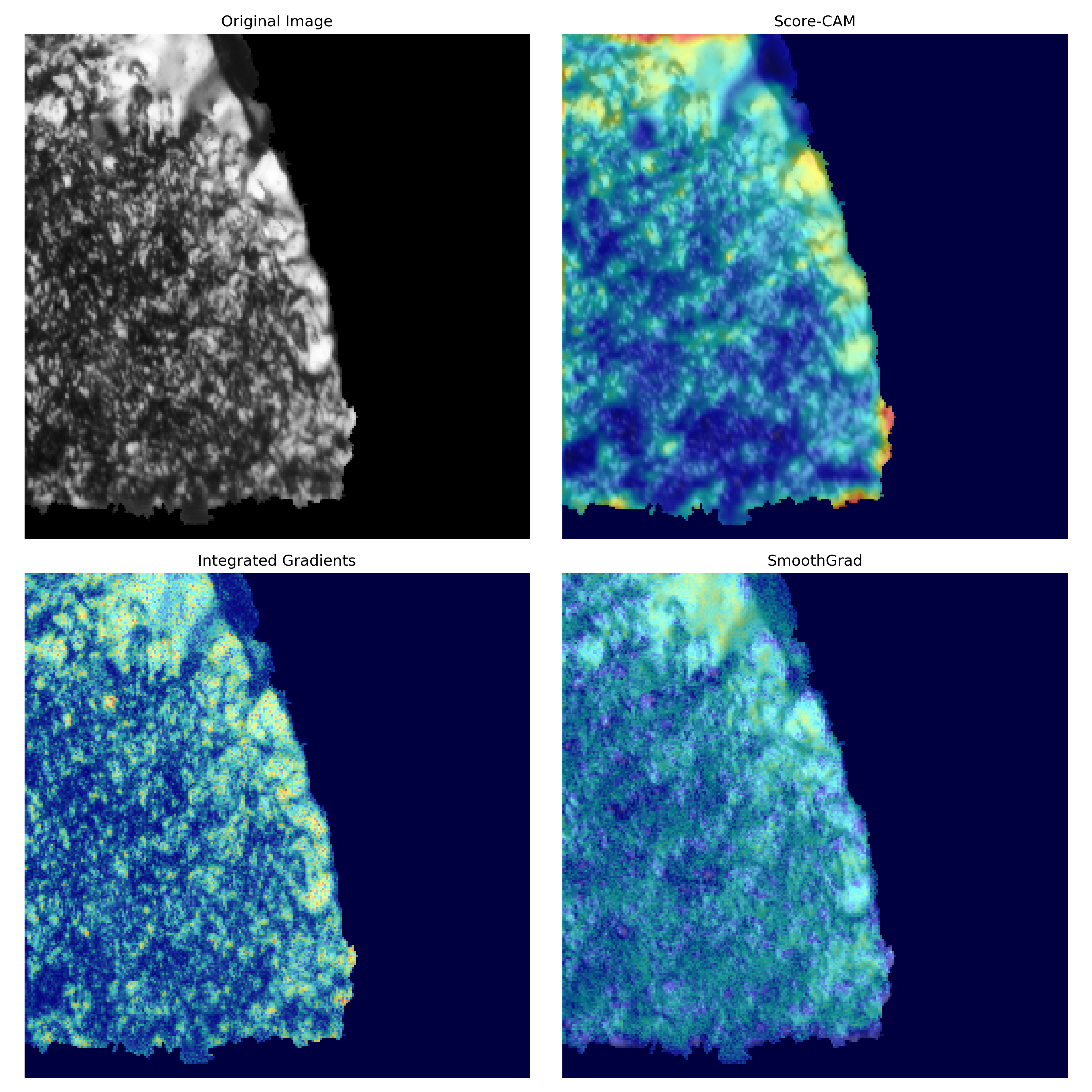

Supplement: Supplementary file 1 — Supplementary Material 1 [file 41598_2025_18179_MOESM1_ESM.tar › supplementary_material_resubmit1/Supplementary Figure S4/saliency maps/custom_CNN/100x_1000_16/bone_chichaoua_flint_SC_1000_1_area_3_area_2_x100_1_quadrant_9.tif_visualization.png]

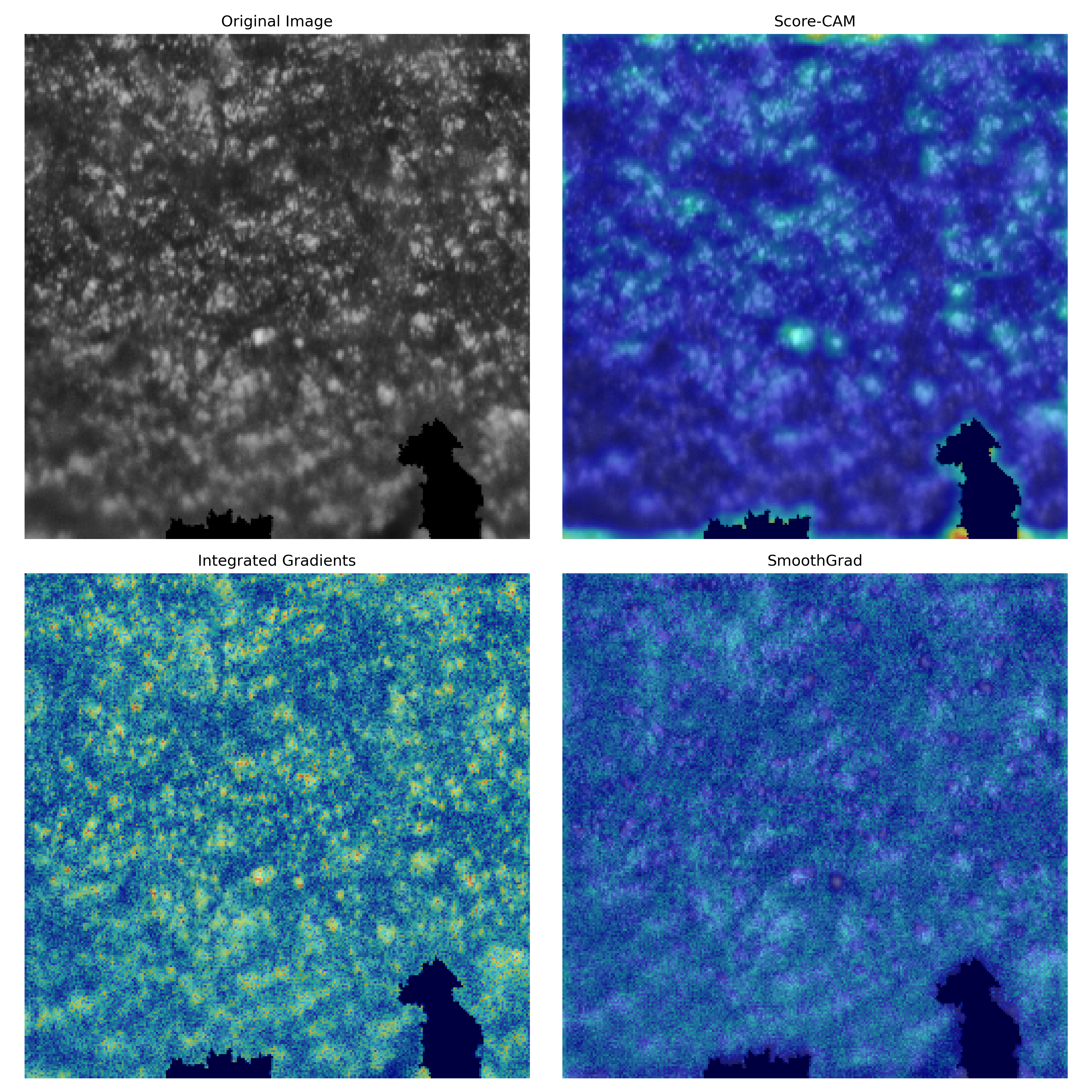

Supplement: Supplementary file 1 — Supplementary Material 1 [file 41598_2025_18179_MOESM1_ESM.tar › supplementary_material_resubmit1/Supplementary Figure S4/saliency maps/custom_CNN/100x_1000_16/wood_SC_600_area_1_area_1_x100_1_quadrant_7.tif_visualization.png]

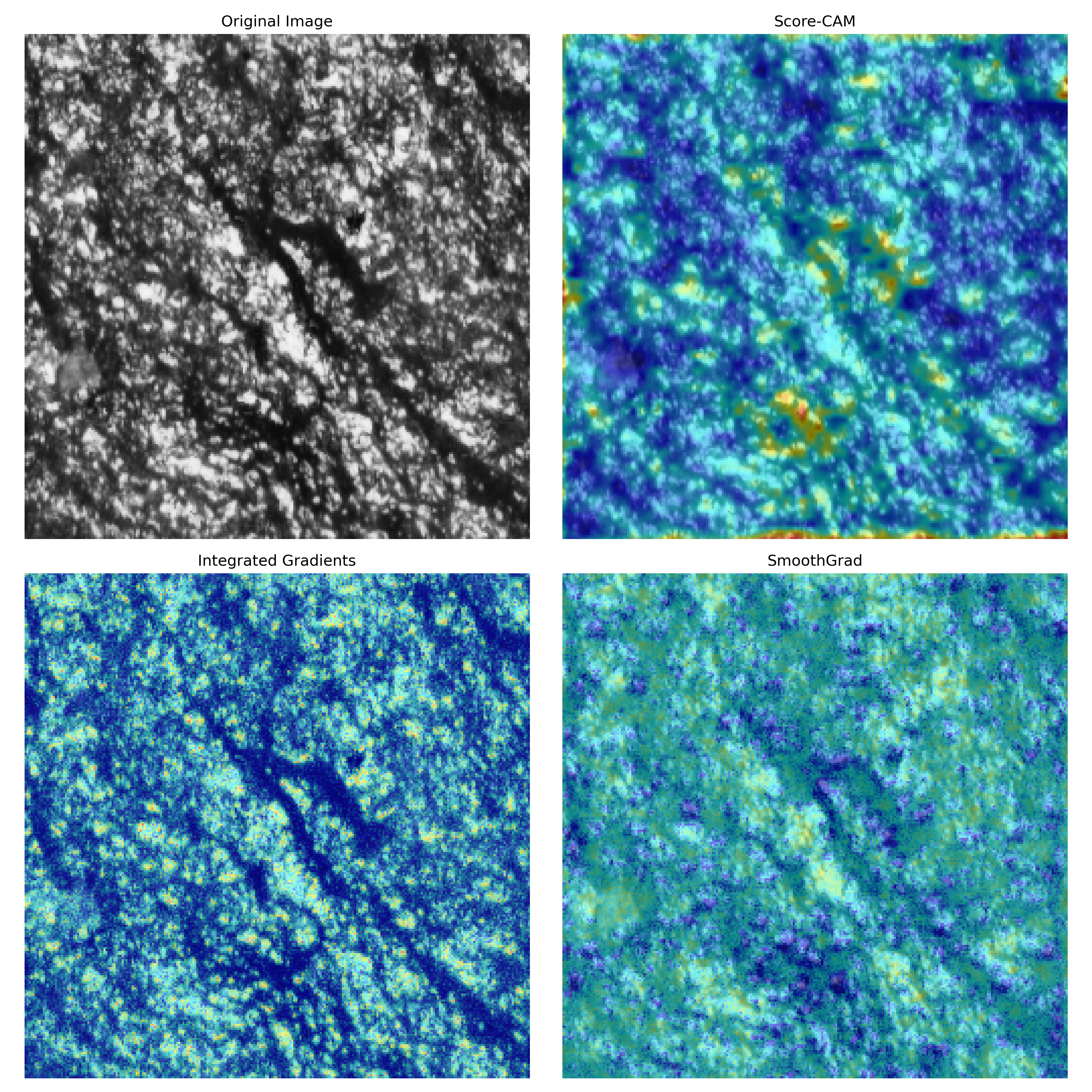

Supplement: Supplementary file 1 — Supplementary Material 1 [file 41598_2025_18179_MOESM1_ESM.tar › supplementary_material_resubmit1/Supplementary Figure S4/saliency maps/custom_CNN/100x_1000_16/wood_SC_900_area_1_area_1_x100_1_quadrant_10.tif_visualization.png]

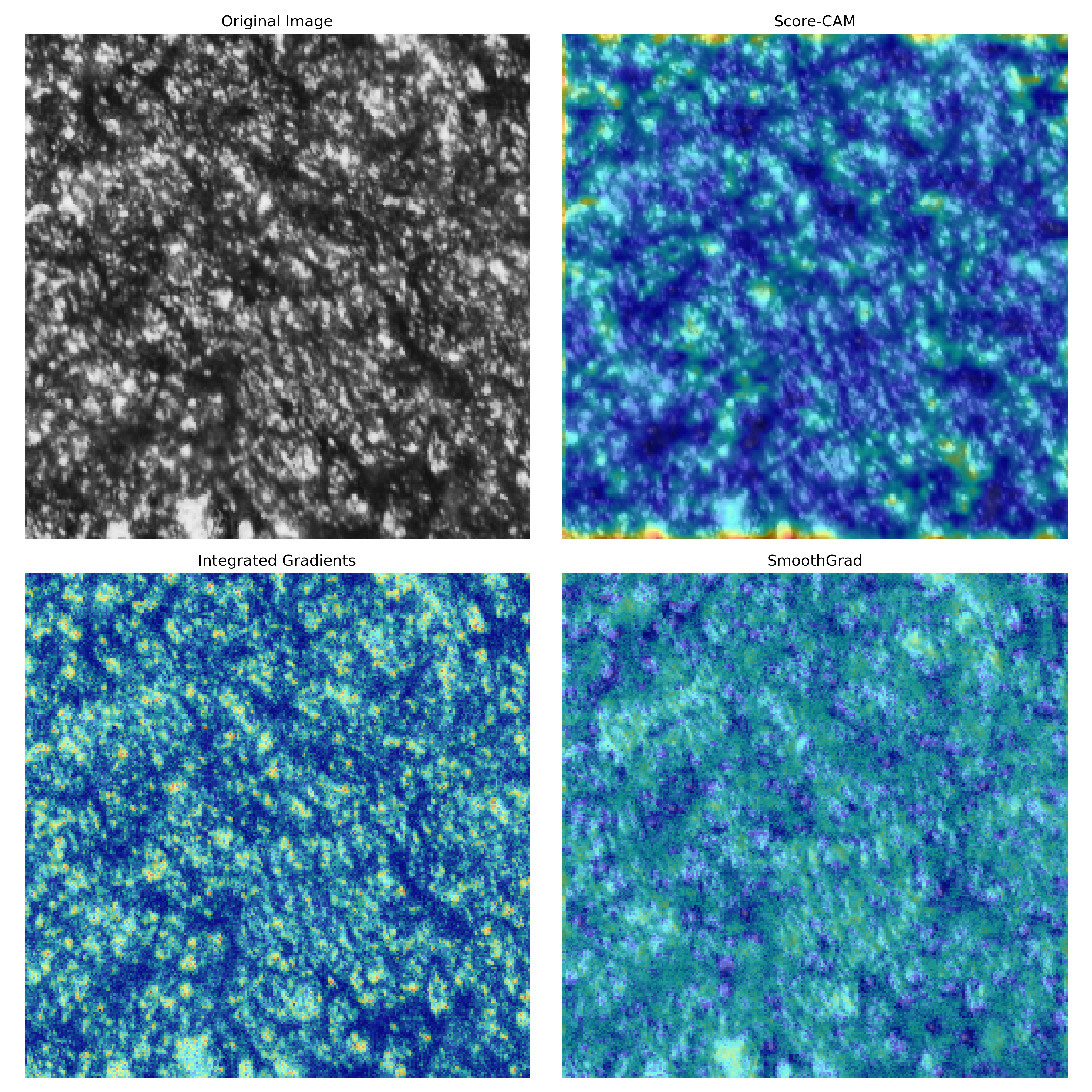

Supplement: Supplementary file 1 — Supplementary Material 1 [file 41598_2025_18179_MOESM1_ESM.tar › supplementary_material_resubmit1/Supplementary Figure S4/saliency maps/custom_CNN/100x_1000_16/wood_SC_900_area_1_area_1_x100_1_quadrant_12.tif_visualization.png]

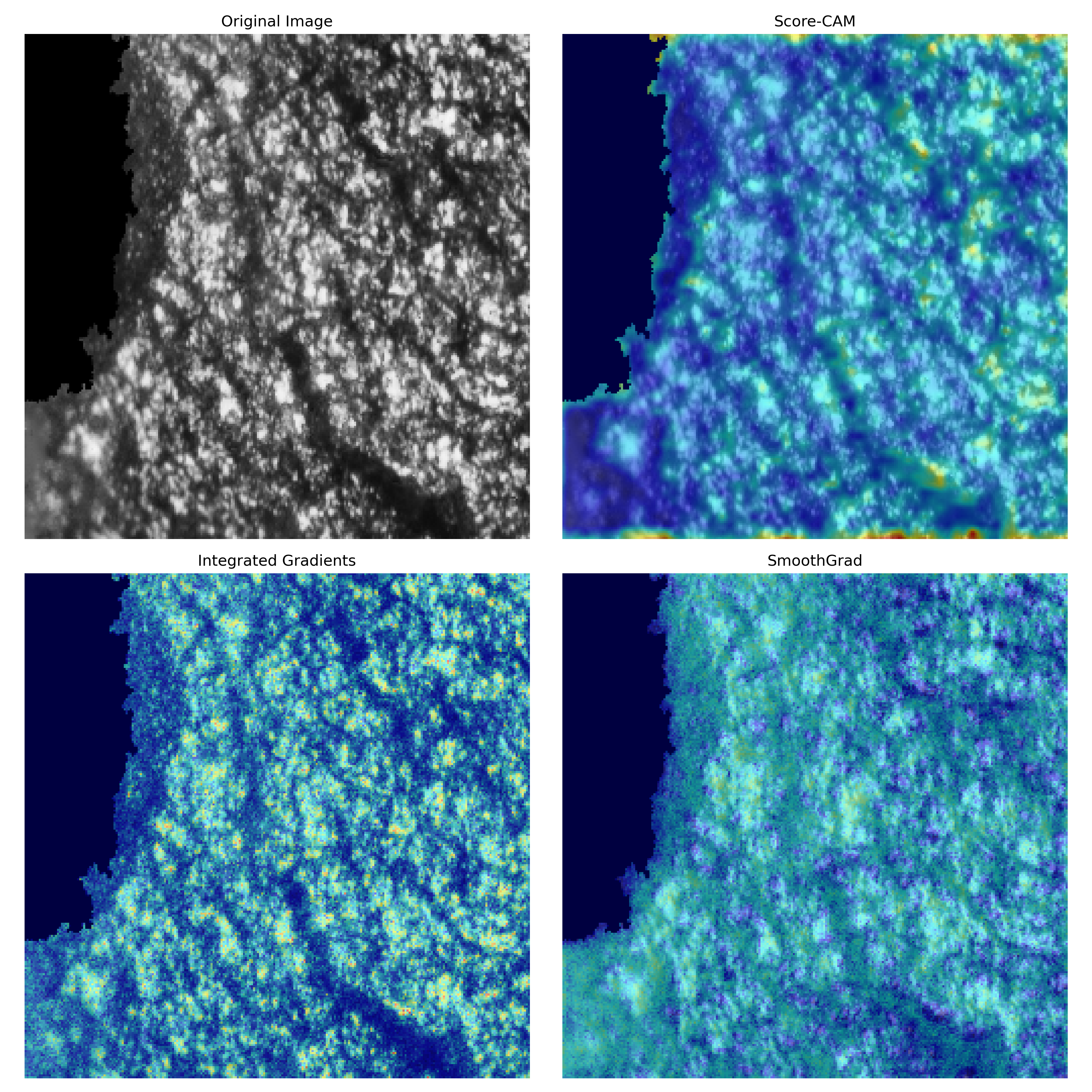

Supplement: Supplementary file 1 — Supplementary Material 1 [file 41598_2025_18179_MOESM1_ESM.tar › supplementary_material_resubmit1/Supplementary Figure S4/saliency maps/custom_CNN/100x_1000_16/wood_SC_900_area_1_area_1_x100_1_quadrant_9.tif_visualization.png]

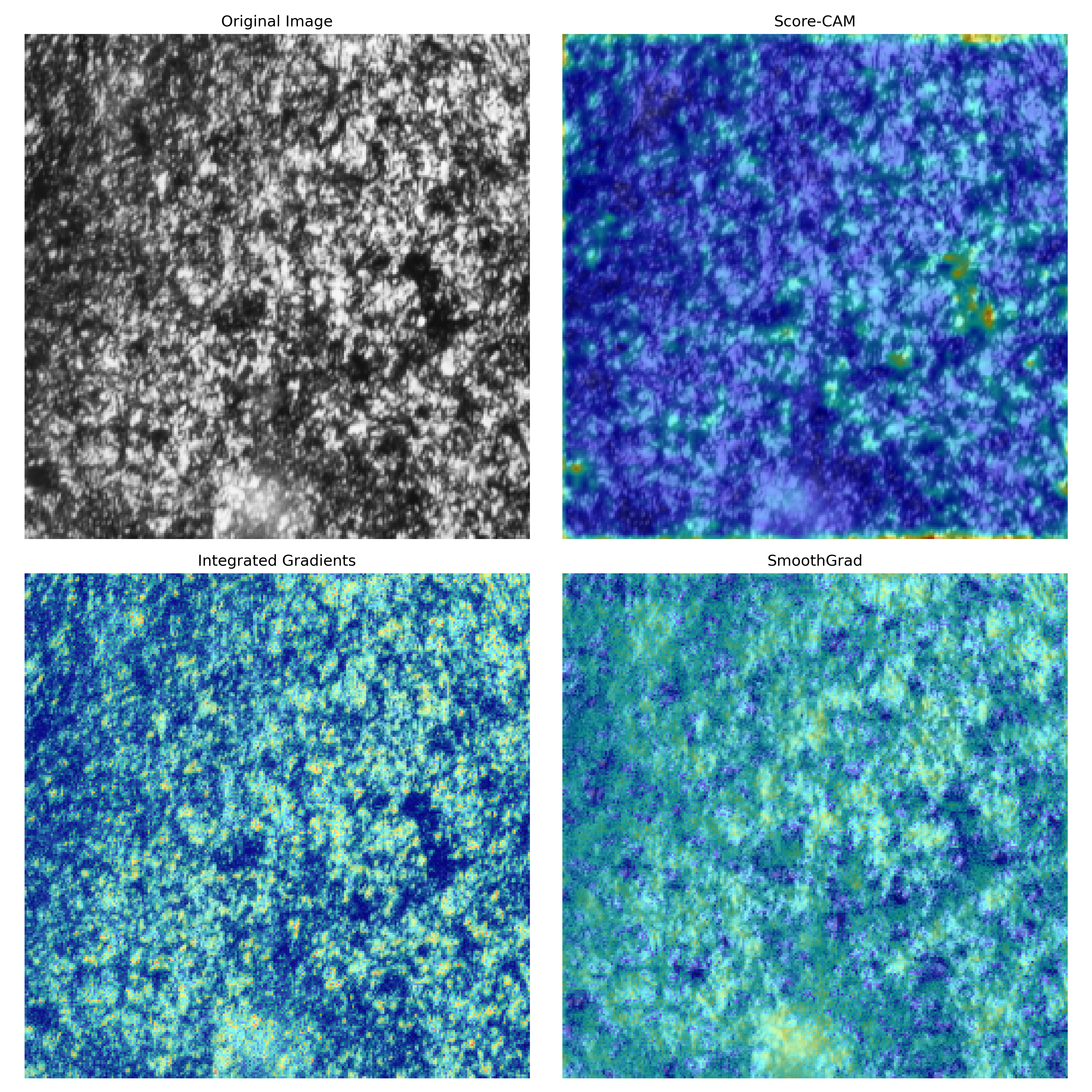

Supplement: Supplementary file 1 — Supplementary Material 1 [file 41598_2025_18179_MOESM1_ESM.tar › supplementary_material_resubmit1/Supplementary Figure S4/saliency maps/custom_CNN/100x_1000_16/wood_SC_900_area_2_area_2_x100_1_quadrant_6.tif_visualization.png]

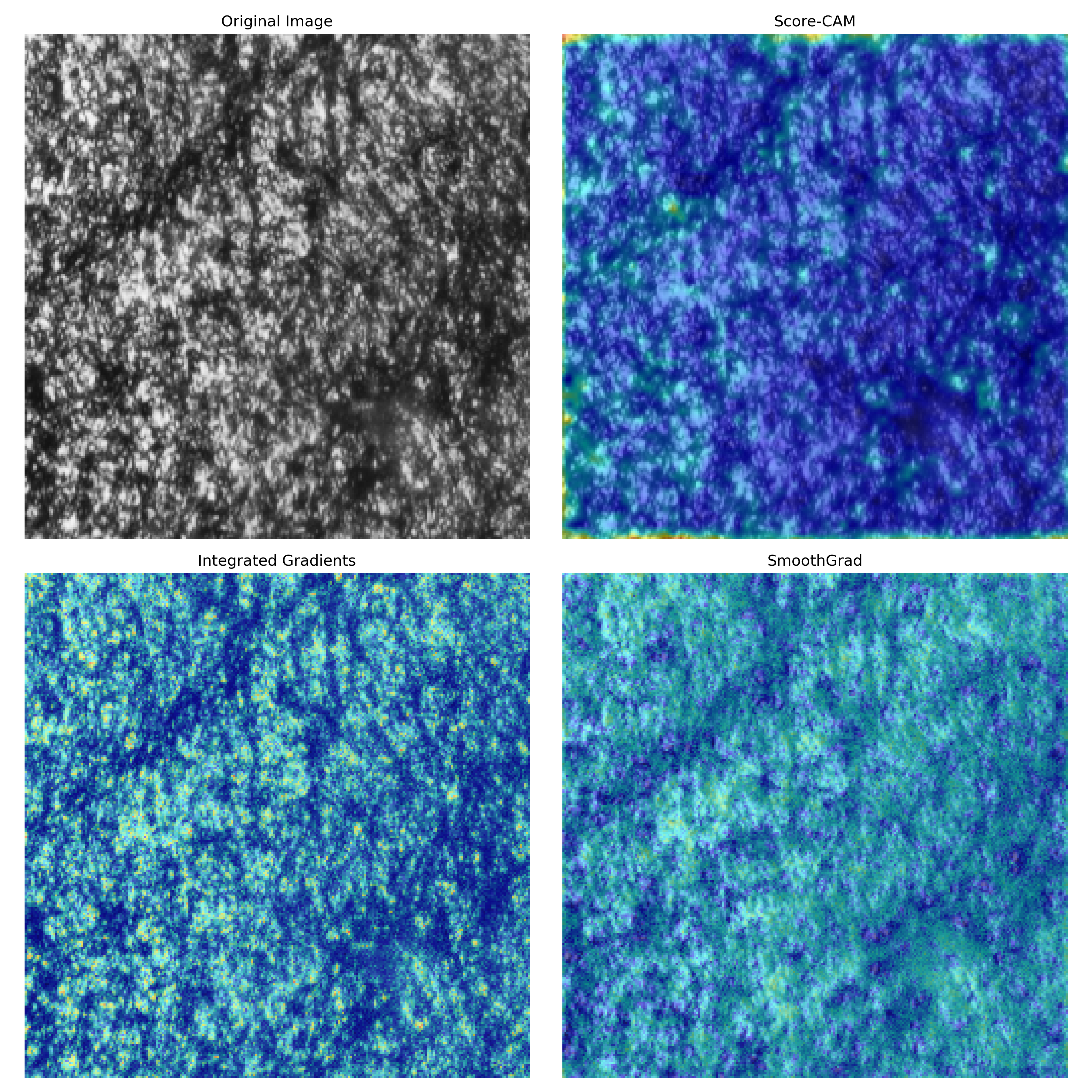

Supplement: Supplementary file 1 — Supplementary Material 1 [file 41598_2025_18179_MOESM1_ESM.tar › supplementary_material_resubmit1/Supplementary Figure S4/saliency maps/custom_CNN/100x_1000_16/wood_SC_900_area_2_area_2_x100_1_quadrant_7.tif_visualization.png]

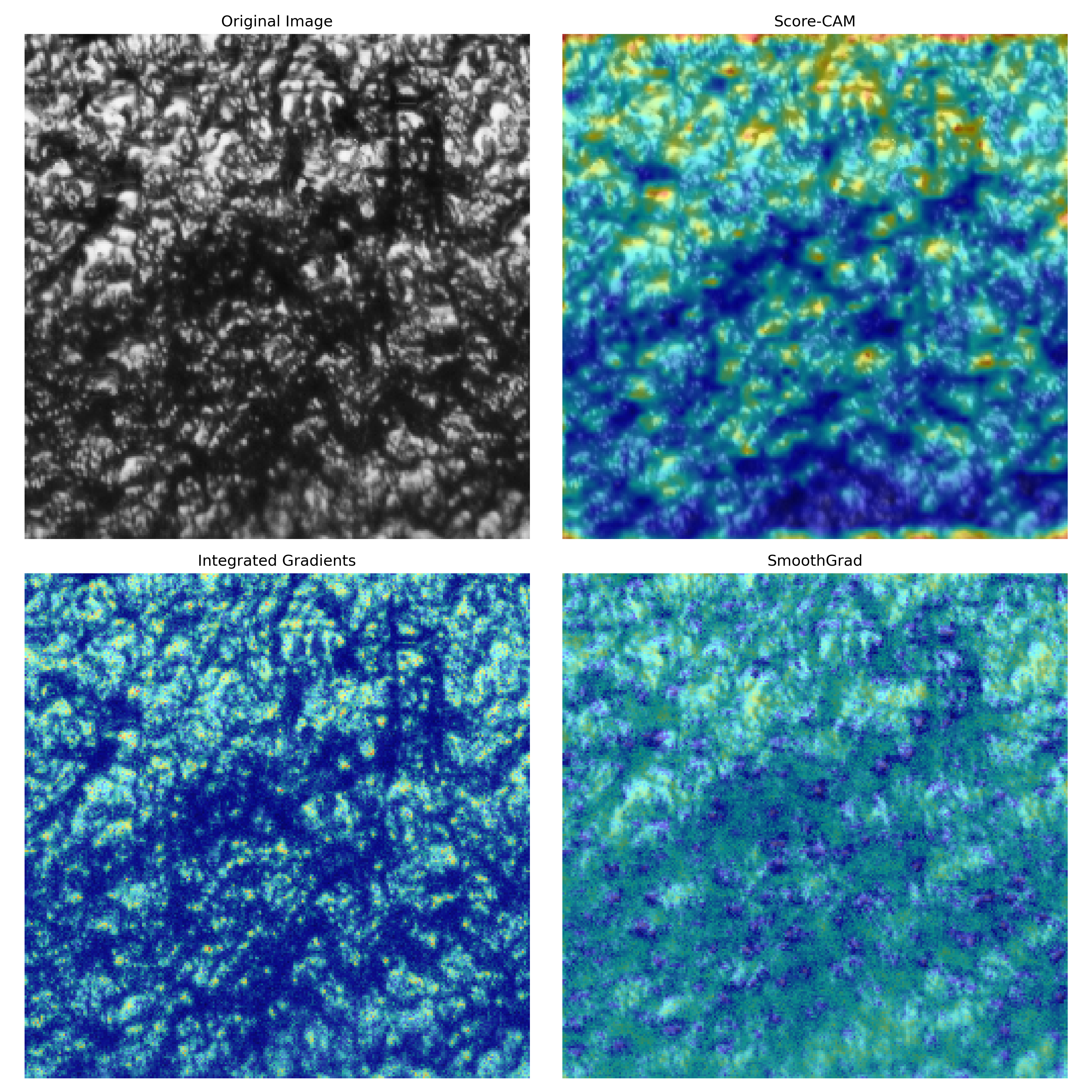

Supplement: Supplementary file 1 — Supplementary Material 1 [file 41598_2025_18179_MOESM1_ESM.tar › supplementary_material_resubmit1/Supplementary Figure S4/saliency maps/custom_CNN/100x_1000_16/wood_SW_1000_1_area_1_area_1_x100_1_quadrant_13.tif_visualization.png]

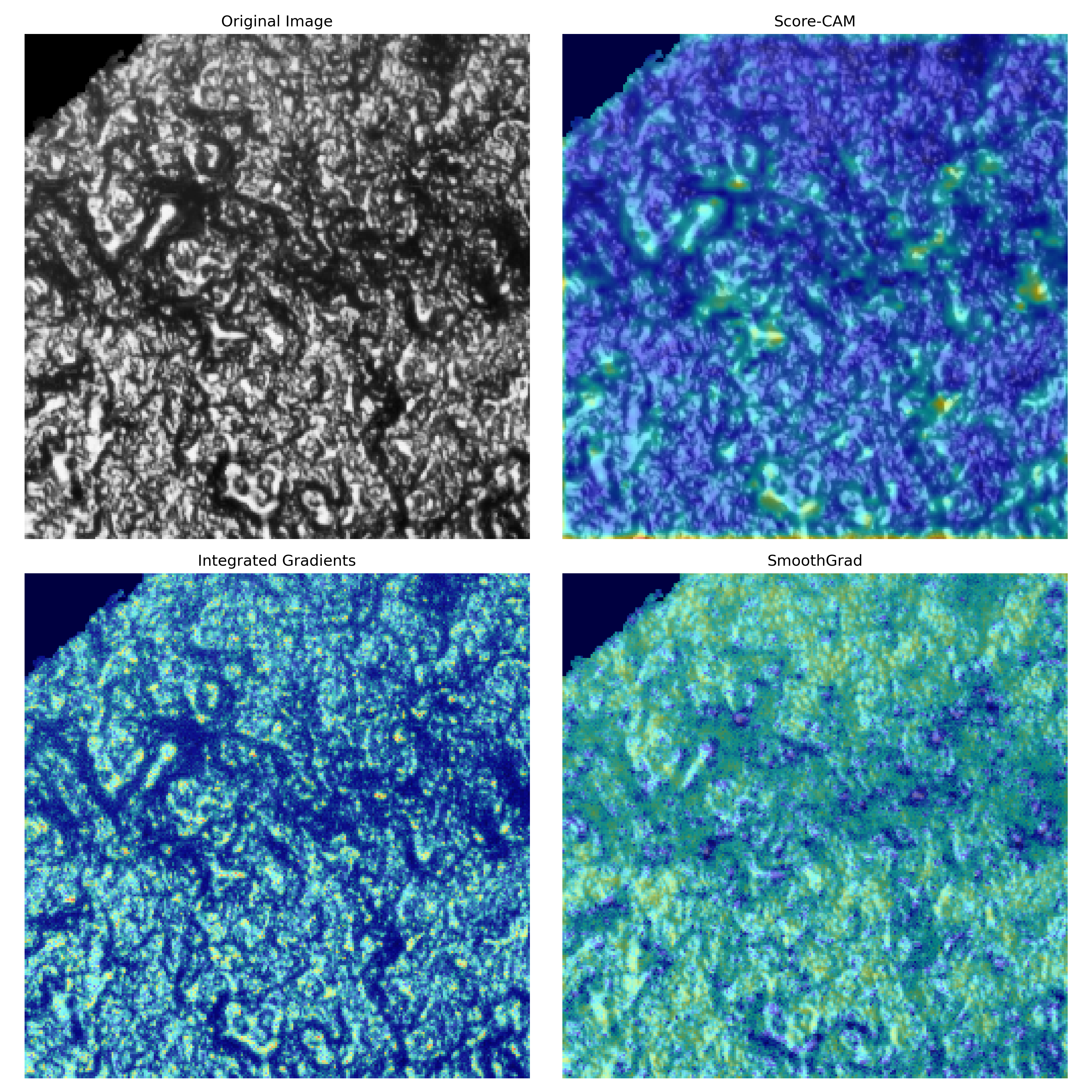

Supplement: Supplementary file 1 — Supplementary Material 1 [file 41598_2025_18179_MOESM1_ESM.tar › supplementary_material_resubmit1/Supplementary Figure S4/saliency maps/custom_CNN/100x_1000_16/wood_SW_1000_1_area_1_area_1_x100_1_quadrant_4.tif_visualization.png]

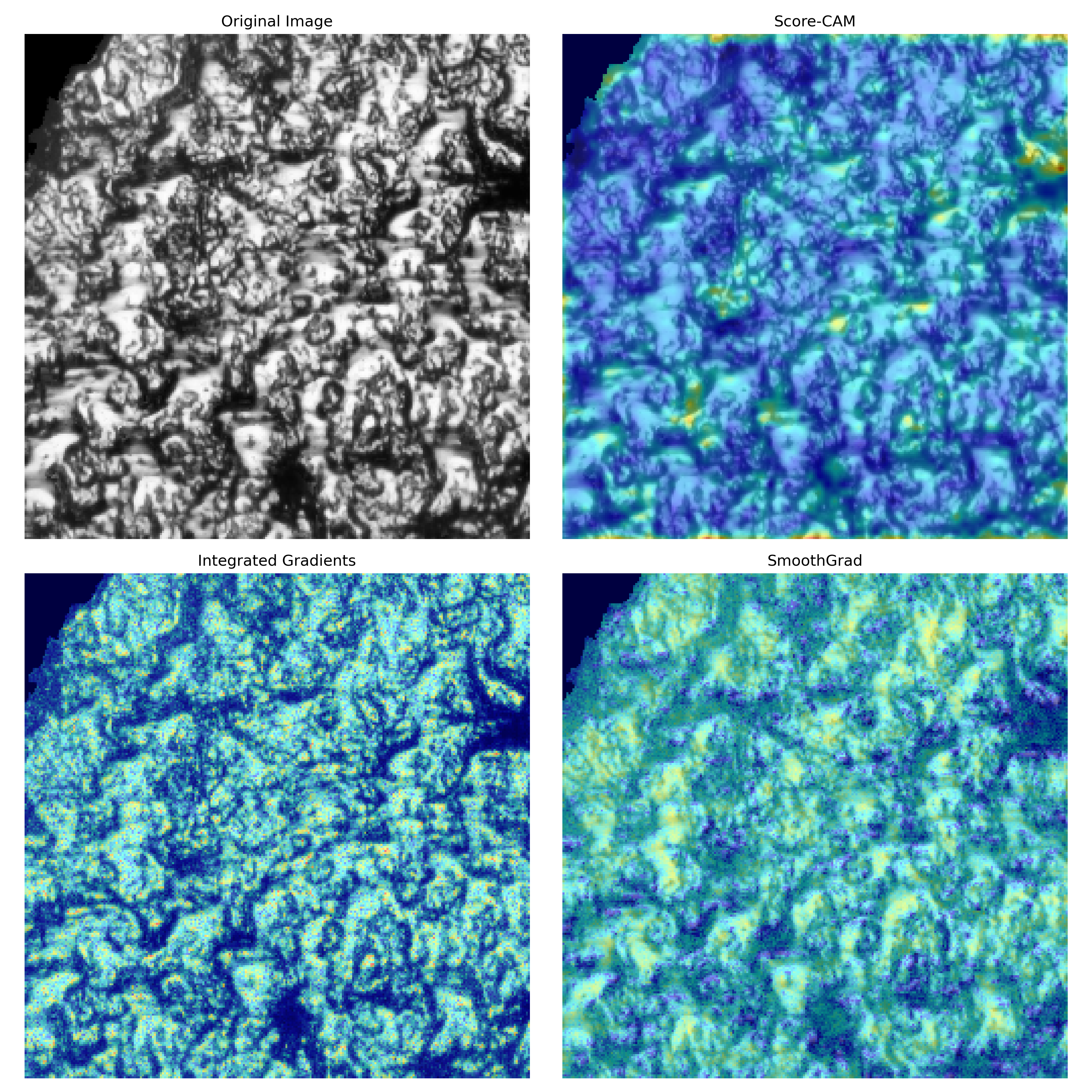

Supplement: Supplementary file 1 — Supplementary Material 1 [file 41598_2025_18179_MOESM1_ESM.tar › supplementary_material_resubmit1/Supplementary Figure S4/saliency maps/custom_CNN/100x_1000_16/wood_SW_1000_1_area_1_area_1_x100_1_quadrant_7.tif_visualization.png]

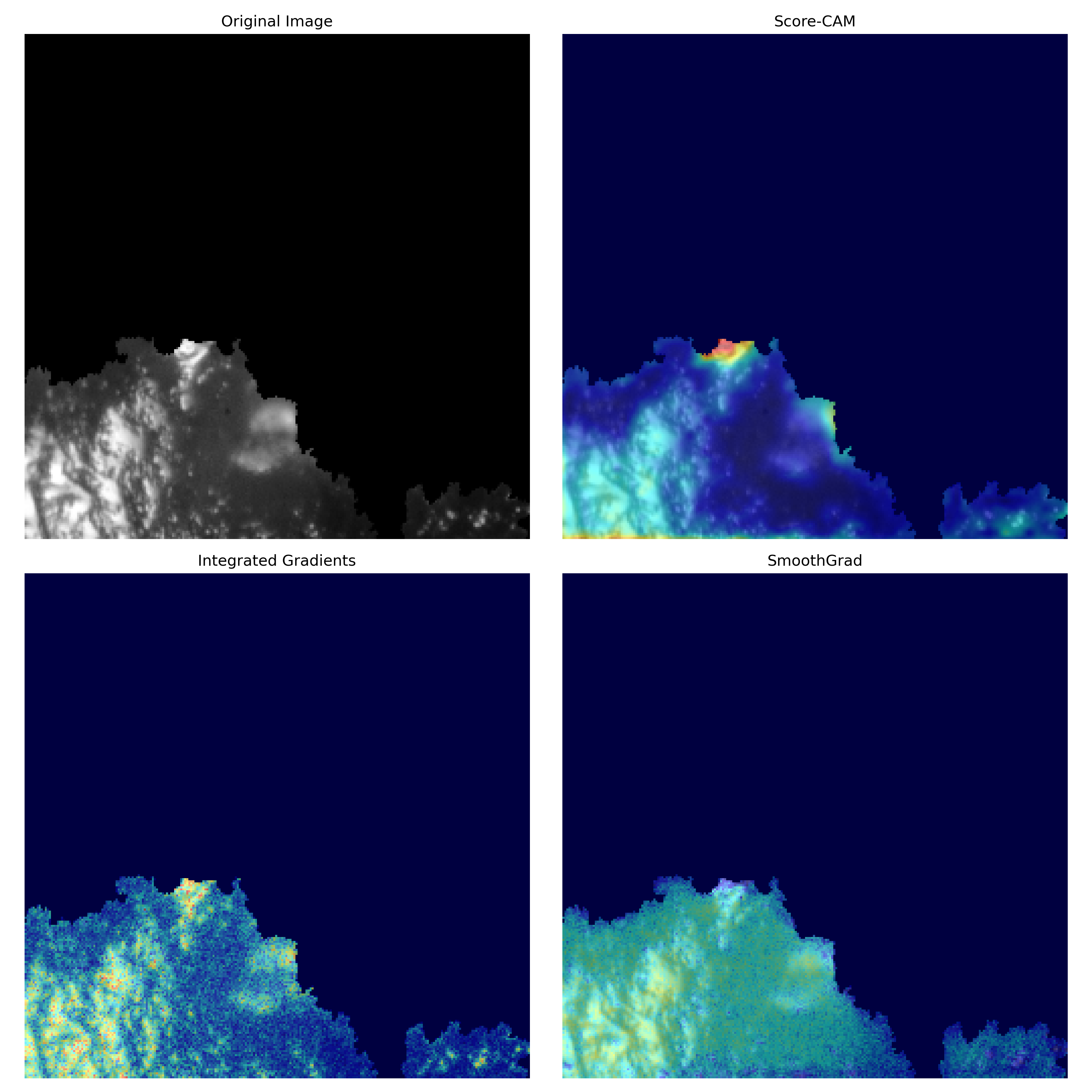

Supplement: Supplementary file 1 — Supplementary Material 1 [file 41598_2025_18179_MOESM1_ESM.tar › supplementary_material_resubmit1/Supplementary Figure S4/saliency maps/custom_CNN/100x_1000_16/wood_SW_1000_1_area_3_area_1_x100_1_quadrant_1.tif_visualization.png]

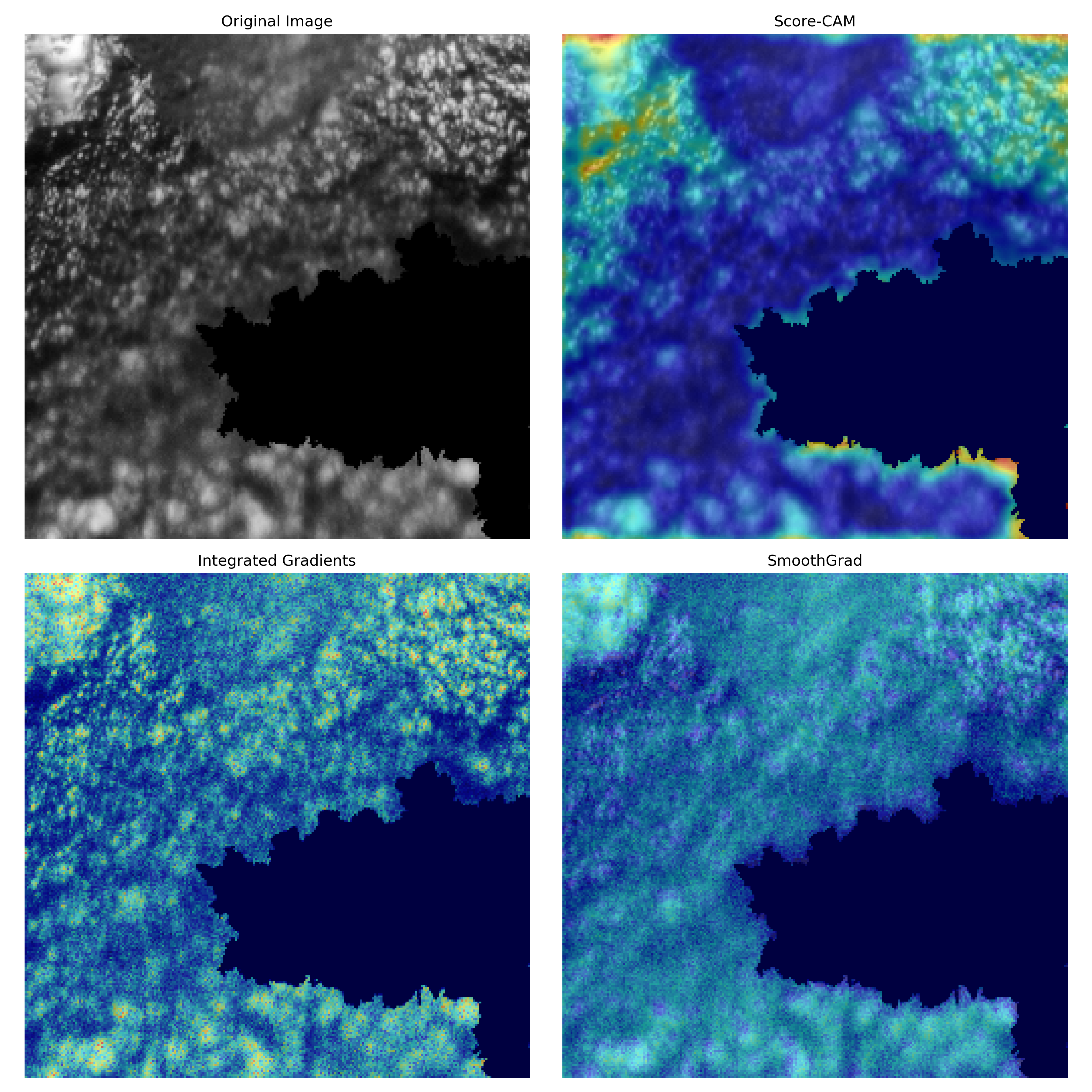

Supplement: Supplementary file 1 — Supplementary Material 1 [file 41598_2025_18179_MOESM1_ESM.tar › supplementary_material_resubmit1/Supplementary Figure S4/saliency maps/custom_CNN/100x_1000_16/wood_SW_1000_1_area_3_area_1_x100_1_quadrant_2.tif_visualization.png]

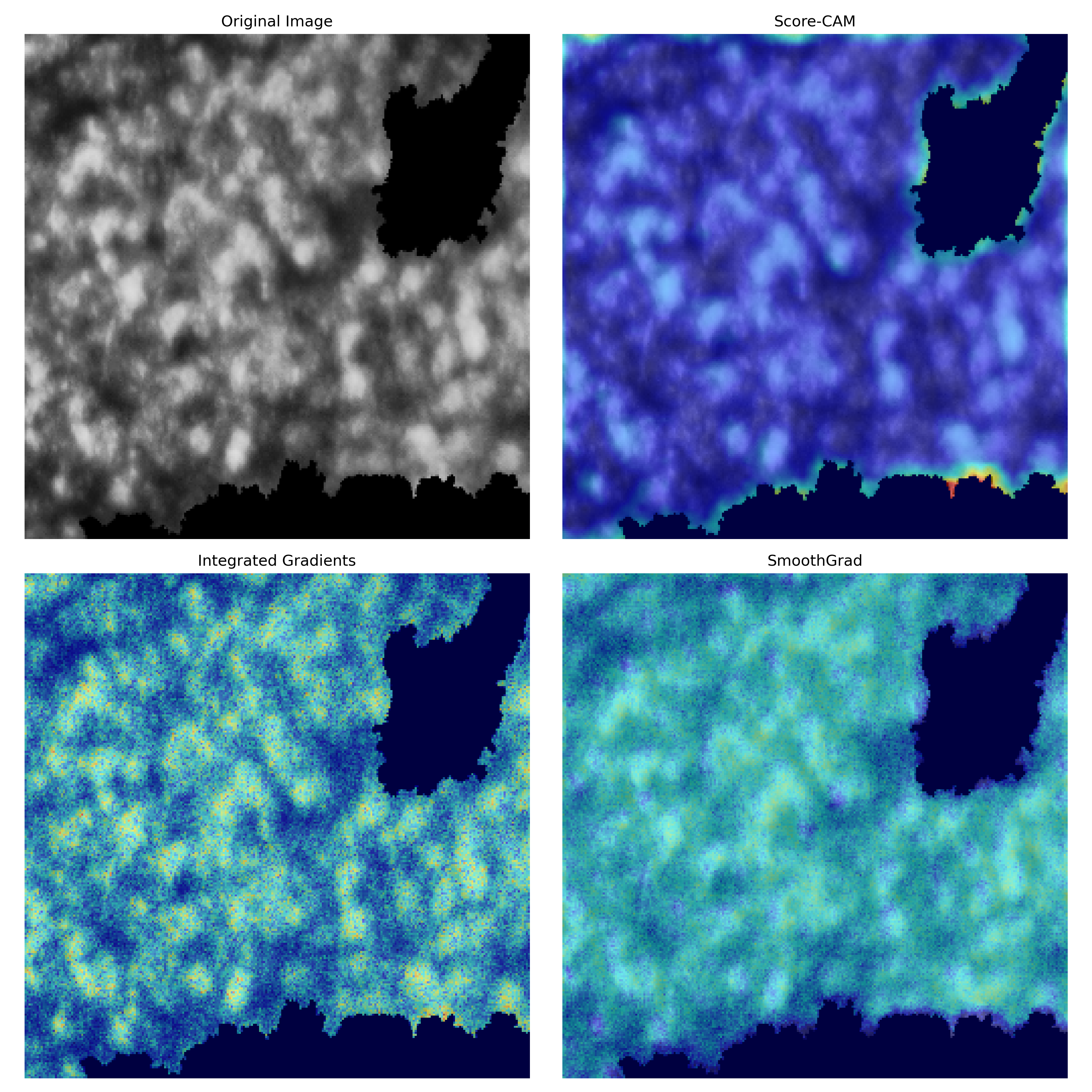

Supplement: Supplementary file 1 — Supplementary Material 1 [file 41598_2025_18179_MOESM1_ESM.tar › supplementary_material_resubmit1/Supplementary Figure S4/saliency maps/custom_CNN/100x_1000_16/wood_SW_1000_1_area_3_area_1_x100_1_quadrant_5.tif_visualization.png]

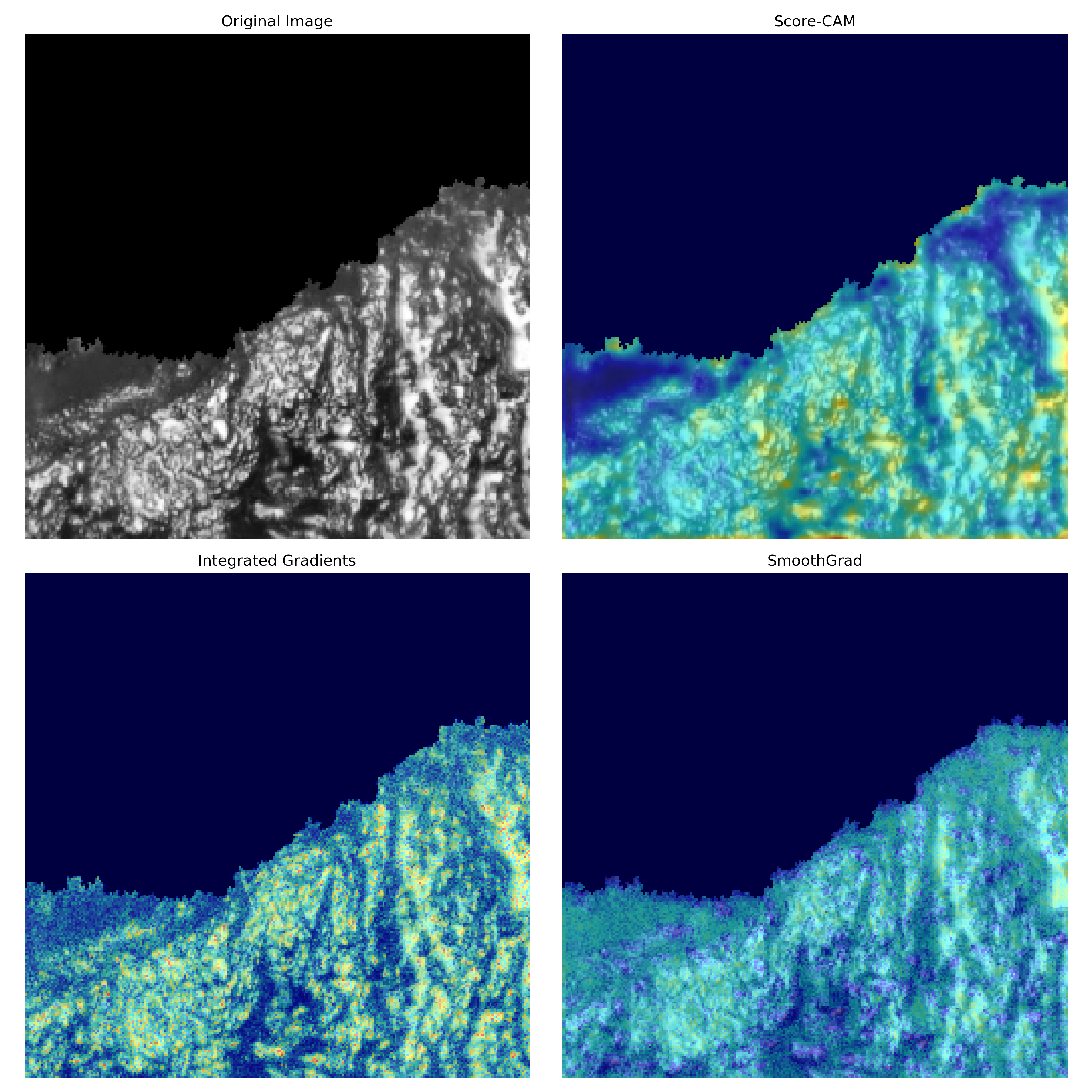

Supplement: Supplementary file 1 — Supplementary Material 1 [file 41598_2025_18179_MOESM1_ESM.tar › supplementary_material_resubmit1/Supplementary Figure S4/saliency maps/custom_CNN/100x_1000_16/wood_SW_1000_1_area_4_area_1_x100_1_quadrant_1.tif_visualization.png]

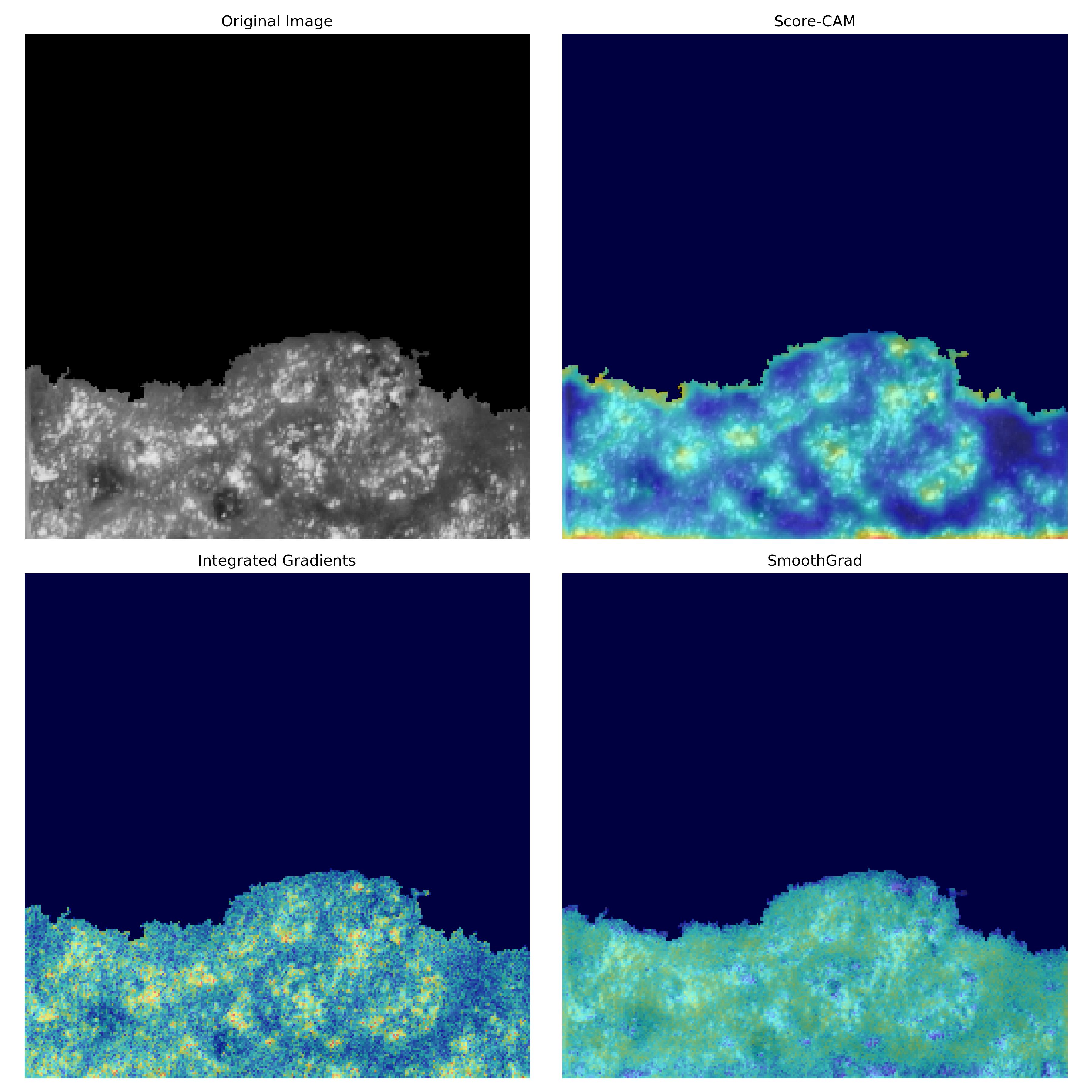

Supplement: Supplementary file 1 — Supplementary Material 1 [file 41598_2025_18179_MOESM1_ESM.tar › supplementary_material_resubmit1/Supplementary Figure S4/saliency maps/custom_CNN/100x_1000_16/wood_SW_1000_2_area_1_x100_1_quadrant_1.tif_visualization.png]

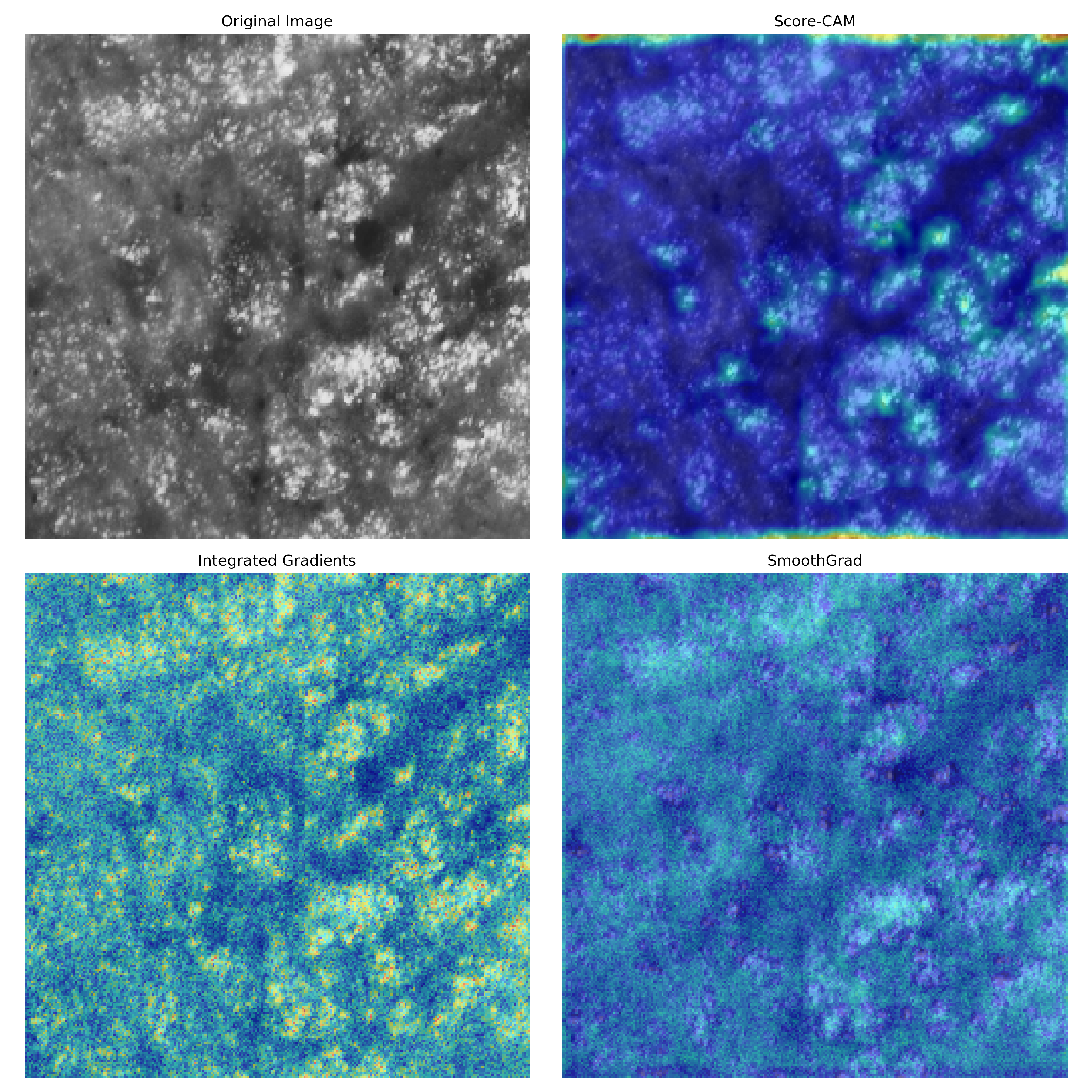

Supplement: Supplementary file 1 — Supplementary Material 1 [file 41598_2025_18179_MOESM1_ESM.tar › supplementary_material_resubmit1/Supplementary Figure S4/saliency maps/custom_CNN/100x_1000_16/wood_SW_1000_2_area_1_x100_1_quadrant_4.tif_visualization.png]

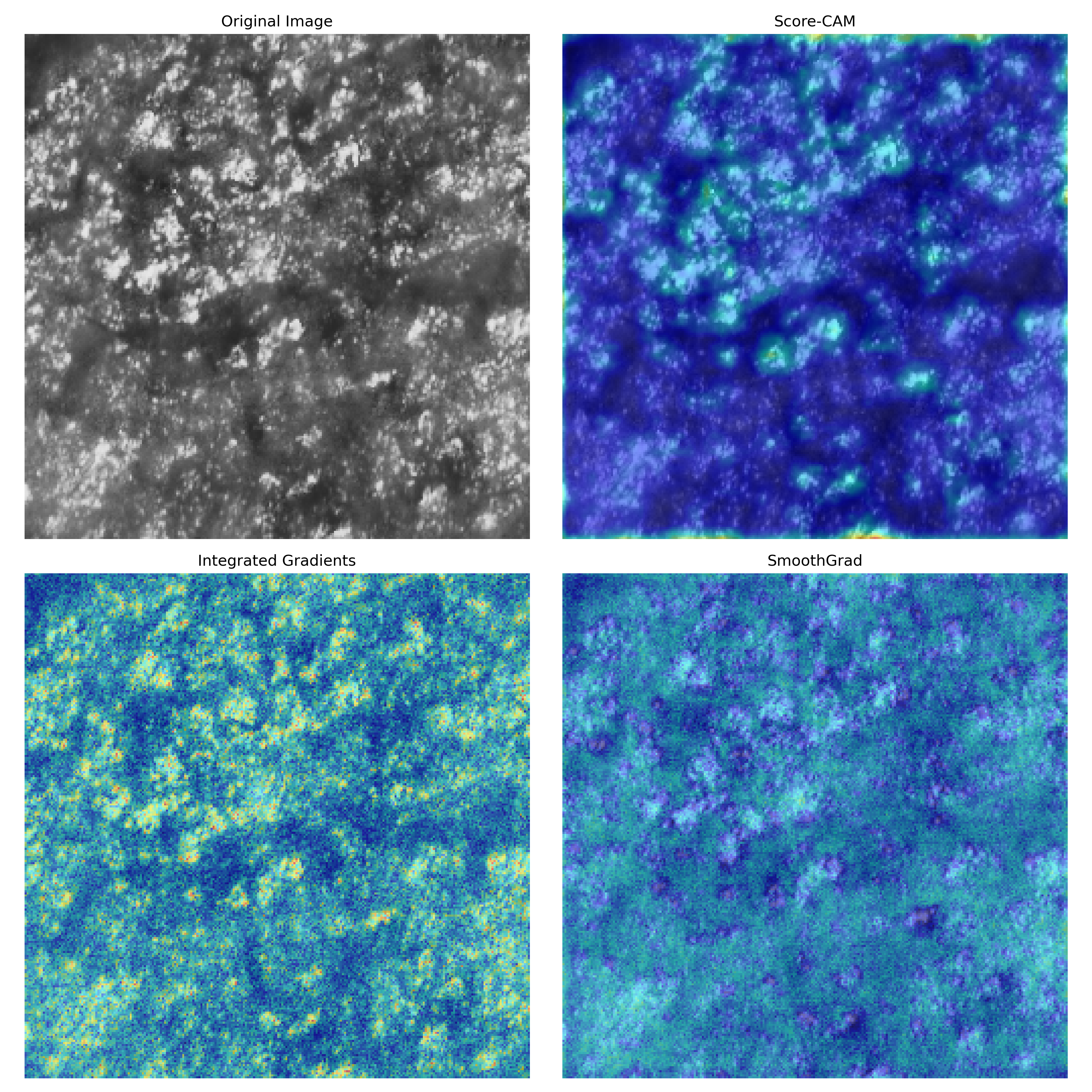

Supplement: Supplementary file 1 — Supplementary Material 1 [file 41598_2025_18179_MOESM1_ESM.tar › supplementary_material_resubmit1/Supplementary Figure S4/saliency maps/custom_CNN/100x_1000_16/wood_SW_1000_2_area_1_x100_1_quadrant_5.tif_visualization.png]

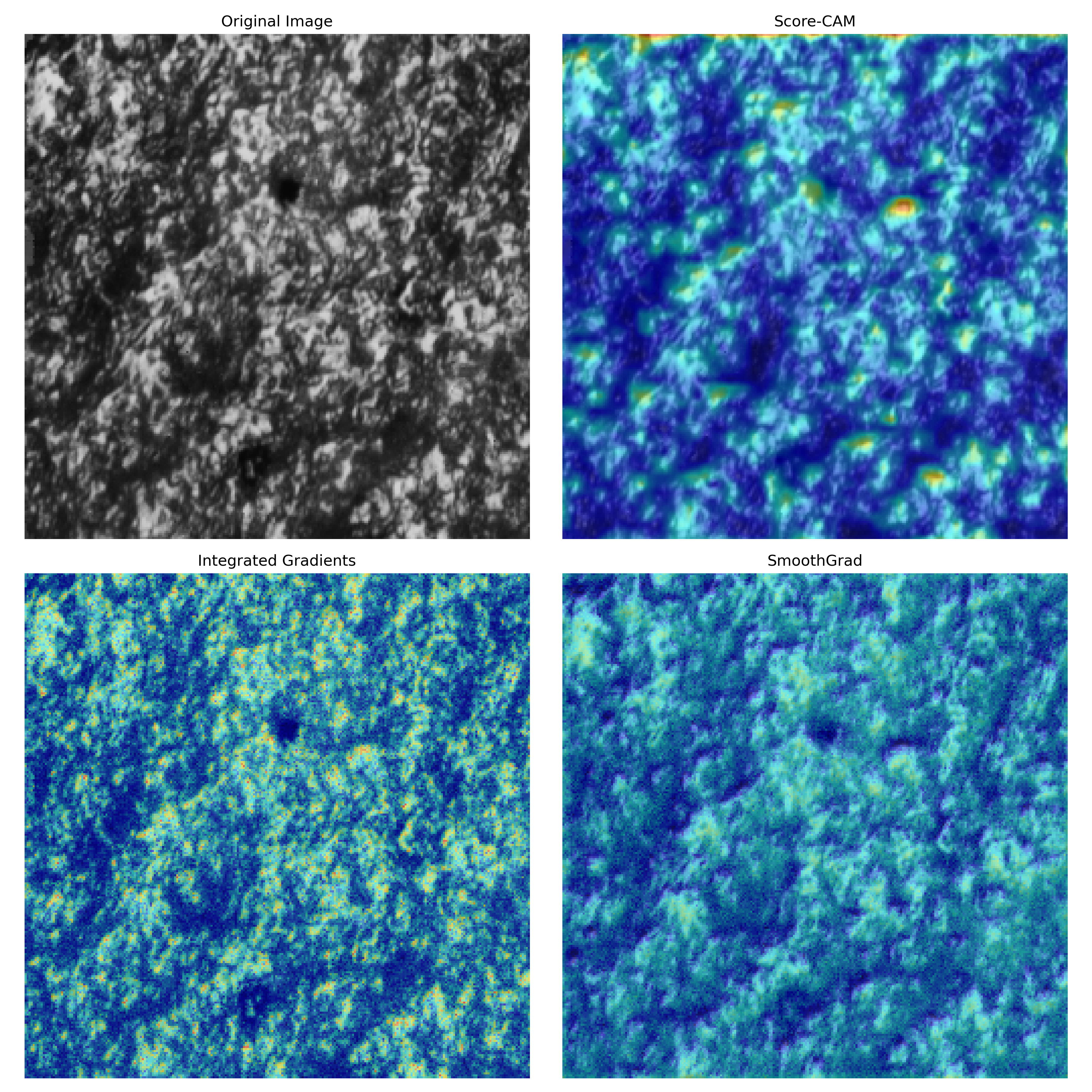

Supplement: Supplementary file 1 — Supplementary Material 1 [file 41598_2025_18179_MOESM1_ESM.tar › supplementary_material_resubmit1/Supplementary Figure S4/saliency maps/custom_CNN/x200_1000_16/bone_chichaoua_flint_CT_1000_area_1_area_1_x200_1_quadrant_7.tif_visualization.png]

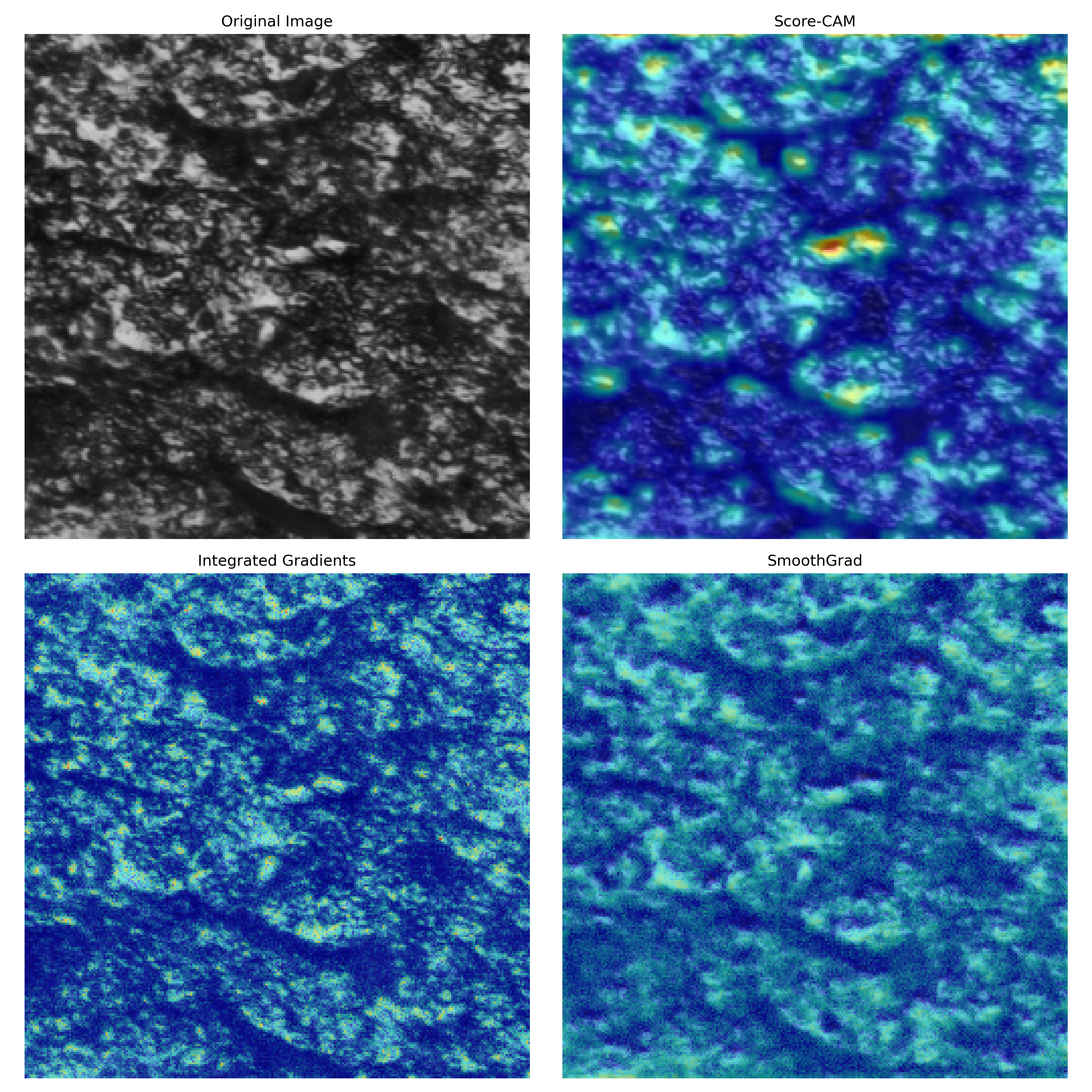

Supplement: Supplementary file 1 — Supplementary Material 1 [file 41598_2025_18179_MOESM1_ESM.tar › supplementary_material_resubmit1/Supplementary Figure S4/saliency maps/custom_CNN/x200_1000_16/bone_chichaoua_flint_CT_1000_area_1_area_2_x200_1_quadrant_12.tif_visualization.png]

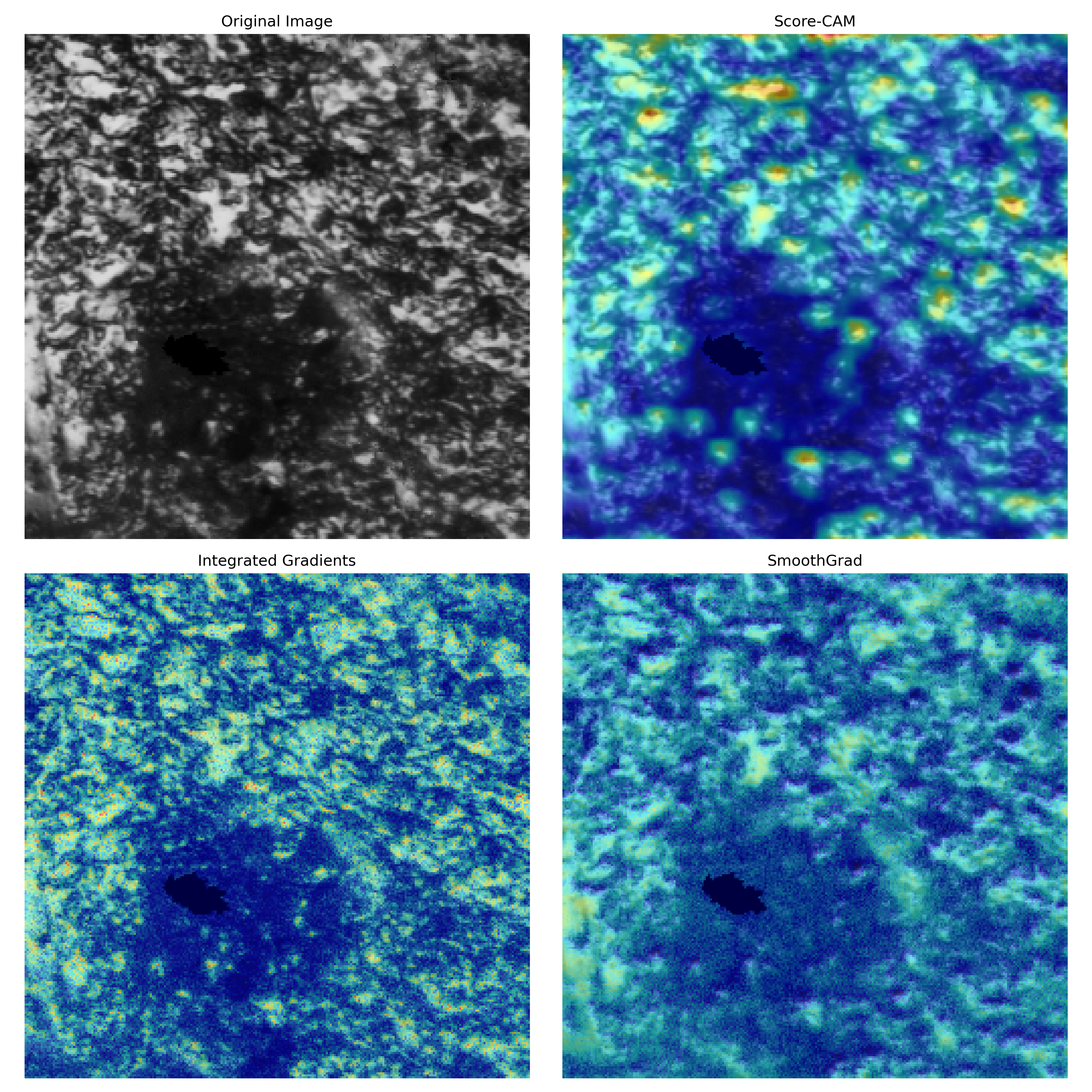

Supplement: Supplementary file 1 — Supplementary Material 1 [file 41598_2025_18179_MOESM1_ESM.tar › supplementary_material_resubmit1/Supplementary Figure S4/saliency maps/custom_CNN/x200_1000_16/bone_chichaoua_flint_CT_1000_area_1_area_2_x200_1_quadrant_8.tif_visualization.png]

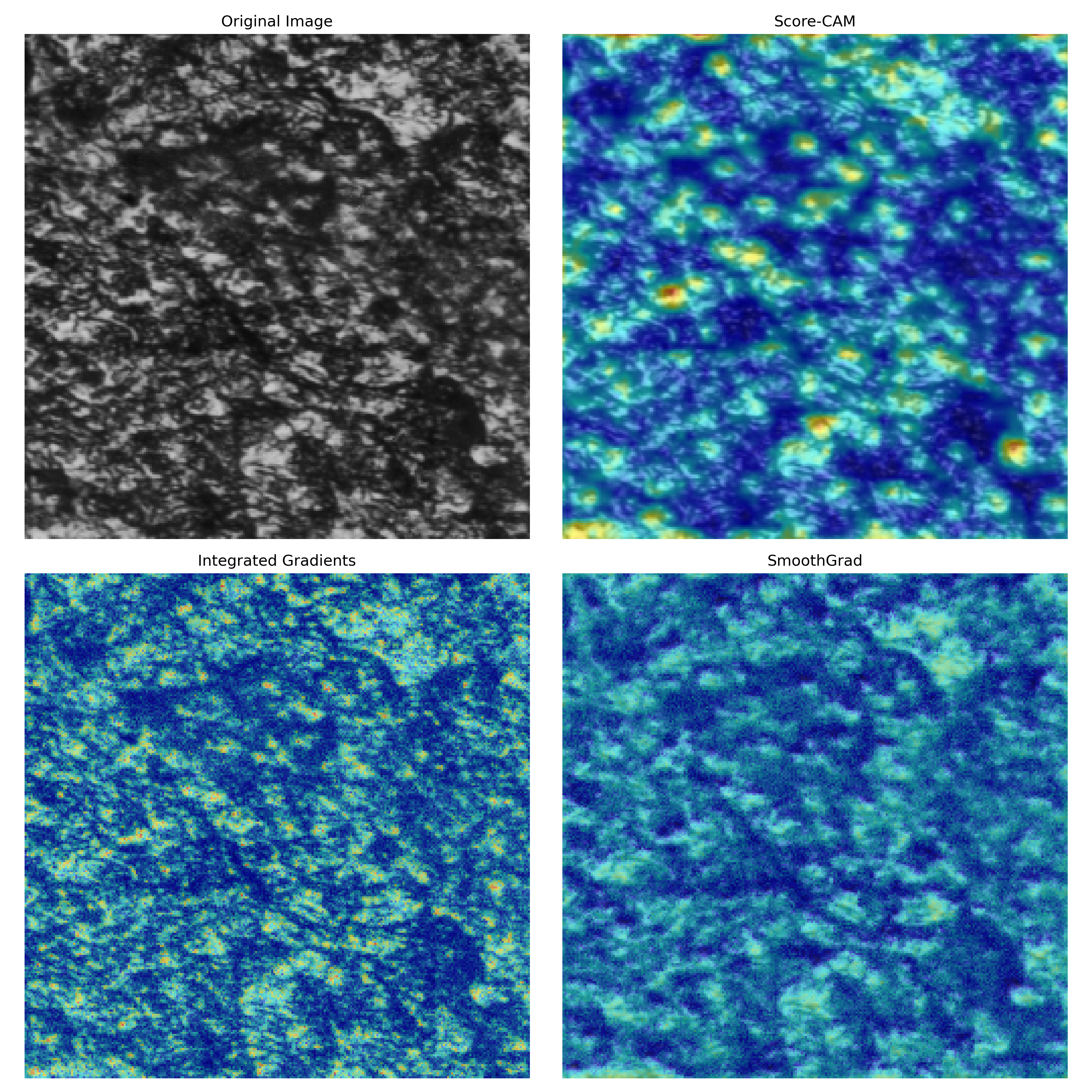

Supplement: Supplementary file 1 — Supplementary Material 1 [file 41598_2025_18179_MOESM1_ESM.tar › supplementary_material_resubmit1/Supplementary Figure S4/saliency maps/custom_CNN/x200_1000_16/bone_chichaoua_flint_CT_1000_area_1_area_2_x200_1_quadrant_9.tif_visualization.png]

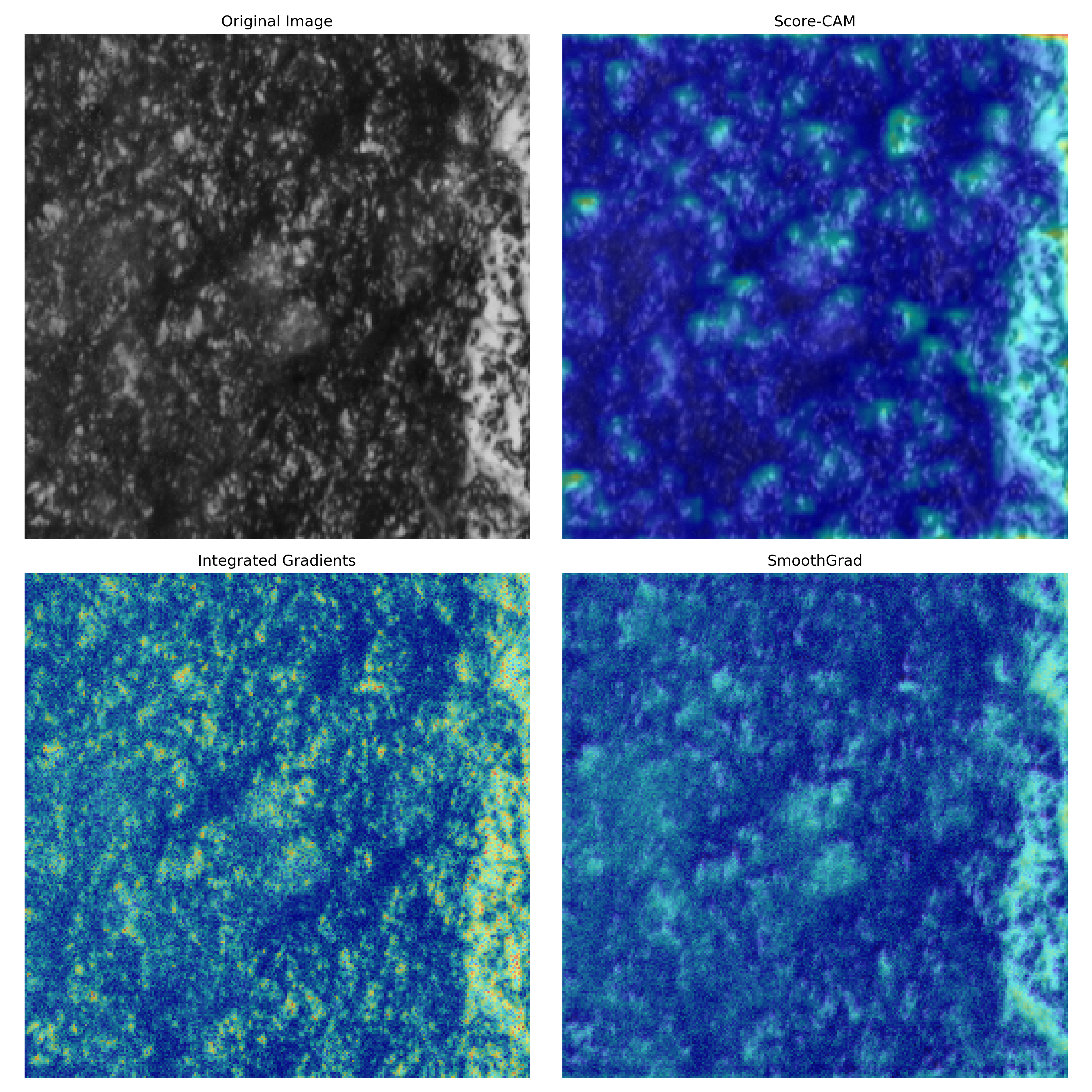

Supplement: Supplementary file 1 — Supplementary Material 1 [file 41598_2025_18179_MOESM1_ESM.tar › supplementary_material_resubmit1/Supplementary Figure S4/saliency maps/custom_CNN/x200_1000_16/bone_chichaoua_flint_CT_1000_area_2_area_2_x200_1_quadrant_10.tif_visualization.png]

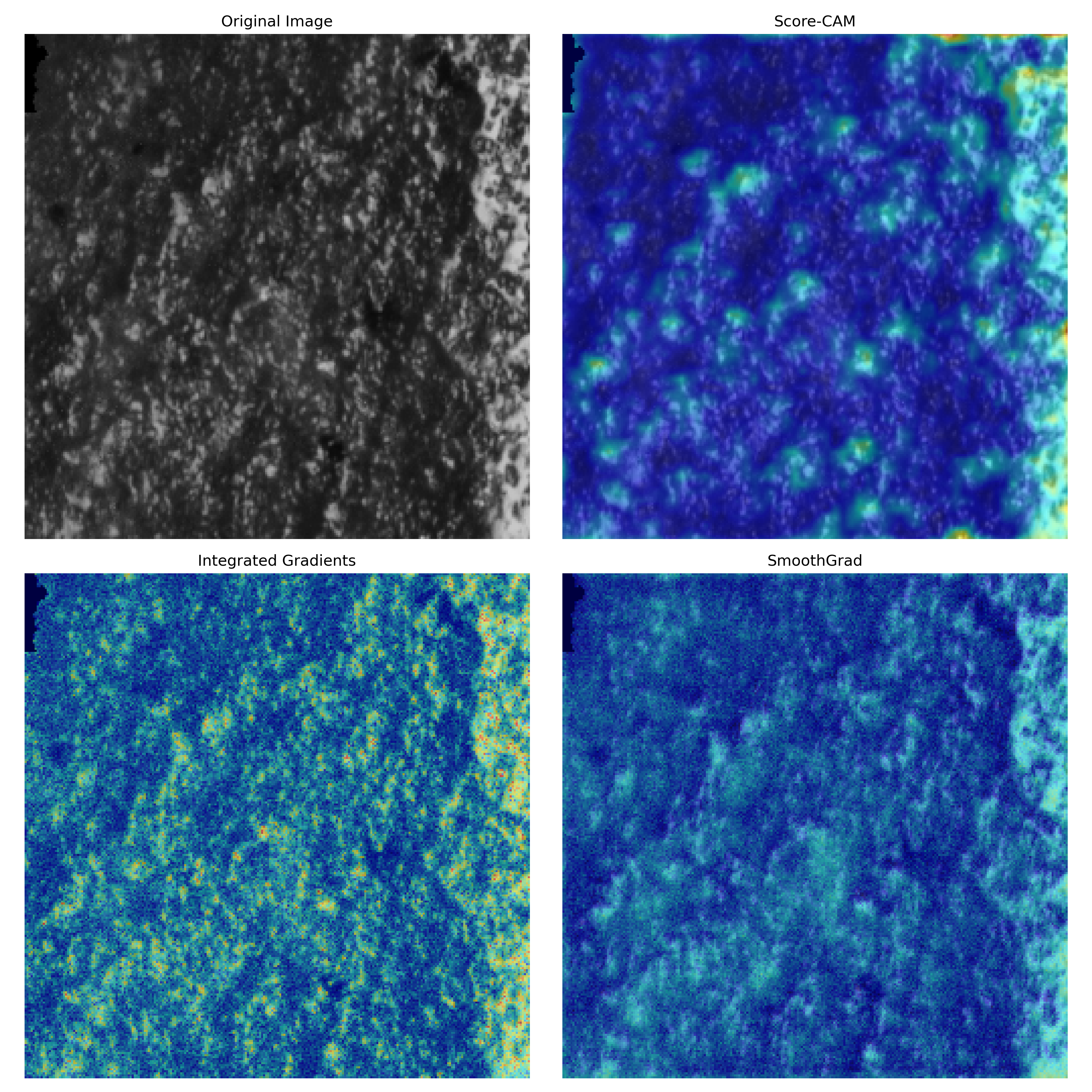

Supplement: Supplementary file 1 — Supplementary Material 1 [file 41598_2025_18179_MOESM1_ESM.tar › supplementary_material_resubmit1/Supplementary Figure S4/saliency maps/custom_CNN/x200_1000_16/bone_chichaoua_flint_CT_1000_area_2_area_2_x200_1_quadrant_6.tif_visualization.png]

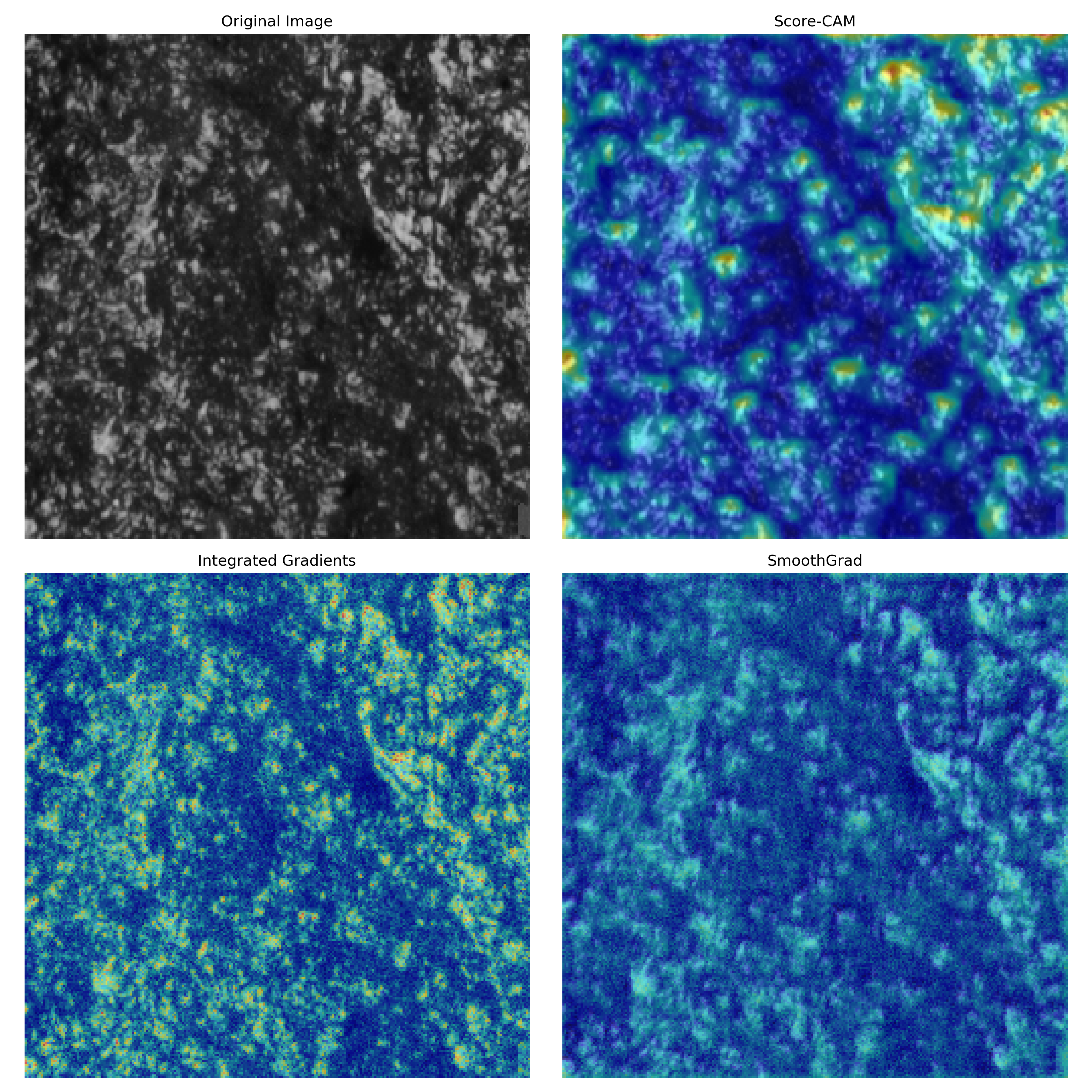

Supplement: Supplementary file 1 — Supplementary Material 1 [file 41598_2025_18179_MOESM1_ESM.tar › supplementary_material_resubmit1/Supplementary Figure S4/saliency maps/custom_CNN/x200_1000_16/bone_chichaoua_flint_CT_1000_area_2_area_2_x200_1_quadrant_8.tif_visualization.png]

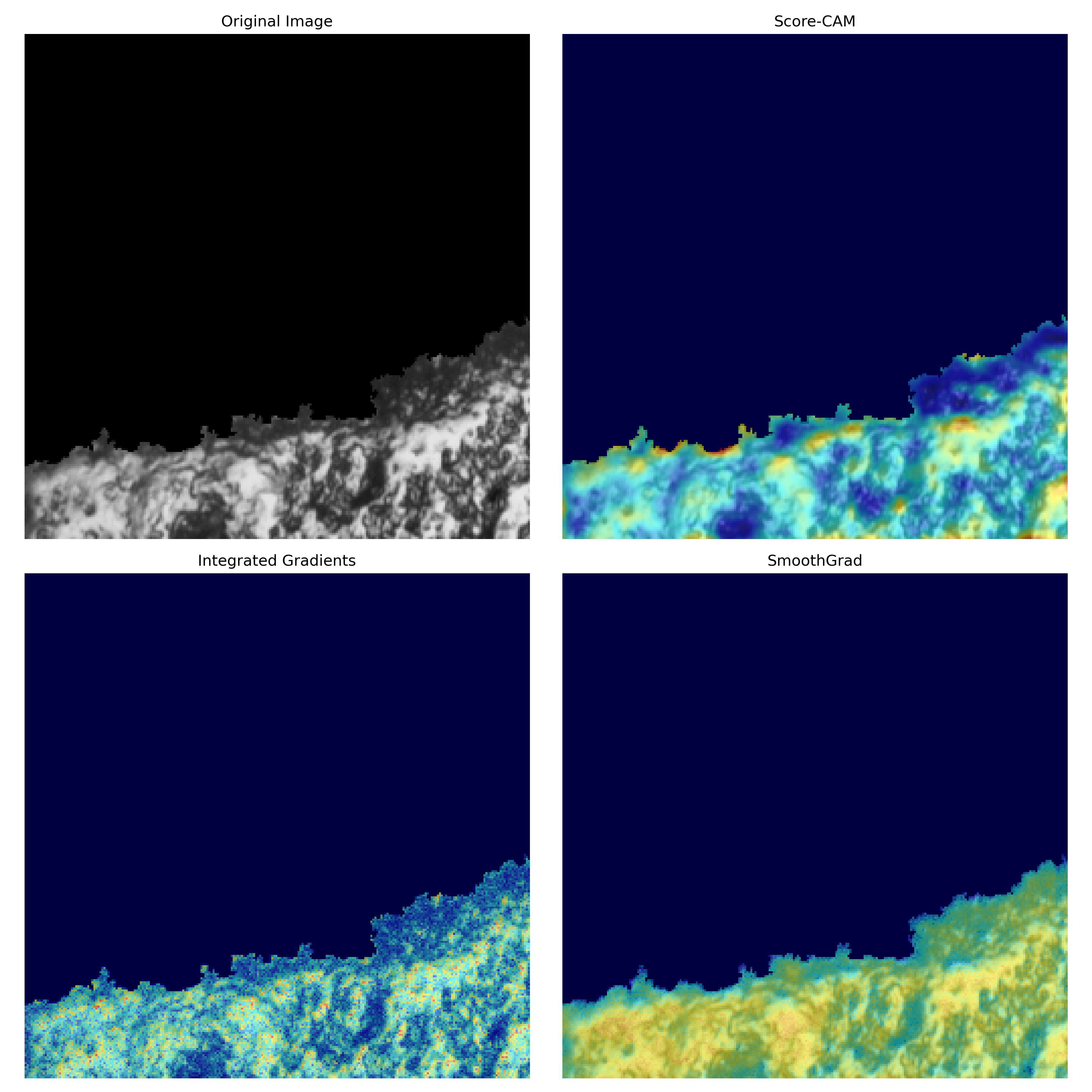

Supplement: Supplementary file 1 — Supplementary Material 1 [file 41598_2025_18179_MOESM1_ESM.tar › supplementary_material_resubmit1/Supplementary Figure S4/saliency maps/custom_CNN/x200_1000_16/bone_chichaoua_flint_CT_1000_area_3_area_1_x200_1_quadrant_1.tif_visualization.png]

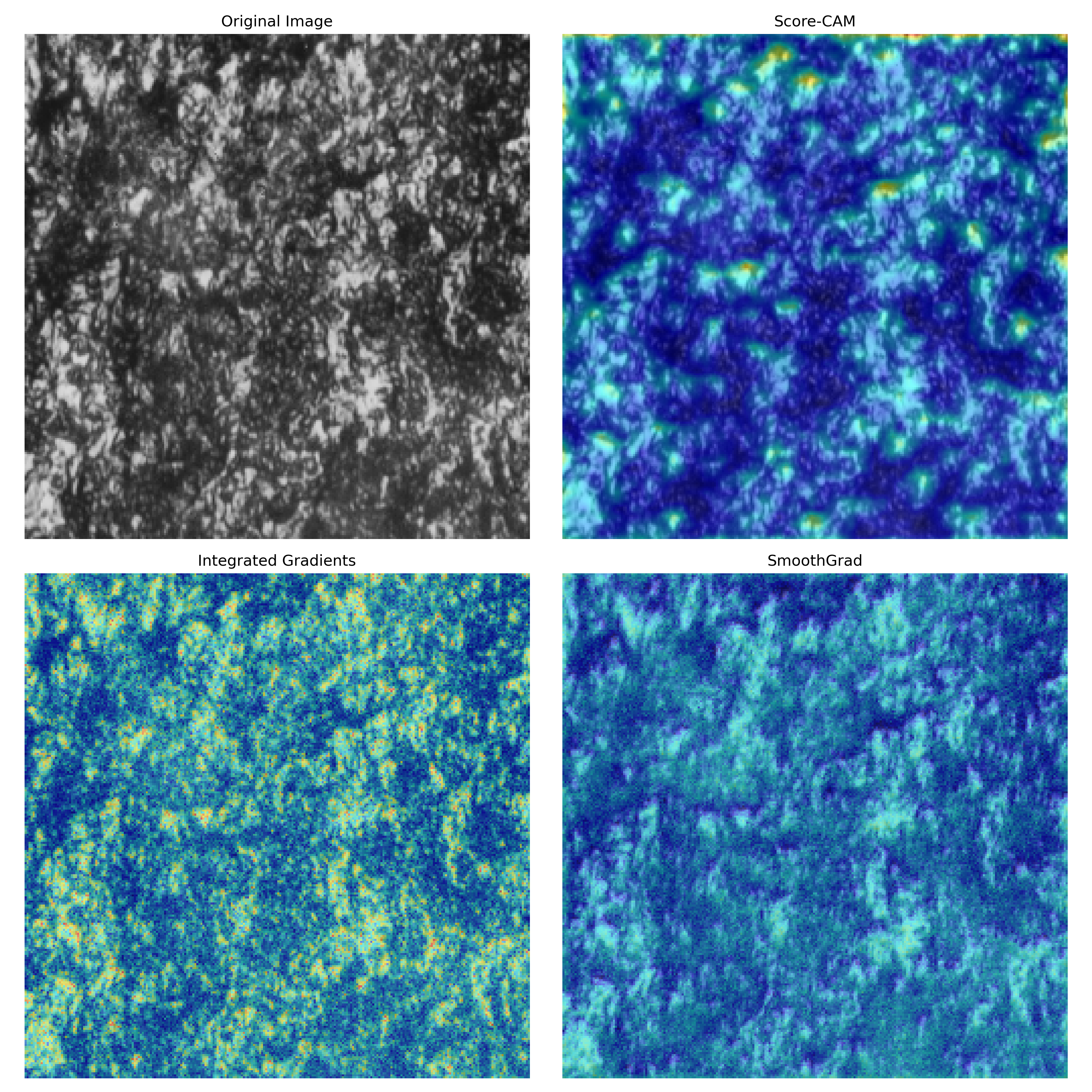

Supplement: Supplementary file 1 — Supplementary Material 1 [file 41598_2025_18179_MOESM1_ESM.tar › supplementary_material_resubmit1/Supplementary Figure S4/saliency maps/custom_CNN/x200_1000_16/bone_chichaoua_flint_CT_1000_area_3_area_1_x200_1_quadrant_11.tif_visualization.png]

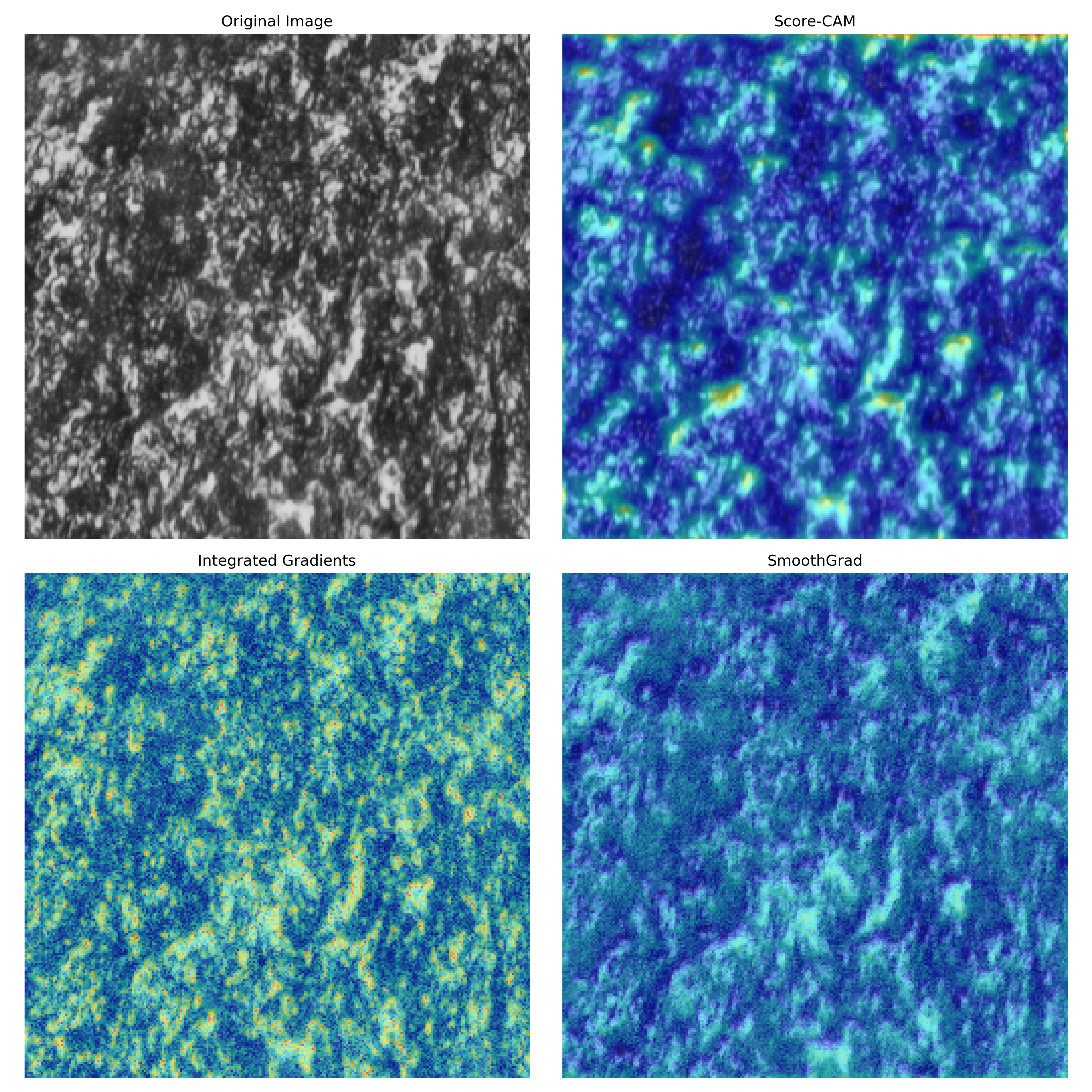

Supplement: Supplementary file 1 — Supplementary Material 1 [file 41598_2025_18179_MOESM1_ESM.tar › supplementary_material_resubmit1/Supplementary Figure S4/saliency maps/custom_CNN/x200_1000_16/bone_chichaoua_flint_CT_1000_area_3_area_1_x200_1_quadrant_14.tif_visualization.png]

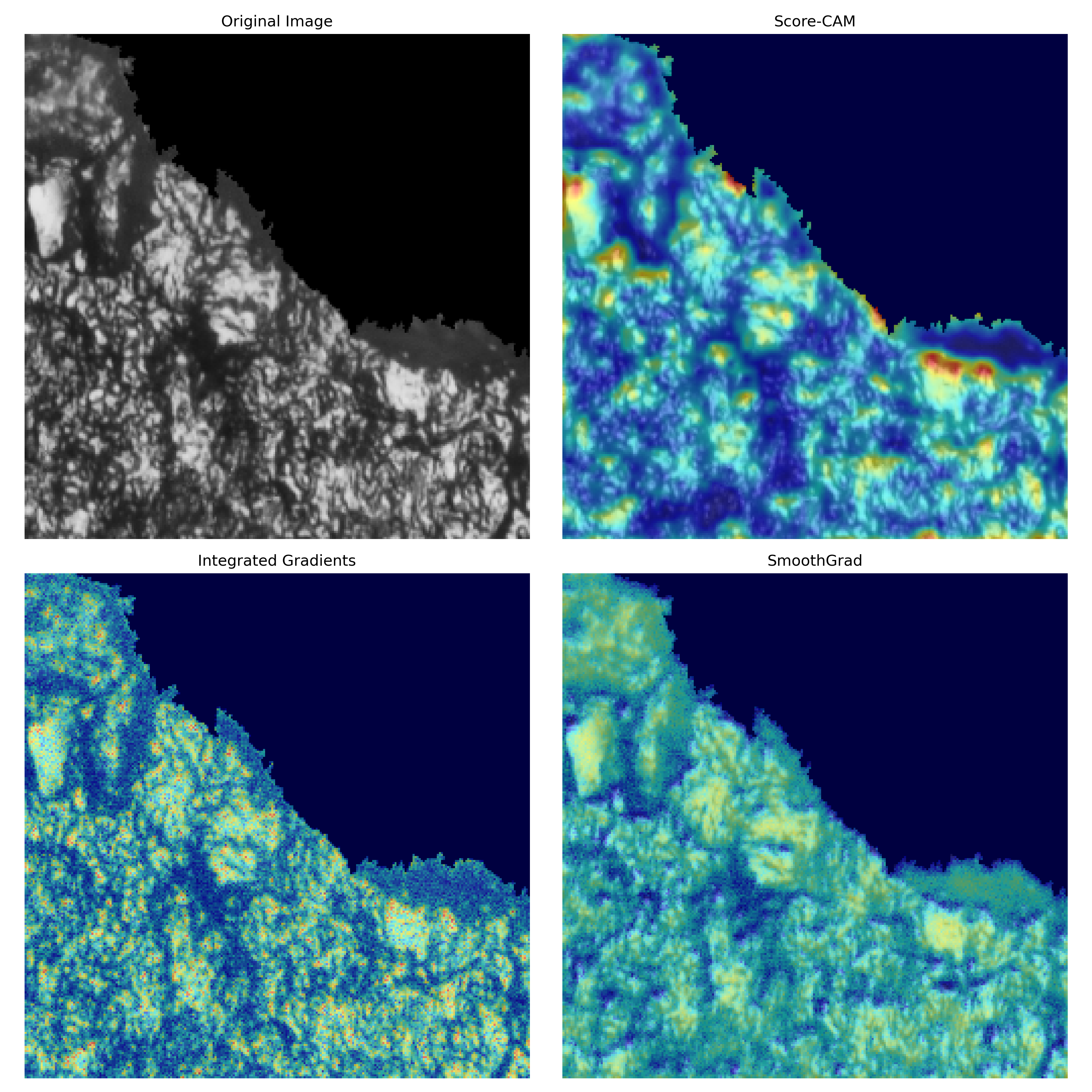

Supplement: Supplementary file 1 — Supplementary Material 1 [file 41598_2025_18179_MOESM1_ESM.tar › supplementary_material_resubmit1/Supplementary Figure S4/saliency maps/custom_CNN/x200_1000_16/bone_chichaoua_flint_CT_1000_area_3_area_2_x200_1_quadrant_4.tif_visualization.png]

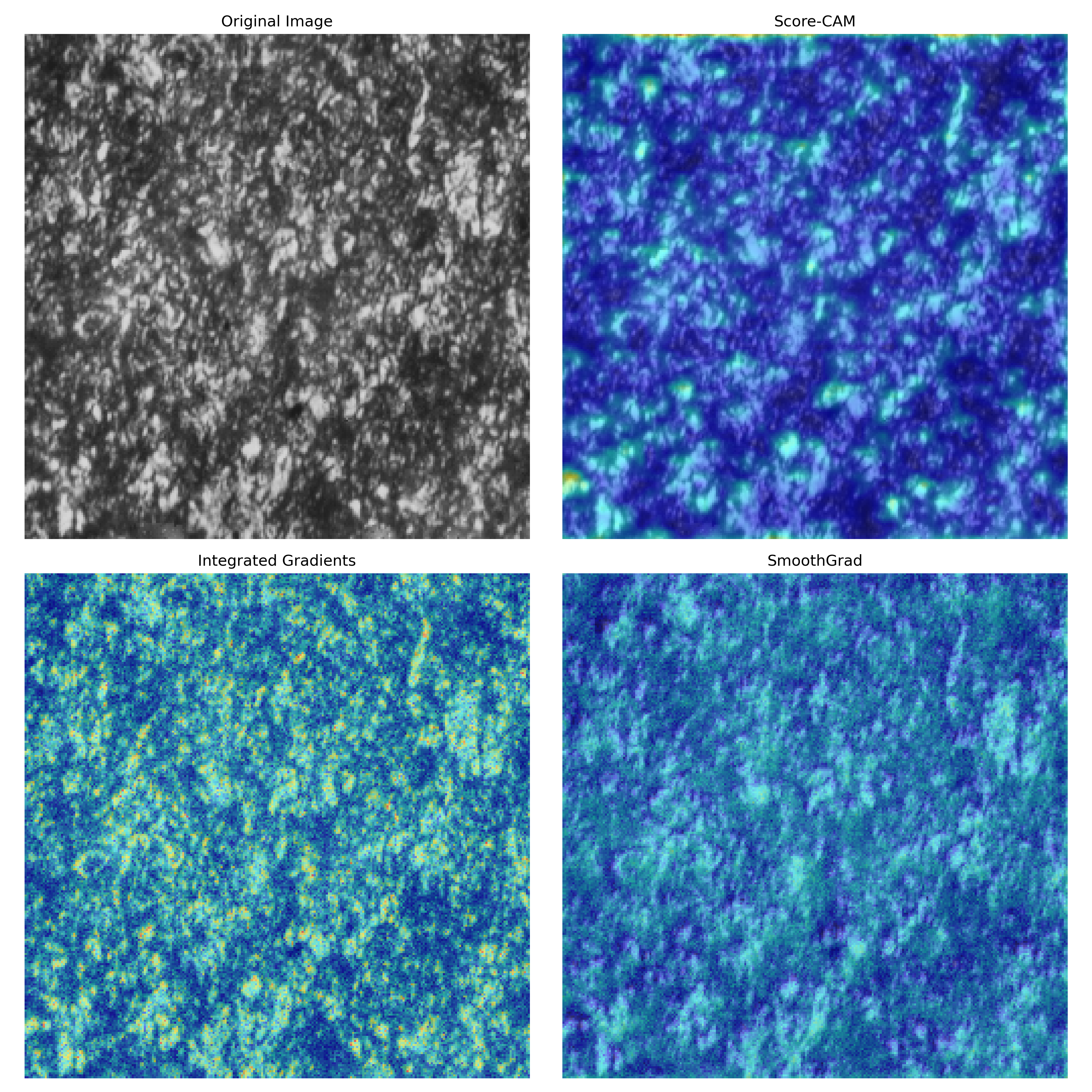

Supplement: Supplementary file 1 — Supplementary Material 1 [file 41598_2025_18179_MOESM1_ESM.tar › supplementary_material_resubmit1/Supplementary Figure S4/saliency maps/custom_CNN/x200_1000_16/bone_chichaoua_flint_CT_1000_area_3_area_2_x200_1_quadrant_5.tif_visualization.png]

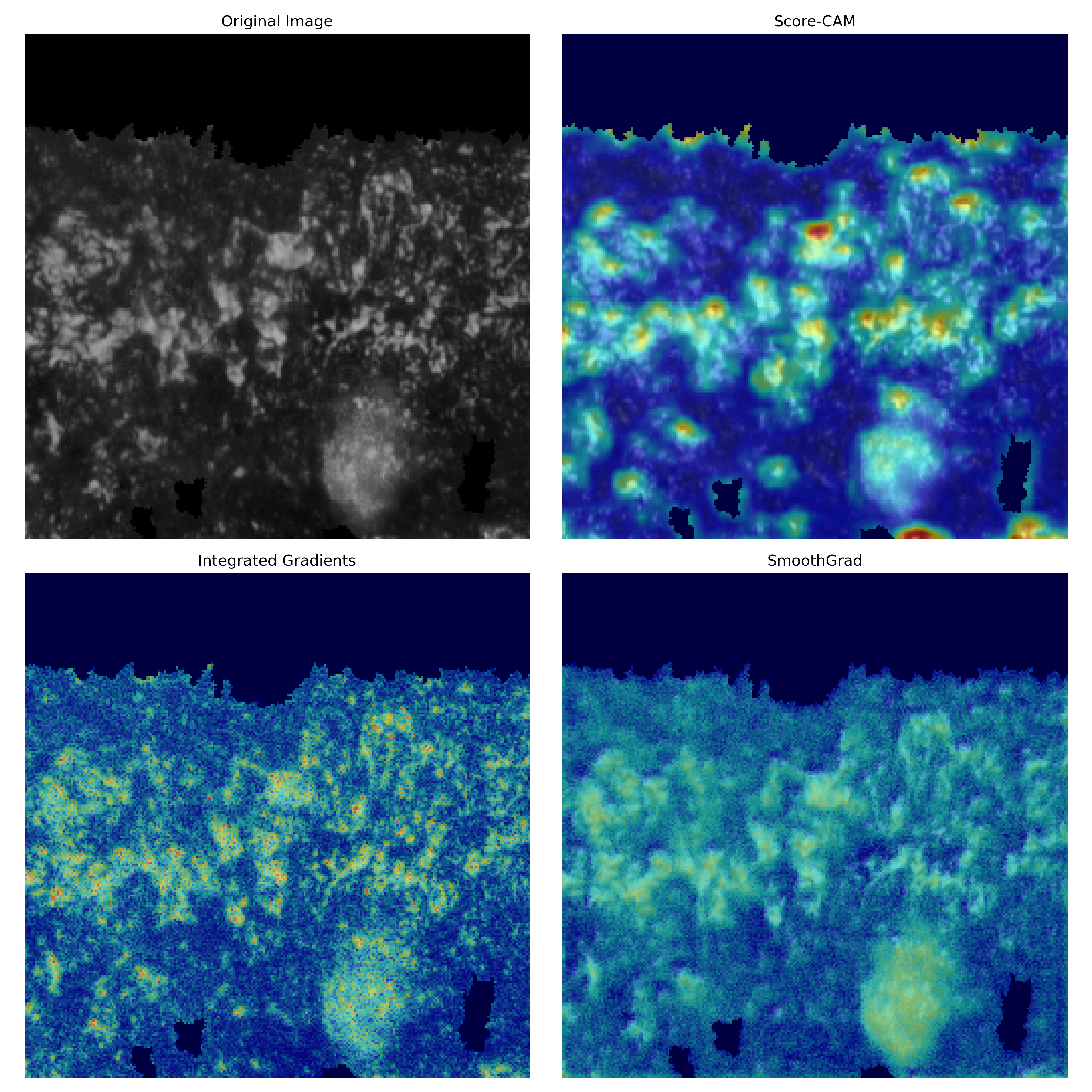

Supplement: Supplementary file 1 — Supplementary Material 1 [file 41598_2025_18179_MOESM1_ESM.tar › supplementary_material_resubmit1/Supplementary Figure S4/saliency maps/custom_CNN/x200_1000_16/bone_chichaoua_flint_CT_1000_area_4_area_2_x200_1_quadrant_3.tif_visualization.png]

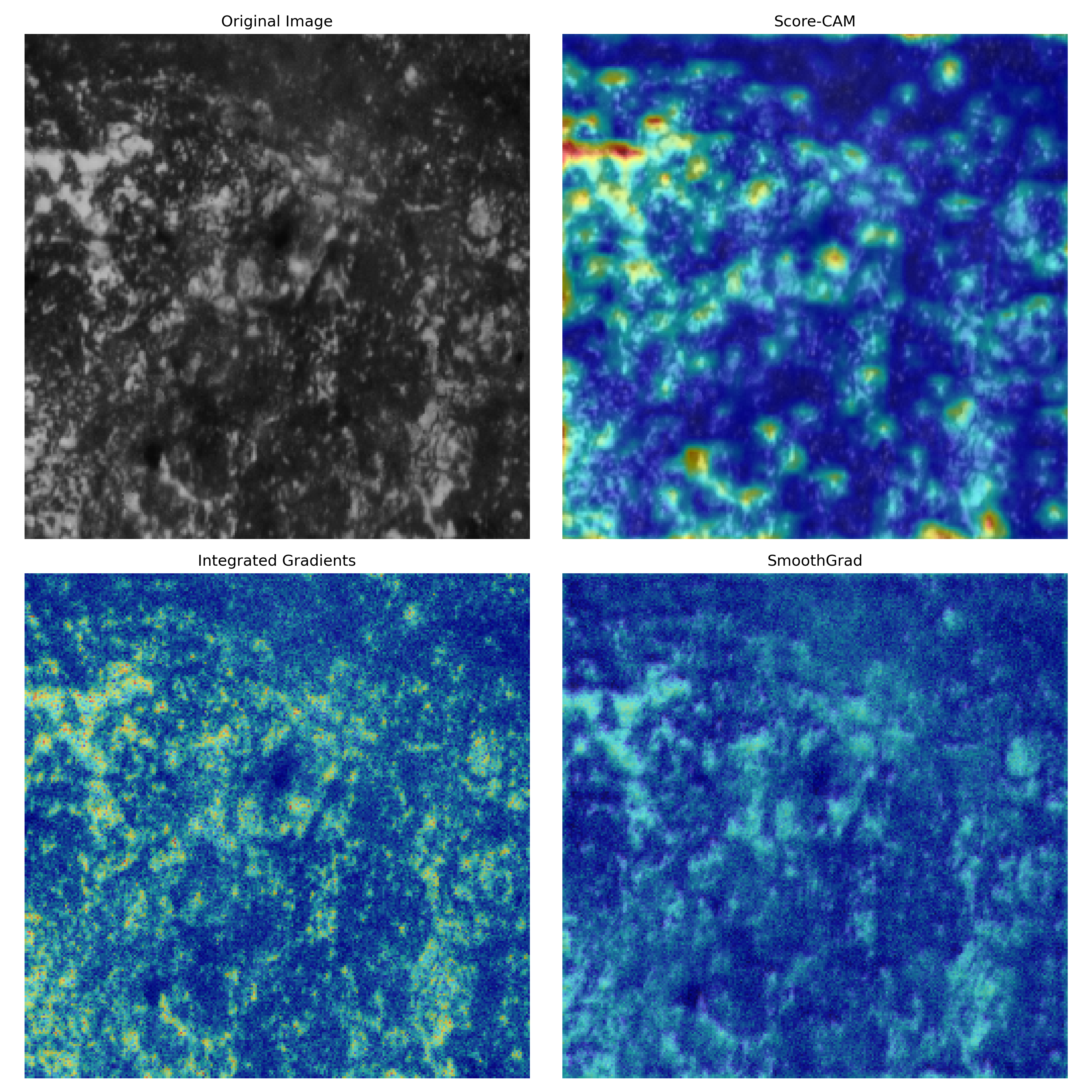

Supplement: Supplementary file 1 — Supplementary Material 1 [file 41598_2025_18179_MOESM1_ESM.tar › supplementary_material_resubmit1/Supplementary Figure S4/saliency maps/custom_CNN/x200_1000_16/bone_chichaoua_flint_CT_1000_area_4_area_2_x200_1_quadrant_6.tif_visualization.png]

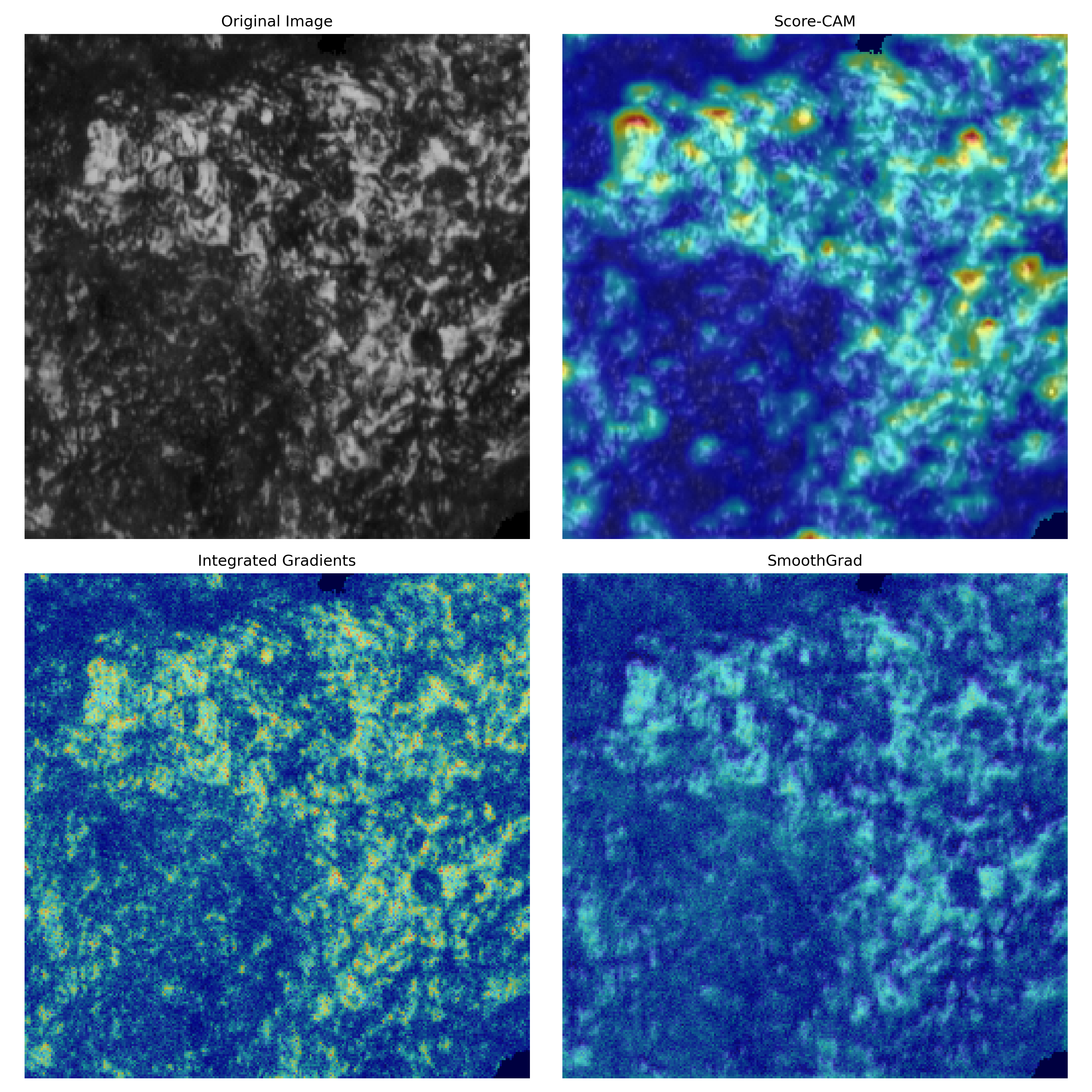

Supplement: Supplementary file 1 — Supplementary Material 1 [file 41598_2025_18179_MOESM1_ESM.tar › supplementary_material_resubmit1/Supplementary Figure S4/saliency maps/custom_CNN/x200_1000_16/bone_chichaoua_flint_CT_1000_area_4_area_2_x200_1_quadrant_7.tif_visualization.png]

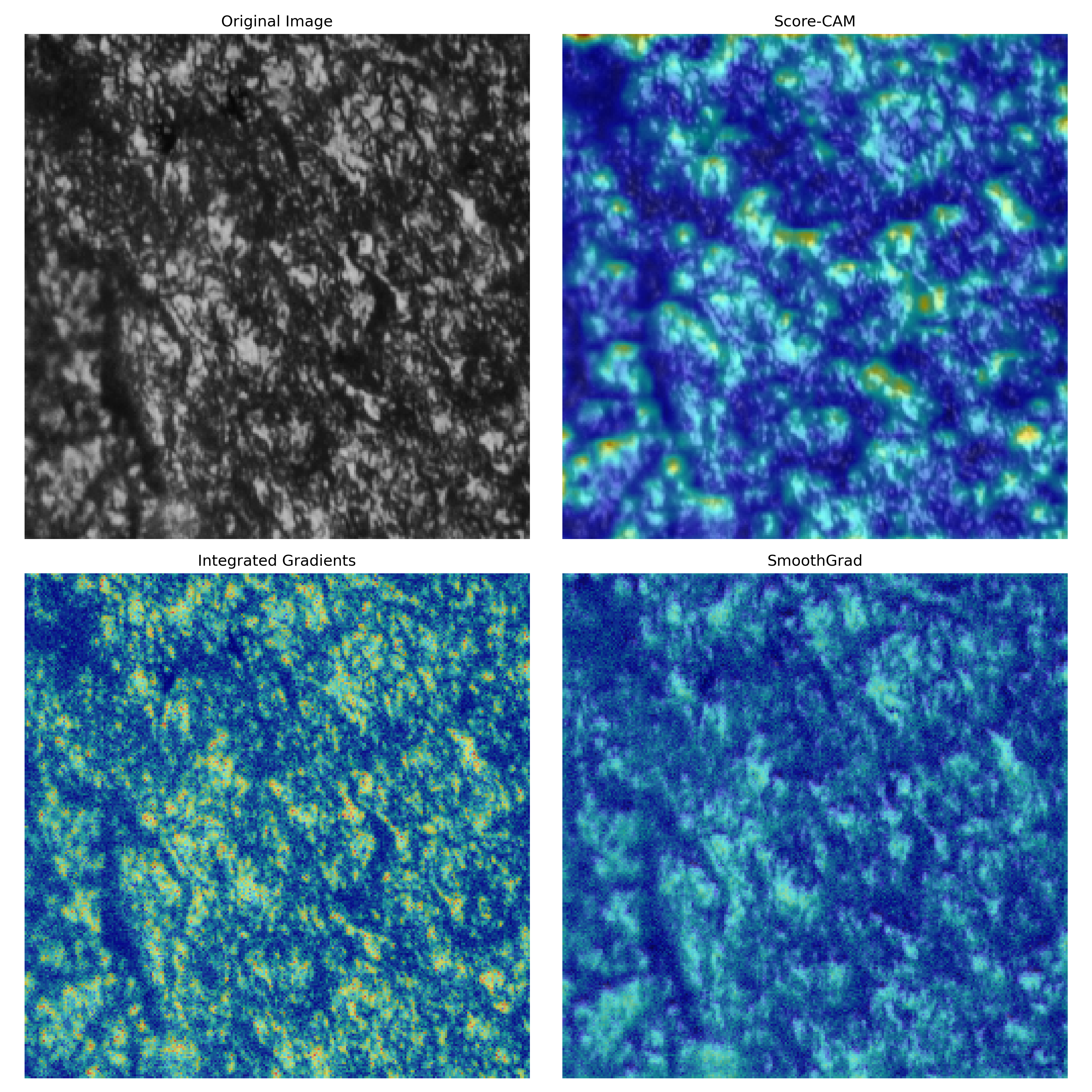

Supplement: Supplementary file 1 — Supplementary Material 1 [file 41598_2025_18179_MOESM1_ESM.tar › supplementary_material_resubmit1/Supplementary Figure S4/saliency maps/custom_CNN/x200_1000_16/bone_chichaoua_flint_CT_1000_area_5_area_1_x200_1_quadrant_13.tif_visualization.png]

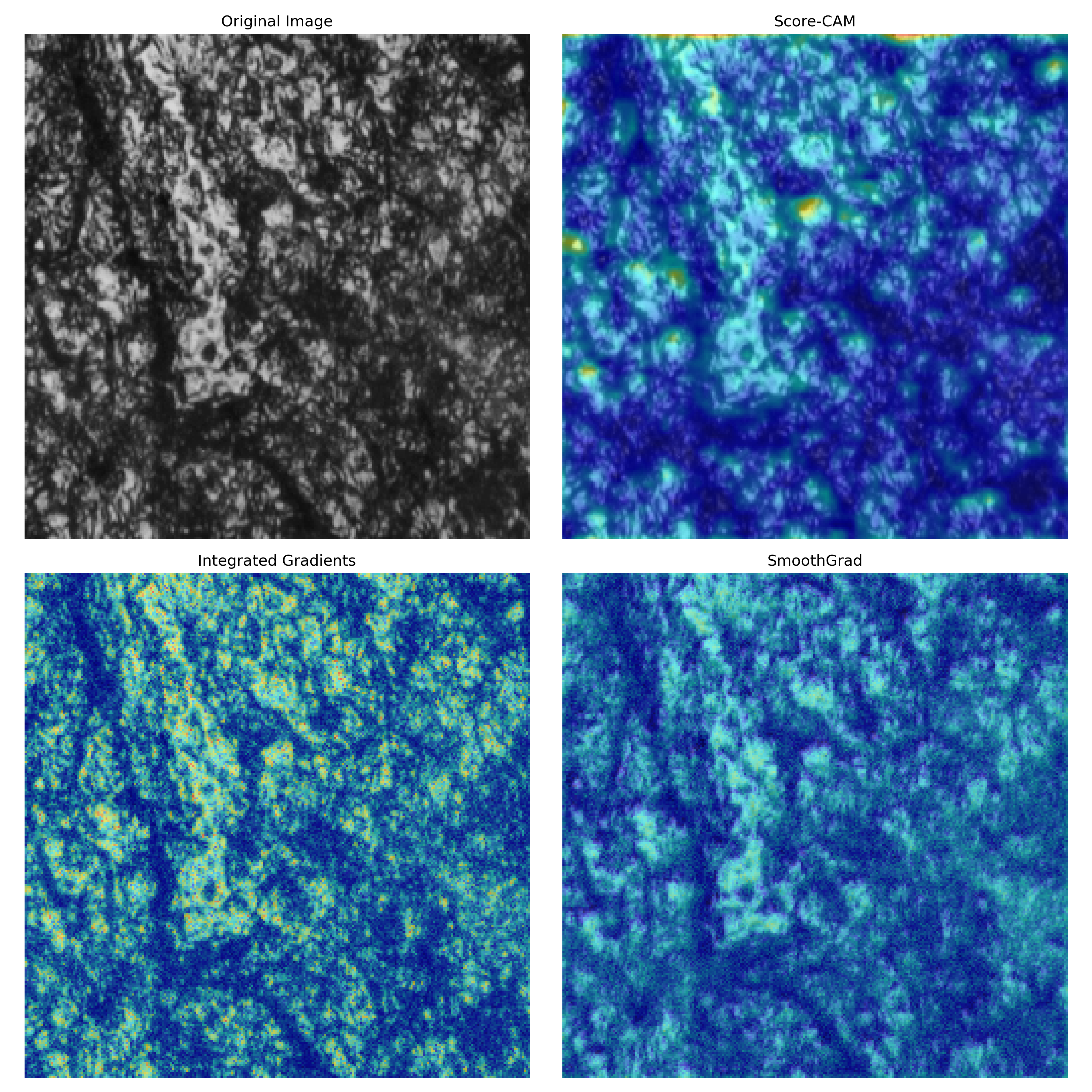

Supplement: Supplementary file 1 — Supplementary Material 1 [file 41598_2025_18179_MOESM1_ESM.tar › supplementary_material_resubmit1/Supplementary Figure S4/saliency maps/custom_CNN/x200_1000_16/bone_chichaoua_flint_CT_1000_area_5_area_1_x200_1_quadrant_8.tif_visualization.png]

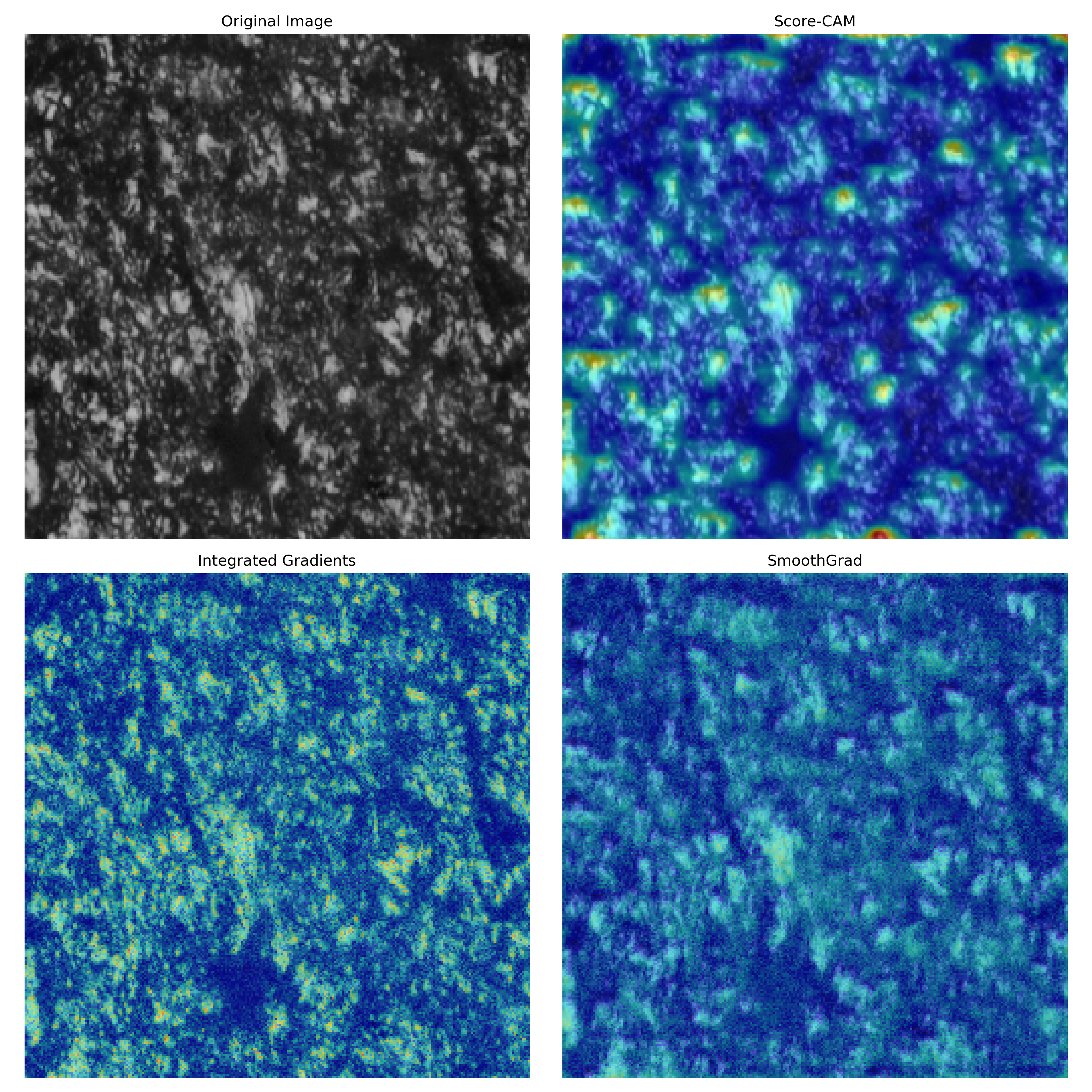

Supplement: Supplementary file 1 — Supplementary Material 1 [file 41598_2025_18179_MOESM1_ESM.tar › supplementary_material_resubmit1/Supplementary Figure S4/saliency maps/custom_CNN/x200_1000_16/bone_chichaoua_flint_CT_1000_area_5_area_1_x200_1_quadrant_9.tif_visualization.png]

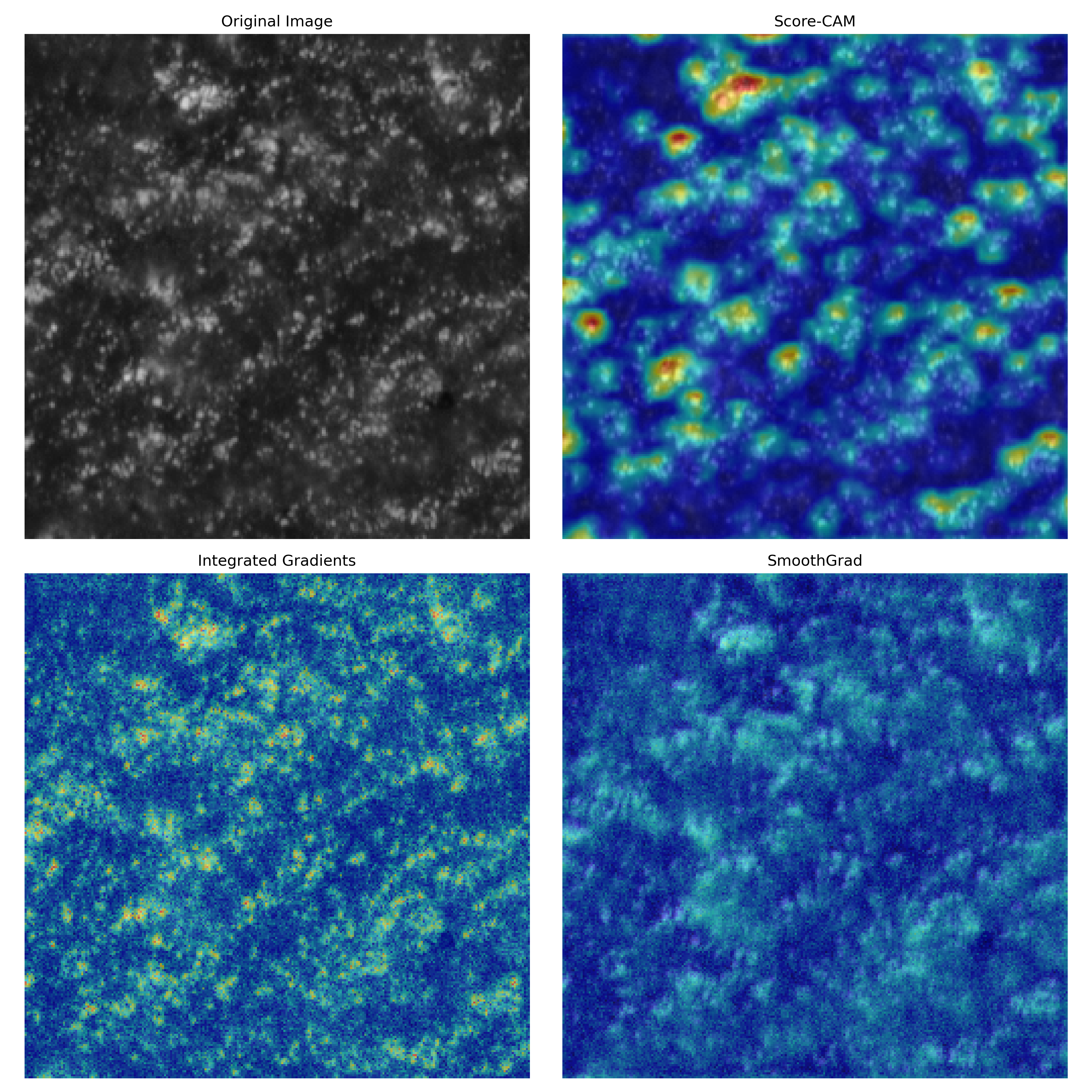

Supplement: Supplementary file 1 — Supplementary Material 1 [file 41598_2025_18179_MOESM1_ESM.tar › supplementary_material_resubmit1/Supplementary Figure S4/saliency maps/custom_CNN/x200_1000_16/bone_chichaoua_flint_SCB_1000_2_area_1_maybe_area_1_x200_1_quadrant_7.tif_visualization.png]
