# Supplementary material for: Classifying polish in use-wear analysis with convolutional neural networks
Source: Sci Rep. 2025 Oct 22;15:36834. doi: 10.1038/s41598-025-18179-4 (PMC12546859; doi:10.1038/s41598-025-18179-4)
Supplement: Supplementary file 1 — Supplementary Material 1 [file 41598_2025_18179_MOESM1_ESM.tar › supplementary_material_resubmit1/Supplementary Information.docx]

**Supplementary Information**

- **Supplementary Data**
  - **S1:** Two preprocessed and cropped subsets of the dataset are provided in the Zenodo repository to facilitate the reproducibility of the current analysis [see Data Availability]. The subsets are organized into two folders: ‘squares_x200_1000_9’ (10x objective, 1000 strokes, and 9 patch size) and ‘squares_x200_2000_16’ (20x objective, 2000 strokes, and 16 patch size). The filenames of the images are standardized to encode the contact material, activity, number of strokes, area, subarea (a single tool may have multiple areas of polish), and objective.
    - e.g., bone_chichaoua_flint_SC_1000_1_area_2_area_2_x100_1_quadrant_6.tif Detailed Breakdown:
- *bone*: The contact material (e.g., bone, antler, wood).
- *chichaoua_flint*: The raw material of the experimental lithic tool used.
- SC: The activity conducted (e.g., SC for scraping, SW for sawing, CT for cutting).
- *1000*: The number of strokes applied during the experiment.
- *1_area_2_area_2:* This part represents the areas and subareas of polish captured in the image:
  - The 1 indicates that there is one primary area being analyzed by the use-wear analyst.
  - area_2_area_2 indicates that this area includes 2 subareas, each of which can contain additional subareas.
- *x100*: The optical magnification, where x100 is for 10x objective and x200 is for 20x objective.
- *1:* Ensures that this is a unique file without duplication.
- *quadrant_6*: The identifier of the patch or region of the sample captured in the image (e.g., quadrant 6 of the area).
- **Supplementary Tables**
  - **S1:** Report on the confusion matrices for convolutional neural network (CNN) model, including precision, recall, and F1-scores.
  - **S2:** Standard errors and scatterplots of validation accuracy across 20 re-runs with different random seeds.
  - **S3:** Report on the results of each model, including accuracy, loss (training/validation), and ROC score. Also provided are the number of images in the training set (before and after augmentation), as well as the number of images in the testing and validation sets.
  - **S4:** Count of images by contact material, objective and number of strokes.
  - **S5:** Summary of experimental design and data acquisition**.**
  - **S6:** Report on the effect of the number of unfreezing layers.
- **Supplementary Figures**
  - **S1:** Boxplot illustrating the impact of contact material, objective and patch size on polish classification based on model type
  - **S2:** Boxplot illustrating the impact of model type on polish classification, based on the number of strokes and patch size, using F1-score values.
  - **S3:** Example of background and unfocused area masking in the dataset. The images on the left (a, c, e) show the original samples and those on the right (b, d, f) display their cropped versions.
  - **S4:** All results, including evaluation metrics (learning curves, confusion matrices, ROC curves) and saliency maps for all models, are available in the Zenodo repository [link] in the folder ‘Supplementary Figures S4’. This folder contains two subfolders: ‘evaluation_metrics’ and ‘saliency_maps’. Within each subfolder, there are further directories for ‘custom_CNN’ and ‘ResNet50_CNN’, each containing folders for the different models trained on various subsets of the dataset, e.g., ‘x100_1000_16’.
